# Meta-analysis of factors for osteonecrosis in systemic lupus erythematosus: integration of comprehensive literatures and multicenter databases

**DOI:** 10.3389/fimmu.2026.1679237

**Published:** 2026-07-02

**Authors:** Rui-Cen Li, Lin-Chong Su, An-Fang Huang, Wang-Dong Xu, Xiao-Yan Liu

**Affiliations:** 1 Health Management Center, West China Hospital, Sichuan University, Chengdu, Sichuan, China; 2 Hubei Provincial Key Laboratory of Occurrence and Intervention of Rheumatic Diseases, Affiliated Minda Hospital of Hubei Minzu University, Enshi, Hubei, China; 3 Department of Rheumatology and Immunology, Affiliated Minda Hospital of Hubei Minzu University, Enshi, Hubei, China; 4 Department of Rheumatology and Immunology, The Affiliated Hospital, Southwest Medical University, Luzhou, Sichuan, China; 5 Department of Evidence-Based Medicine, School of Public Health, Southwest Medical University, Luzhou, Sichuan, China

**Keywords:** anti-SSA/SSB, meta-analysis, osteonecrosis, risk factor, SLE

## Abstract

**Background:**

Osteonecrosis (ON) is a prevalent and severe complication in patients with systemic lupus erythematosus (SLE). However, the etiology and mechanism of SLE-ON have not been fully elucidated. This study aims to investigate the factors related to SLE-ON and provide a basis for early prevention and control.

**Methods:**

A comprehensive search of relevant literatures was conducted from PubMed, Ovid Medline, Web of Science, Embase, Cochrane Library, CNKI, Wanfang Data, and VIP Information databases up to 31 October 2025. According to the inclusion and exclusion criteria, 3,051 studies were examined, and quality of each study was assessed using the Newcastle–Ottawa scale (NOS). Meta-analysis methods discussed the association of the factors related to SLE-ON.

**Results:**

A total of 64 studies were finally included in the meta-analysis. For clinical features, factors such as neuropsychiatric lupus (OR = 1.652), hyperlipidemia (OR = 1.358), oral ulcers (OR = 1.319), pleuritis (OR = 2.225), malar rash (OR = 1.311), vasculitis (OR = 2.638), serositis (OR = 1.514), hematologic involvement (OR = 1.190), Reynaud’s phenomenon (OR = 1.604), thrombophlebitis (OR = 1.856), and arthritis (OR = 1.256) were positively related to SLE-ON risk. For laboratory features, proteinuria (OR = 1.635), antiphospholipid antibody (OR = 1.450), elevated ESR (OR = 1.623), leukopenia (OR = 1.317), and ACL (OR = 1.657) were positively related to SLE-ON risk as well. Interestingly, the anti-SSA antibody (OR = 0.805) and anti-SSB antibody (OR = 0.764) were negatively related to SLE-ON susceptibility. Usage of cyclophosphamide (OR = 1.869) and steroid pulse therapy (OR = 1.829) was positively correlated with occurrence of SLE-ON. Furthermore, younger age (SMD=−0.175) and the age at onset (SMD=−0.426) significantly related to developing of SLE-ON.

**Conclusion:**

A total of 32 factors including clinical, laboratory features, drug usage, and basic information in SLE patients were related to SLE complicated with osteonecrosis. Of note, the association between cyclophosphamide/steroid pulse therapy and SLE-ON risk may be confounded by underlying disease severity, so these results should be interpreted cautiously. Targeted monitoring and intervention of these modifiable risk factors, especially optimized steroid pulse therapy and cyclophosphamide use and early osteoporosis screening, combined with risk stratification based on anti-SSA/SSB antibody status, may help reduce SLE-ON risk and improve clinical management of SLE patients.

## 1 Introduction

Systemic lupus erythematosus (SLE) is a systemic autoimmune disease characterized by a complex array of clinical manifestations (1). Osteonecrosis (ON) is a prevalent and severe complication in patients with SLE. The main pathological changes of ON include interruption of bone blood flow, bone ischemia, cellular necrosis, and eventual trabecular fractures and collapse of the femoral head (2, 3). As the disease progresses, patients may experience pain in the affected hip joints and limited mobility, which may ultimately require hip replacement (4, 5). This is recognized as a major cause of disability and poor prognosis in SLE patients (6). In addition to common sites such as the femoral head and knee, a recent case report has also described rare peripheral manifestations of osteonecrosis in SLE patients, such as Freiberg’s infraction, further expanding the clinical spectrum of this complication (7). The prevalence of ON in SLE patients (SLE-ON) ranges from 3% to 44% (8), which imposes a significant burden on patients’ families and society. Therefore, comprehensively identifying the potential factors related to SLE-ON is urgent, and early management of these factors is important for patients with SLE-ON.

The etiology and pathogenesis of SLE-ON remain incompletely understood. The development of SLE-ON has been linked to a variety of factors, including high-dose glucocorticoid (GC) use (9), coagulation abnormalities (10), dyslipidemia (11), vasculitis (8), and antiphospholipid antibody dysfunction (12). However, current studies on the factors for SLE-ON risk are heterogeneous and inconsistent. The heterogeneity may stem from differences in sample sizes, study design, diagnostic criteria, and statistical methods, which can lead to conflicting results in risk factor assessment. For example, studies by Kallas et al. (8) and Kwon et al. (13) found a positive correlation between mycophenolate mofetil (MMF) use and an elevated risk of SLE-ON. Conversely, the study by Shaharir et al. (14) suggested that MMF administration was associated with a reduced risk of SLE-ON. Furthermore, Kallas et al. (8) reported no significant association between steroid pulse therapy and the incidence of SLE-ON, whereas Francisco et al. (15) found that such therapy was linked to an increased risk. To date, whether age at disease onset is a risk factor for SLE-ON remains ambiguous, largely because most studies are limited to adult-onset SLE and some lack a multicenter design. Therefore, it is of great importance to conduct a systematic review and comprehensive analysis of potential factors related to SLE-ON.

In this study, we performed a systematic review and meta-analysis by searching for relevant studies. Moreover, three databases established by our research group were additionally included. Finally, a total of 64 studies were used to assess the risk factors for SLE-ON. This will assist clinicians in more accurately identifying high-risk patients in the future and enable the implementation of targeted preventive measures to reduce the incidence of ON and enhance the prognosis of SLE patients.

## 2 Methods

### 2.1 Search strategy

A comprehensive literature search was conducted in multiple databases, including PubMed, Ovid Medline, Web of Science, Embase, Cochrane Library, CNKI, Wanfang Data, and VIP Information. The search time frame spanned from the inception of each database to October 2025. The search strategy combined both Medical Subject Headings (MeSH) terms, such as “systemic lupus erythematosus” and “osteonecrosis,” and free terms including “avascular necrosis,” “ischemic necrosis,” “bone necrosis,” and “aseptic necrosis”. After reviewing the titles and abstracts, full-text articles were evaluated for eligibility. EndNote X9 software was used to identify and remove duplicate records. Studies meeting the following criteria were potentially included: (1) clear definition of the diagnostic criteria for SLE patients; (2) diagnosis of ON using appropriate imaging or clinical techniques; (3) availability of original data on variables including demographic characteristics, clinical features, laboratory indicators, and drug use for both the SLE-ON and SLE-non-ON groups; (4) publication in either English or Chinese. Studies were excluded if any of the following applied: (1) inability to extract valid data from the publication; (2) publication type was a review article, case reports, or conference abstract. Furthermore, when multiple studies were derived from the same study population, only the one with the largest sample size was included. The results in our meta-analysis are summarized in accordance with Meta-Analysis of Observational Studies in Epidemiology (16) and Preferred Reporting Items for Systematic Review and Meta-Analysis (PRISMA) (17). This study was approved by the Ethics Committees of the Affiliated Hospital of Southwest Medical University and Minda Hospital of Hubei Minzu University and was in accordance with the Declaration of Helsinki. Clinical Trial Number was not applicable.

### 2.2 Data extraction

Two researchers independently selected eligible studies and extracted data according to the aforementioned criteria. The extracted data included the following items: first author, year of publication, country, sample size, basic information of patients (e.g., age at SLE diagnosis, age at disease onset, disease duration, and SLE disease activity index (SLEDAI) score), clinical features (e.g., metabolic abnormalities, multi-system involvement, vascular-related manifestations, markers of inflammatory activity, Cushingoid, and osteoporosis), laboratory features (e.g., proteinuria, antiphospholipid antibody, elevated erythrocytes sedimentation rate (ESR), leukopenia, anticardiolipin antibodies (ACL), anti-Sjögren syndrome A antibody (anti-SSA), and anti-Sjögren syndrome B antibody (anti-SSB)), and drug use (e.g., steroid pulse therapy, usage of immunosuppressants). In the event of data discrepancies, the two researchers discussed the issues to resolve them. If no consensus was reached, a third researcher was consulted to determine whether the data should be included in the analysis.

### 2.3 Quality assessment

All studies included in this meta-analysis were case–control studies. The quality of each study was assessed using the Newcastle–Ottawa scale (NOS). The NOS evaluates studies across three dimensions: the selection of study subjects, the comparability between groups, and the assessment of exposure factors. The scale comprises eight items, with a maximum score of nine points. A higher score indicates better methodological quality. Studies with a score of less than five points were excluded.

### 2.4 Rationale for variable selection

The variables included in this meta-analysis were identified based on a comprehensive review of the existing literature regarding factors related to SLE-ON. These factors were selected because they have been consistently evaluated as potential predictors of ON development in patients with SLE in previous epidemiological studies and meta-analysis. To enhance the interpretability of our findings, we have also summarized the specific definitions of key outcomes and exposures (including osteonecrosis diagnosis, neuropsychiatric lupus, serositis, and steroid pulse therapy, among others) across these studies (**Supplementary Table 1**).

### 2.5 Statistical analysis

Raw data were extracted from the included studies, and all statistical analysis was performed using R-studio (version 3.1.1) software. Forest plots were generated to synthesize and visualize the association between potential factors and SLE-ON. The effect size of each factor was analyzed using either a random-effects or the fixed-effects model, with statistical significance defined as P<0.05. For dichotomous variables, pooled odds ratio (OR) and 95% confidence interval (CI) were calculated to estimate between-group differences. For continuous variables, pooled standardized mean difference (SMD) and 95% CI were computed. Between-study heterogeneity was quantified using the I^2^ statistic (range: 0%-100%), where higher values indicate greater heterogeneity. Heterogeneity was also assessed using the P value. When P>0.1 and I^2^<50%, heterogeneity was considered statistically non-significant, and a fixed-effects model was adopted. When P ≤ 0.1 or I^2^≥50%, heterogeneity was considered statistically significant, and a random-effects model was used. A leave-one-out method was employed for sensitivity analysis of the meta-analysis results. Publication bias was identified as a potential factor affecting the reliability of the analysis outcomes. Egger’s test was used to detect publication bias, with P<0.05 considered indicative of significant publication bias.

### 2.6 Patients and public involvement in this study

The patients and public are not in design, conduction, and reporting of the current study.

## 3 Results

### 3.1 Literature search

**Figure 1:**
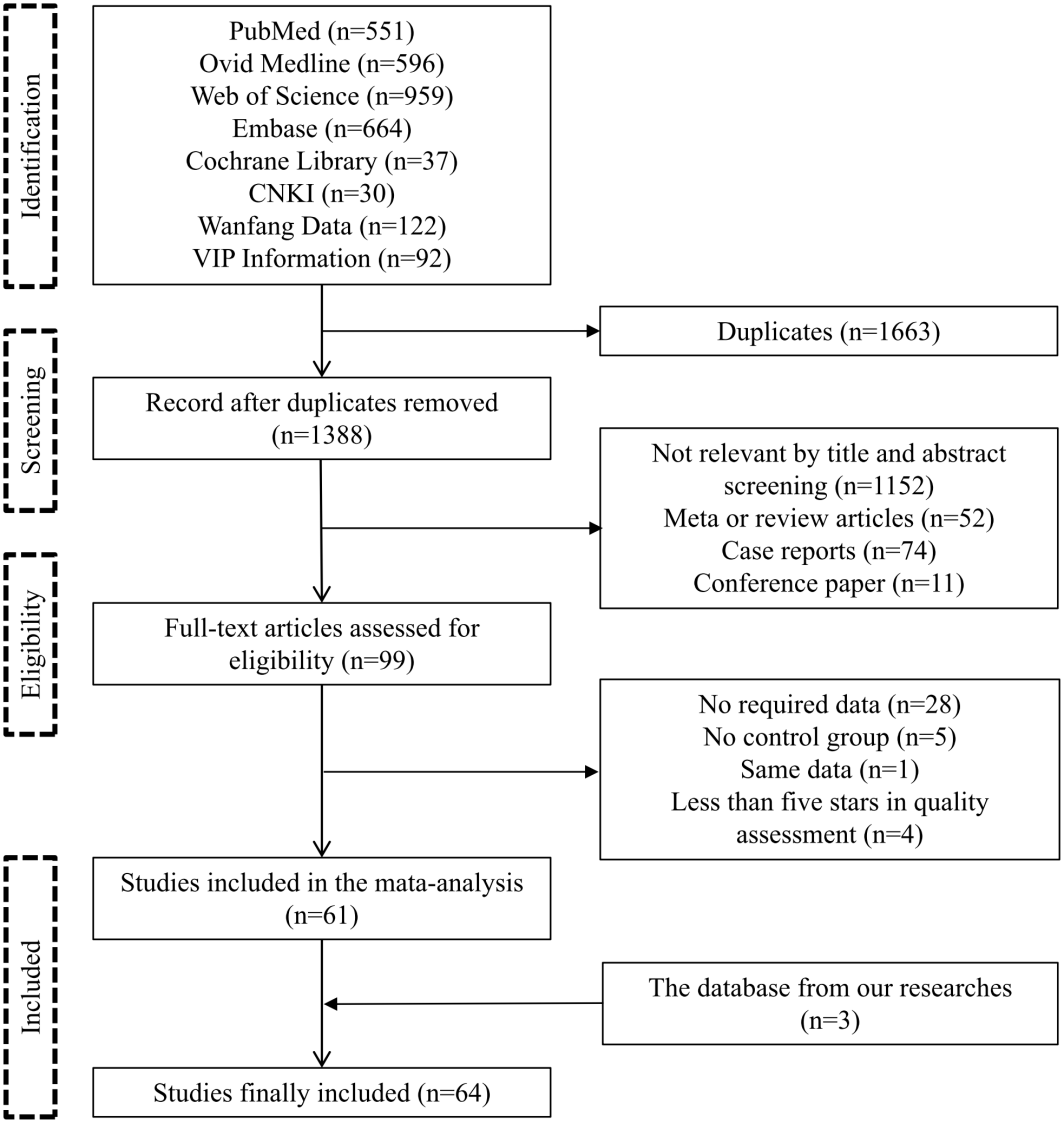
Screening process of studies in the meta-analysis. This figure outlines the systematic literature screening steps for the meta-analysis. Arrows illustrate the sequential flow of the screening stages, whereas side branches detail the number of excluded studies and their respective reasons at each step.

The initial search yielded a total of 3,051 records, of which 1,663 duplicates were removed. The titles and abstracts of the remaining 1,388 records were then screened, and 99 records were identified as potentially eligible. The full texts of these 99 records were evaluated using the NOS, resulting in 61 eligible studies. Additionally, three databases collected by our study group (AHSMU, WCHSCU, and MHMU) were also included in the analysis, with details provided in **Supplementary Tables 2**-**4**. Finally, a total of 64 studies were included in this meta-analysis (9, 11–15, 18–72). The detailed screening process is illustrated in **Figure 1**.

### 3.2 Study characteristics

The characteristics of the 64 included studies are summarized in **Table 1**. Geographically, 45 studies were from Asia, 5 from Europe, 10 from North America, 2 from South America, and 2 from Africa. A total of 2,922 cases and 43,950 controls were ultimately enrolled. The following factors associated with SLE-ON risk were analyzed: general characteristics, clinical features, laboratory findings, and drug usage. The NOS scores of the included studies ranged from 5 to 9. One study achieved the maximum score of 9, whereas 8 studies scored 8. The majority of studies received scores of 7 (n=26) and 6 (n=21).

**Table 1:** Characteristics of included studies.

Study	Publication year	Country	Sample size		Survey year	NOS score
			Case	Control		
Abdelkawy et al.	2022	Egypt	55	715	2014.11-2019.08	8
Cheng et al. (19)	2023	China	106	3985	2009.02-2021.01	8
Xiong et al. (20)	2022	China	26	50	2018.01-2019.12	7
Long et al. (12)	2021	China	88	1070	2009-2020	7
Shaharir et al. (14)	2021	Malaysia	55	335	2017.10-2019.04	6
Dogan et al.	2020	Turkey	11	116	–	8
Hisada et al. (21)	2018	Japan	38	50	2000.01-2017.03	6
Tse et al. (22)	2017	China	55	220	1999-2014	8
Jokar et al. (23)	2016	Iran	29	458	–	6
Kuroda et al. (24)	2015	Japan	21	57	–	6
Sheikh et al. (25)	1998	America	15	11	1987-1994	7
Sekiya et al. (26)	2010	Japan	5	12	2004-2007	9
Migliaresi et al. (27)	1994	Italy	7	62	1985-1991	8
Watanabe et al. (28)	1997	Japan	7	106	–	7
Mok et al. (29)	1998	China	38	143	1971-1997	7
Al Saleh et al. (30)	2010	Emirates	11	115	2002.01-2008.01	7
Massardo et al. (31)	1992	Chile	17	173	1962-1990	6
Joo et al. (32)	2015	Korea	319	20319	2006-2010	6
Yang et al. (33)	2015	Canada	37	111	1982.07-2013.06	6
Ono et al. (34)	1992	Japan	9	42	1984-1988	5
Griffiths et al. (35)	1979	London	8	60	–	7
Calvo-Alen et al. (36)	2006	America	32	59	–	7
Nagasawa et al. (37)	2005	Japan	15	30	1994-1997	6
Ghaleb et al. (38)	2011	Egypt	15	85	2008.11-2010.01	6
Hamijoyo et al. (39)	2008	Philippines	43	93	1995-2005	7
Oinuma et al. (40)	2001	Japan	32	40	–	5
Nagasawa et al. (41)	1989	Japan	24	87	1980-1987	6
Weiner et al. (42)	1989	America	12	15	1975-1987	6
Lee et al. (43)	2014	Korea	64	64	1990.01-2012.04	7
Faezi et al. (44)	2015	Iran	105	560	1979-2009	6
Fialho et al. (45)	2007	Brazil	10	36	2004-2005	5
Sayarlioglu et al. (46)	2012	Turkey	49	154	–	5
Prasad et al. (47)	2007	Canada	65	65	1970-2004	7
Zizic et al. (48)	1985	America	28	26	–	6
Uea-areewongsa et al. (49)	2009	Thailand	20	20	1992-2008	7
Gladman et al. (50)	2001	Canada	70	70	1970-1995	7
Kunyakham et al. (51)	2012	Thailand	65	671	1995.01-2005.08	7
Mont et al. (52)	1997	America	31	72	1995.06-1995.09	6
Smith et al. (53)	1976	America	7	7	1973.01-1974.12	7
Liu et al. (54)	2022	China	26	65	2019.05-2021.06	8
Li et al. (55)	2008	China	32	32	1996-2007	7
Qi et al. (56)	2010	China	27	349	2002.01-2010.01	7
Xuan et al. (57)	2011	China	37	74	–	5
Shen et al. (58)	2012	China	10	30	2009.01-2010.06	7
Shi et al. (59)	2013	China	42	42	2018.01-2012.06	5
Wu et al. (60)	2014	China	16	48	2000.01-2013.03	7
Lin et al. (61)	2014	China	26	26	2010.01-2013.12	7
Wang et al. (62)	2018	China	36	50	1998.01-2015.01	6
Li et al. (63)	2021	China	118	118	2014.01-2019.05	7
Lei et al. (64)	2024	China	65	65	2013.01-2022.12	7
Zhang et al. (65)	2008	China	37	40	2002-2006	6
Liu et al. (66)	2011	China	40	40	2003.10-2008.10	8
Li et al. (67)	2014	China	40	58	2005.01-2013.01	6
Tang et al. (68)	1999	China	29	40	–	5
Shen et al. (69)	2005	China	28	234	–	5
Vílchez-Oya et al. (15)	2019	Spain	6	12	–	6
Gladman et al. (70)	2018	Canada	162	162	–	8
Kwon et al. (13)	2018	Korea	113	764	1998-2014	6
Xu et al. (9)	2024	China	87	706	2013.01-2022.12	6
Chen et al. (71)	2021	China	41	408	2016.01-2019.12	7
Wang et al. (72)	2009	China	32	64	1998-2008	6
AHSMU	2023	China	82	4645	2016.01-2023.09	7
WCHSCU	2020	China	53	2242	2015-2020	7
MHMU	2023	China	93	3272	2019-2023	7

NOS, Newcastle–Ottawa scale; AHSMU, Affiliated Hospital of Southwest Medical University; WCHSCU, West China Hospital of Sichuan University; MHMU, Minda Hospital of Hubei Minzu University.

### 3.3 Factors related to clinical manifestations

**Figure 2:**
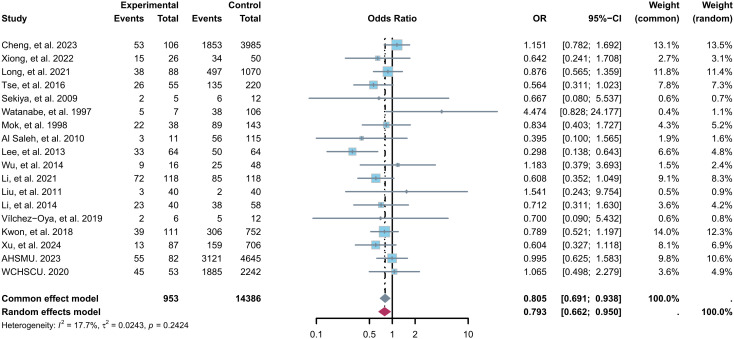
Forest plot of hypertension associated with SLE-ON. The forest plot illustrates the combined effects of hypertension on the development of SLE-ON across 29 studies. The box in the middle of the forest plot represents the study-specific effect size for each study, and the horizontal line segments represent the 95% confidence intervals for the corresponding effect size. The box and line segments at the bottom represent the overall effect size and their confidence intervals for the meta-analysis under both the common effects model and the random effects model. SLE, systemic lupus erythematosus; ON, osteonecrosis; OR, odd ratio; CI, confidence interval.

A comparison of the clinical manifestations between SLE-ON and SLE-non-ON patients is presented in **Table 2**. No significant differences were observed between the two groups in the following characteristics: epilepsy, alopecia, discoid lupus, photosensitivity, pericarditis, anemia, cataract, pulmonary arterial hypertension, antiphospholipid syndrome, and livedo reticularis. A total of 29 studies analyzed the differences in hypertension between SLE-ON patients and controls. Among them, four original studies reported a higher incidence of hypertension in the SLE-ON group, whereas the remaining 25 studies showed no significant difference. The pooled OR was 1.353 (95%CI=1.107-1.655, P = 0.0032) (**Figure 2**). A total of 26 studies analyzed the correlation between neuropsychiatric lupus and SLE-ON risk, with a pooled OR of 1.652 (95%CI=1.391-1.963, P<0.0001) (**Supplementary Figure 1**). Seven studies analyzed the correlation between hyperlipidemia and SLE-ON risk, with a pooled OR of 1.358 (95%CI=1.075-1.717, P = 0.0103) (**Supplementary Figure 2**). A total of 23 studies analyzed the correlation between diabetes mellitus and SLE-ON susceptibility, with a pooled OR of 1.270 (95%CI=1.045-1.543, P = 0.0161) (**Supplementary Figure 3**). There were 31 studies that analyzed the correlation between oral ulcers and SLE-ON risk, with a pooled OR of 1.319 (95%CI=1.023-1.700, P = 0.0329) (**Supplementary Figure 4**). Six studies analyzed the correlation between pleuritis and SLE-ON susceptibility, with a pooled OR of 2.225 (95%CI=1.345-3.679, P = 0.0018) (**Supplementary Figure 5**). There were 31 studies that analyzed the relationship between malar rash and SLE-ON risk, with a pooled OR of 1.311 (95%CI=1.072-1.604, P = 0.0083) (**Supplementary Figure 6**). A total of 32 studies analyzed the relationship between vasculitis and SLE-ON susceptibility, with a pooled OR of 2.638 (95%CI=2.034-3.420, P<0.0001) (**Supplementary Figure 7**). There were 18 studies that analyzed the correlation between serositis and SLE-ON risk, with a pooled OR of 1.514 (95%CI=1.266-1.810, P<0.0001) (**Supplementary Figure 8**). There were 17 studies that analyzed the correlation between nephritis and SLE-ON risk, with a pooled OR of 1.528 (95%CI=1.202-1.942, P = 0.0005) (**Supplementary Figure 9**). A total of 28 studies analyzed the association of renal involvement with SLE-ON risk, with a pooled OR of 1.408 (95%CI=1.147-1.729, P = 0.0011) (**Supplementary Figure 10**). Five studies analyzed the relationship between gastrointestinal involvement and SLE-ON risk, with a pooled OR of 1.998 (95%CI=1.457-2.741, P<0.0001) (**Supplementary Figure 11**). There were 12 studies that explored the relation between CNS involvement and SLE-ON susceptibility, with a pooled OR of 2.010 (95%CI=1.447-2.792, P<0.0001) (**Supplementary Figure 12**). Six studies discussed the correlation between musculoskeletal manifestations and SLE-ON susceptibility, with a pooled OR of 2.002 (95%CI=1.110-3.613, P = 0.0212) (**Supplementary Figure 13**). There were 19 studies that evaluated the correlation between hematologic involvement and SLE-ON risk, with a pooled OR of 1.190 (95%CI=1.021-1.388, P = 0.0261) (**Supplementary Figure 14**). A total of 45 studies determined the association of Reynaud’s phenomenon with SLE-ON susceptibility, with a pooled OR of 1.604 (95%CI=1.302-1.977, P<0.0001) (**Supplementary Figure 15**). Furthermore, 15 studies discussed the correlation between Cushingoid features and SLE-ON risk, with a pooled OR of 3.593 (95%CI=2.347-5.502, P<0.0001) (**Supplementary Figure 16**). There were 16 studies that evaluated the correlation between osteoporosis and SLE-ON susceptibility, with a pooled OR of 2.403 (95%CI=1.405-4.110, P = 0.0014) (**Supplementary Figure 17**). Five studies discussed the correlation between thrombophlebitis and SLE-ON risk, with a pooled OR of 1.856 (95%CI=1.012-3.406, P = 0.0457) (**Supplementary Figure 18**). A total of 38 studies determined the correlation between arthritis and SLE-ON risk, with a pooled OR of 1.256 (95%CI=1.109-1.423, P = 0.0003) (**Supplementary Figure 19**).

**Table 2:** Comparison of clinical manifestations in patients with SLE and SLE-ON.

Clinical manifestation	No. of study	Association of ON in patients with SLE		Heterogeneity		Egger’s test (P value)
		OR (95% CI)	P value	I^2^, %	P value	
Epilepsy	2	0.984 (0.177, 5.458)	0.9854	0.0	0.8167	–
Arthritis^a^	38	1.256 (1.109, 1.423)	0.0003	12.8	0.2506	0.3916
Alopecia	17	1.131 (0.925, 1.383)	0.2317	5.9	0.3859	0.0256
Neuropsychiatric lupus	26	1.652 (1.391, 1.963)	<0.0001	0.0	0.7236	0.6110
Hyperlipidemia	7	1.358 (1.075, 1.717)	0.0103	10.3	0.3505	0.4494
Hypertension^a^	29	1.353 (1.107, 1.655)	0.0032	44.9	0.0053	0.3618
Diabetes mellitus	23	1.270 (1.045, 1.543)	0.0161	20.3	0.1897	0.8757
Oral ulcers^a^	31	1.319 (1.023, 1.700)	0.0329	58.0	<0.0001	0.0950
Pleuritis	6	2.225 (1.345, 3.679)	0.0018	0.0	0.7137	0.3755
Malar rash^a^	31	1.311 (1.072, 1.604)	0.0083	46.6	0.0026	0.2433
Discoid lupus^a^	14	1.300 (0.785, 2.152)	0.3085	40.6	0.0635	0.5787
Photosensitivity	15	1.154 (0.925, 1.439)	0.2037	0.5	0.4440	0.4857
Vasculitis^a^	32	2.638 (2.034, 3.420)	<0.0001	53.1	0.0002	0.0871
Serositis	18	1.514 (1.266, 1.810)	<0.0001	0.0	0.5793	0.2454
Pericarditis	8	0.982 (0.617, 1.564)	0.9387	19.8	0.2724	0.8162
Nephritis^a^	17	1.528 (1.202, 1.942)	0.0005	52.8	0.0056	0.3401
Anemia	18	0.960 (0.775, 1.188)	0.7050	23.5	0.1764	0.1635
Cataract	5	1.191 (0.592, 2.397)	0.6247	42.6	0.1376	0.5232
Renal involvement^a^	28	1.408 (1.147, 1.729)	0.0011	46.9	0.0036	0.8977
Gastrointestinal involvement	5	1.998 (1.457, 2.741)	<0.0001	0.0	0.8179	0.4760
CNS involvement	12	2.010 (1.447, 2.792)	<0.0001	16.2	0.2852	0.1752
Pulmonary arterial hypertension^a^	7	1.152 (0.524, 2.532)	0.7244	69.9	0.0028	0.0967
Musculoskeletal manifestations	6	2.002 (1.110, 3.613)	0.0212	28.7	0.2197	0.0739
Hematologic involvement	19	1.190 (1.021, 1.388)	0.0261	21.3	0.1953	0.6904
Reynaud’s phenomenon^a^	45	1.604 (1.302, 1.977)	<0.0001	49.8	0.0001	0.0064
Cushingoid^a^	15	3.593 (2.347, 5.502)	<0.0001	44.5	0.0323	0.1723
Osteoporosis^a^	16	2.403 (1.405, 4.110)	0.0014	81.8	<0.0001	0.4490
Antiphospholipid syndrome	14	0.971 (0.732, 1.288)	0.8377	0.0	0.9349	0.5438
Livedo reticularis	7	1.222 (0.862, 1.731)	0.2610	0.0	0.5196	0.2188
Thrombophlebitis	5	1.856 (1.012, 3.406)	0.0457	0.0	0.4753	0.2881
Sjögren’s syndrome^a^	4	0.835 (0.271, 2.567)	0.7524	75.0	0.0074	0.5814

^a^ Random-effects model; SLE, systemic lupus erythematosus; ON, osteonecrosis; OR, odds ratio; CI, confidence interval; CNS, central nervous system.

### 3.4 Potential factors correlated with laboratory features

**Figure 3:**
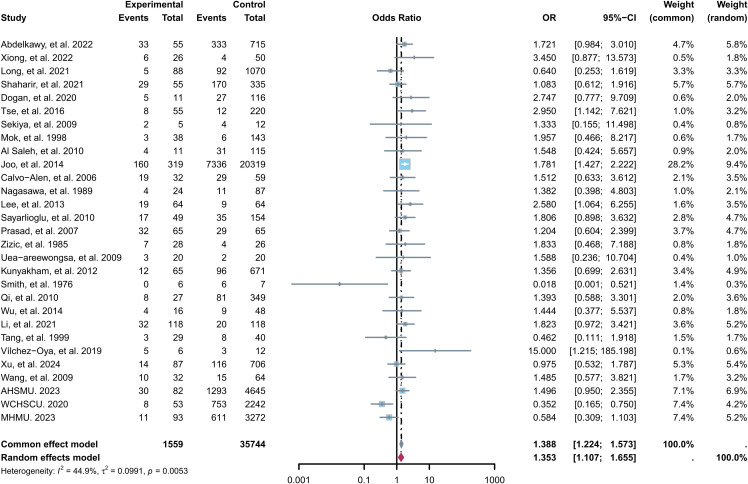
Forest plot of anti-SSA associated with SLE-ON. The forest plot illustrates the combined effects of anti-SSA on the development of SLE-ON across 18 studies. The box in the middle of the forest plot represents the study-specific effect size for each study, and the horizontal line segments represent the 95% confidence intervals for the corresponding effect size. The box and line segments at the bottom represent the overall effect size and their confidence intervals for the meta-analysis under both the common effects model and the random effects model. SLE, systemic lupus erythematosus; ON, osteonecrosis; anti-SSA, anti-Sjogren Syndrome A antibody; OR, odd ratio; CI, confidence interval.

A comparison of the laboratory characteristics between SLE-ON and SLE-non-ON patients is presented in **Table 3**. No significant differences were observed between the two groups in the following characteristics: antinuclear antibody (ANA), hypocomplementemia, anti-double-stranded DNA antibody (anti-dsDNA), anti-smith antibody (anti-Sm), anti-ribonucleoprotein antibody (anti-RNP), aCL IgG, aCL IgM, lupus anticoagulant, thrombocytopenia, and rheumatoid factor (RF). For other factors, 13 studies analyzed the difference in the incidence of proteinuria between the SLE-ON patients and the controls. Among them, two original studies reported a higher incidence of proteinuria in the SLE-ON group, whereas the remaining 11 studies showed no significant difference. The pooled OR was 1.635 (95%CI=1.095-2.441, P = 0.0163) (**Supplementary Figure 20**). There were 18 studies that explored the relationship between anti-SSA and SLE-ON risk, with a pooled OR of 0.805 (95%CI=0.691-0.938, P = 0.0055) (**Figure 3**); 16 studies investigated the relationship between anti-SSB and SLE-ON risk, with a pooled OR of 0.764 (95%CI=0.623-0.936, P = 0.0095) (**Supplementary Figure 21**); and 13 studies examined the relationship between antiphospholipid antibody and SLE-ON susceptibility, with a pooled OR of 1.450 (95%CI=1.008-2.086, P = 0.0449) (**Supplementary Figure 22**). Three studies analyzed the association of elevated ESR and SLE-ON risk, with a pooled OR of 1.623 (95%CI=1.104-2.387, P = 0.0138) (**Supplementary Figure 23**). There were 15 studies that discussed the relationship between leukopenia and SLE-ON susceptibility, with a pooled OR of 1.317 (95%CI=1.055-1.644, P = 0.0151) (**Supplementary Figure 24**). Furthermore, 17 studies analyzed the relationship between ACL and SLE-ON risk, with a pooled OR of 1.657 (95% CI = 1.090-2.519, P = 0.0181) (**Supplementary Figure 25**).

**Table 3:** Comparison of laboratory features in patients with SLE and SLE-ON.

Laboratory features	No. of study	Association of ON in patients with SLE		Heterogeneity		Egger’s test (P value)
		OR (95% CI)	P value	I^2^, %	P value	
Proteinurial^a^	13	1.635 (1.095, 2.441)	0.0163	42.6	0.0516	0.0383
ANA	16	0.932 (0.598, 1.452)	0.7654	0.0	0.8838	0.5516
Anti-SSA	18	0.805 (0.691, 0.938)	0.0055	17.7	0.2424	0.7631
Anti-SSB	16	0.764 (0.623, 0.936)	0.0095	0.0	0.4516	0.6312
Antiphospholipid antibody^a^	13	1.450 (1.008, 2.086)	0.0449	56.5	0.0064	0.0393
Elevated ESR	3	1.623 (1.104, 2.387)	0.0138	0.0	0.6153	0.1297
Leukopenia	15	1.317 (1.055, 1.644)	0.0151	18.8	0.2434	0.9027
Hypocomplementemia^a^	4	0.627 (0.324, 1.213)	0.1657	79.7	0.0020	0.7874
Anti-dsDNA^a^	25	1.213 (0.938, 1.568)	0.1416	62.3	<0.0001	0.7341
Anti-Sm^a^	20	0.874 (0.670, 1.141)	0.3228	58.9	0.0005	0.4407
Anti-RNP^a^	17	1.247 (0.986, 1.577)	0.0652	51.9	0.0068	0.2449
ACL^a^	17	1.657 (1.090, 2.519)	0.0181	58.1	0.0014	0.0207
aCL IgG positive^a^	9	1.255 (0.763, 2.066)	0.3707	50.4	0.0407	0.9135
aCL IgM positive	7	1.378 (0.947, 2.004)	0.0934	0.0	0.7715	0.1410
Lupus anticoagulant	19	0.920 (0.747, 1.135)	0.4373	29.1	0.1203	0.7203
Thrombocytopenia	14	1.060 (0.830, 1.353)	0.6420	22.4	0.2106	0.2335
RF	4	0.919 (0.626, 1.350)	0.6661	0.0	0.7179	0.4119

^a^ Random-effects model; SLE, systemic lupus erythematosus; ON, osteonecrosis; OR, odd ratio; CI, confidence interval; ANA, antinuclear antibody; Anti-SSA, anti-Sjogren Syndrome A antibody; Anti-SSB, anti-Sjogren Syndrome B antibody; ESR, erythrocytes sedimentation rate; Anti-dsDNA, anti-double stranded DNA antibody; Anti-Sm, anti-smith antibody; Anti-RNP, anti-ribonucleoprotein antibody; ACL, anticardiolipin antibodies; RF, rheumatoid factor.

### 3.5 Drug usage in SLE-ON risk

A comparison of the drug use between SLE-ON patients and SLE-non-ON patients is presented in **Supplementary Table 5**. No significant differences were observed between the two groups in the use of the following drugs: azathioprine, MMF, methotrexate, cyclosporine, bisphosphonate, hydroxychloroquine. There were 14 studies that analyzed the difference in cyclophosphamide use between SLE-ON patients and the controls. Among them, six original studies reported higher cyclophosphamide use in the SLE-ON group, whereas the remaining eight studies showed no significant difference. The pooled OR for cyclophosphamide use was 1.869 (95%CI=1.264-2.765, P = 0.0017) (**Supplementary Figure 26**). To assess the impact of disease activity, a sensitivity analysis was performed in nine studies reporting baseline SLEDAI scores. After controlling for disease activity, the association remained significant (OR = 2.328, 95%CI=1.570-3.451, P<0.0001), with a substantial reduction in heterogeneity (I² from 79.1% to 64.6%) (**Supplementary Figure 27**). A total of 28 studies discussed the relationship between steroid pulse therapy and SLE-ON risk: 10 original studies showed a higher frequency of steroid pulse therapy in the SLE-ON group, whereas the remaining 18 studies showed no significant difference between the two groups. The pooled OR for steroid pulse therapy was 1.829 (95%CI=1.460-2.291, P<0.0001) (**Supplementary Figure 28**). The sensitivity analysis restricted to 17 studies reporting SLEDAI scores confirmed the robustness of this association (OR = 1.708, 95%CI=1.276-2.286, P = 0.0003), with stable heterogeneity (I²=41.4%) (**Supplementary Figure 29**). These associations should be interpreted with caution, as they may be confounded by underlying disease severity, although our sensitivity analysis suggests that the relationship remains robust after controlling for disease activity.

### 3.6 Association of general situation with SLE-ON susceptibility

A comparison of the baseline characteristics of SLE-ON and SLE-non-ON patients is presented in **Supplementary Table 6**. No significant difference was observed between the two groups in terms of disease duration. A total of 31 studies analyzed the difference in age between SLE-ON patients and controls. Among them, seven original studies indicated that the SLE-ON group was younger, whereas two studies found the SLE-ON group to be older. The remaining 22 studies showed no significant difference in age between the two groups. The meta-analysis of age revealed a pooled SMD of −0.175 (95%CI=−0.323 to −0.027), P = 0.0203) (**Supplementary Figure 30**). Moreover, 21 studies analyzed the difference in age at onset between the SLE-ON patients and controls, with a pooled SMD of −0.426 (95%CI=−0.842 to −0.009), P = 0.0452) (**Supplementary Figure 31**). A total of 25 studies discussed the difference in SLEDAI between SLE-ON patients and controls, with a pooled SMD of 0.966 (95%CI=0.020-1.913), P = 0.0454) (**Supplementary Figure 32**).

### 3.7 Subgroup analysis by geographic region: association of variables with SLE-ON susceptibility

The results of the subgroup analysis stratified by geographic region are presented in **Table 4**. For osteoporosis, a total of 14 studies were included in the Asian region subgroup, with a pooled OR of 2.573 (95%CI=1.548-4.279, P = 0.0003) (**Supplementary Figure 33**); 2 studies were included in the other regions subgroup, with a pooled OR of 1.617 (95%CI=0.025-105.461, P = 0.8215) (**Supplementary Figure 34**). For hydroxychloroquine, a total of 18 studies were included in the Asian region subgroup, with a pooled OR of 0.665 (95%CI=0.440-1.004, P = 0.0520) (**Supplementary Figure 35**); 6 studies were included in the other regions subgroup, with a pooled OR of 1.149 (95%CI=0.861-1.533, P = 0.3464) (**Supplementary Figure 36**). For mycophenolate mofetil, a total of 10 studies were included in the Asian region subgroup, with a pooled OR of 1.424 (95%CI=0.803-2.528, P = 0.2267) (**Supplementary Figure 37**); one study was included in the other regions subgroup, with an OR of 1.283 (95%CI=0.560-2.939, P = 0.5564) (**Supplementary Figure 38**). For age at onset, a total of 14 studies were included in the Asian region subgroup, with a pooled SMD of −0.620 (P = 0.0513) (**Supplementary Figure 39**); 7 studies were included in the other regions subgroup, with a pooled SMD of −0.102 (P = 0.2324) (**Supplementary Figure 40**). For disease duration, a total of 16 studies were included in the Asian region subgroup, with a pooled SMD of 0.862 (P = 0.1073) (**Supplementary Figure 41**); 6 studies were included in other regions subgroup, with a pooled SMD of 0.066 (P = 0.6928) (**Supplementary Figure 42**). For SLEDAI, a total of 18 studies were included in the Asian region subgroup, with a pooled SMD of 1.351 (P = 0.0385) (**Supplementary Figure 43**); 7 studies were included in other regions subgroup, with a pooled SMD of 0.035 (P = 0.6490) (**Supplementary Figure 44**).

**Table 4:** Subgroup analysis by region: association of variables with the risk of SLE-ON.

Variable	Asian region			Other regions		
	OR/SMD (95% CI)	P value	I^2^, %	OR/SMD (95% CI)	P value	I^2^, %
Osteoporosis	2.573 (1.548, 4.279)	0.0003	82.2	1.617 (0.025, 105.461)	0.8215	84.8
Hydroxychloroquine	0.665 (0.440, 1.004)	0.0520	84.4	1.149 (0.861, 1.533)	0.3464	13.3
Mycophenolate mofetil	1.424 (0.803, 2.528)	0.2267	85.2	1.283 (0.560, 2.939)	0.5564	–
Age at onset	−0.620 (−1.244, 0.004)	0.0513	94.3	−0.102 (−0.269, 0.065)	0.2324	38.7
Disease duration	0.862 (−0.187, 1.911)	0.1073	97.7	0.066 (−0.261, 0.393)	0.6928	72.2
SLEDAI	1.351 (0.072, 2.631)	0.0385	99.0	0.035 (−0.117, 0.188)	0.6490	0.0

NOS, Newcastle–Ottawa scale; SLE, systemic lupus erythematosus; ON, osteonecrosis; OR, odd ratio; SMD, standardized mean difference; CI, confidence interval; SLEDAI, SLE disease activity index.

### 3.8 Subgroup analysis by NOS score: association of variables with SLE-ON susceptibility

The results of the subgroup analysis stratified by NOS score are presented in **Table 5**. For osteoporosis, a total of 11 studies were included in the NOS score ≥7 subgroup, with a pooled OR of 2.824 (95%CI=1.300-6.132, P = 0.0087) (**Supplementary Figure 45**); 5 studies were included in the NOS score <7 subgroup, with a pooled OR of 1.860 (95%CI=1.006-3.436, P = 0.0477) (**Supplementary Figure 46**). For hydroxychloroquine, a total of 16 studies were included in the NOS score ≥7 subgroup, with a pooled OR of 0.835 (95%CI=0.557-1.251, P = 0.3814) (**Supplementary Figure 47**); 8 studies were included in the NOS score <7 subgroup, with a pooled OR of 0.685 (95%CI=0.384-1.224, P = 0.2019) (**Supplementary Figure 48**). For mycophenolate mofetil, a total of six studies were included in the NOS score ≥7 subgroup, with a pooled OR of 1.285 (95%CI=0.910-1.816, P = 0.1541) (**Supplementary Figure 49**); five studies were included in the NOS score <7 subgroup, with a pooled OR of 1.214 (95%CI=0.447-3.296, P = 0.7041) (**Supplementary Figure 50**). For age at onset, a total of 15 studies were included in the NOS score ≥7 subgroup, with a pooled SMD of −0.598 (P = 0.0429) (**Supplementary Figure 51**); 6 studies were included in the NOS score <7 subgroup, with a pooled SMD of −0.060 (P = 0.5456) (**Supplementary Figure 52**). For disease duration, a total of 15 studies were included in the NOS score ≥7 subgroup, with a pooled SMD of 0.756 (P = 0.1637) (**Supplementary Figure 53**); 7 studies were included in the NOS score <7 subgroup, with a pooled SMD of 0.399 (P = 0.4135) (**Supplementary Figure 54**). For SLEDAI, a total of 16 studies were included in the NOS score ≥7 subgroup, with a pooled SMD of 0.715 (P = 0.0324) (**Supplementary Figure 55**); 9 studies were included in the NOS score <7 subgroup, with a pooled SMD of 1.379 (P = 0.2641) (**Supplementary Figure 56**).

**Table 5:** Subgroup analysis by NOS score: association of variables with the risk of SLE-ON.

Variable	NOS score≥7			NOS score<7		
	OR/SMD (95% CI)	P value	I^2^, %	OR/SMD (95% CI)	P value	I^2^, %
Osteoporosis	2.824 (1.300, 6.132)	0.0087	80.3	1.860 (1.006, 3.436)	0.0477	76.7
Hydroxychloroquine	0.835 (0.557, 1.251)	0.3814	68.6	0.685 (0.384, 1.224)	0.2019	89.1
Mycophenolate mofetil	1.285 (0.910, 1.816)	0.1541	0.0	1.214 (0.447, 3.296)	0.7041	92.4
Age at onset	−0.598 (−1.176, −0.019)	0.0429	93.7	−0.060 (−0.255, 0.135)	0.5456	49.7
Disease duration	0.756 (−0.308, 1.819)	0.1637	95.1	0.399 (−0.557, 1.354)	0.4135	98.5
SLEDAI	0.715 (0.060, 1.371)	0.0324	96.3	1.379 (−1.041, 3.799)	0.2641	99.4

NOS, Newcastle-Ottawa scale; SLE, systemic lupus erythematosus; ON, osteonecrosis; OR, odd ratio; SMD, standardized mean difference; CI, confidence interval; SLEDAI, SLE disease activity index.

### 3.9 Analysis of publication bias and sensitivity analysis

The Egger’s test was used to assess the publication bias of the meta-analysis. The results indicated the presence of publication bias in the meta-analyses of alopecia, Raynaud’s phenomenon, proteinuria, antiphospholipid antibody, ACL, and SLEDAI. For the remaining study factors, no statistically significant publication bias was detected (**Tables 2**, **3**; **Supplementary Tables 5**, **6**). To further evaluate the impact of publication bias, we performed trim−and−fill adjustment for these six indicators with significant publication bias, and adjusted pooled effect sizes are presented in **Table 6**. After correction, alopecia changed from non−significant to statistically significant, Raynaud’s phenomenon and SLEDAI remained significant, whereas proteinuria, antiphospholipid antibody, and ACL lost statistical significance. Notably, no hypothetical unpublished studies were imputed for SLEDAI, indicating its high robustness against publication bias. Additionally, the leave-one-out method confirmed the stability of the meta-analysis results, as excluding any single study did not substantially alter the pooled effect sizes of each factor (**Supplementary Tables 7**-**38**).

**Table 6:** Pooled effect sizes after trim-and-fill adjustment for indicators with significant publication bias.

Variable	Imputed studies	Adjusted OR/SMD (95%CI)	P value
Alopecia	3	1.266 (1.036-1.546)	0.0208
Raynaud’s phenomenon	8	1.306 (1.007-1.693)	0.0439
Proteinuria	5	1.133 (0.721-1.779)	0.5874
Antiphospholipid antibody	5	1.010 (0.672-1.516)	0.9625
ACL	5	1.149 (0.711-1.857)	0.5711
SLEDAI	0	0.966 (0.020-1.913)	0.0454

OR, odd ratio; SMD, standardized mean difference; CI, confidence interval; ACL, anticardiolipin antibodies; SLEDAI, SLE disease activity index.

## 4 Discussion

### 4.1 Rationale and key updates of the meta-analysis

It is widely recognized that glucocorticoid (GC) therapy is beneficial for the management of SLE. However, this treatment is also a well-established contributor to the development of SLE-ON (73), and the onset of SLE-ON is linked to additional risk factors. Notably, a proportion of SLE patients treated with high-dose GC do not develop ON during the disease course (74). Conversely, some patients with ON have never received GC therapy (22). Furthermore, the incidence of ON in SLE patients is higher than in other rheumatic diseases that also require GC treatment (75). These observations suggest that additional factors may contribute to SLE-ON development. To date, studies investigating potential factors for SLE-ON risk have shown considerable heterogeneity. In this study, we performed a comprehensive meta-analysis to explore the potential factors for SLE-ON by integrating relevant literature from all periods, and we incorporated data from three databases. Ultimately, 64 studies were included in this systematic review and meta-analysis. Our results demonstrated that the age at SLE diagnosis, age at disease onset, and SLEDAI score were associated with an increased risk of SLE-ON. Compared with the controls, the SLE-ON group showed a positive association with metabolic abnormalities (hyperlipidemia, hypertension, and diabetes mellitus), multisystem involvements (CNS involvement, neuropsychiatric lupus, serositis, pleuritis, nephritis, renal involvement, gastrointestinal involvement, musculoskeletal manifestations, hematologic involvement, and arthritis), and a higher prevalence of vascular-related manifestations (vasculitis, Raynaud’s phenomenon, and thrombophlebitis), markers of inflammatory activity (oral ulcers, and malar rash), and Cushingoid features and osteoporosis. Regarding laboratory indices, proteinuria, antiphospholipid antibodies, elevated ESR, leukopenia, and anticardiolipin antibodies (ACL) were positively associated with a high risk of SLE-ON, whereas anti-SSA and anti-SSB antibodies were negatively associated with SLE-ON risk. In terms of drug exposure, the use of cyclophosphamide and steroid pulse therapy was closely associated with an increased risk of SLE-ON. A previous meta-analysis published in 2014 (76) discussed factors related to SLE-ON, reporting a high prevalence of arthritis, alopecia, oral ulcers, vasculitis, pleurisy, hypertension, Cushingoid features, and gastrointestinal and renal involvement in SLE-ON patients but found no correlation between laboratory indices and SLE-ON susceptibility. Additionally, that meta-analysis did not fully analyze the association of clinical features, medication use, and general conditions with SLE-ON risk. Furthermore, the limited number of included studies, small sample size, and insufficient data for pooled analysis may have influenced the conclusions. In our meta-analysis, we have made significant updates in several key aspects: The number of included studies increased substantially (from 16 to 64), and the sample size expanded nearly 20-fold (from 2,384 to 46,872 participants), greatly improving the statistical power and reliability of our conclusion. We identified additional factors associated with SLE-ON risk, including diabetes mellitus, skin rash, serositis, CNS involvement, Raynaud’s phenomenon, positive anti-SSA, anti-SSB, antiphospholipid antibodies, and leukopenia. We also newly included factors such as neuropsychiatric lupus, hyperlipidemia, nephritis, musculoskeletal involvement, hematological involvement, osteoporosis, thrombophlebitis, and laboratory indices (proteinuria, elevated ESR, and ACL). It is worth noting that the previous meta-analysis suggested an association between alopecia and SLE-ON. However, in our meta-analysis, which incorporated more studies, alopecia was not significantly associated with SLE-ON risk. This discrepancy may be related to the increased ethnic diversity of the included population and optimized GC administration in more recent studies. Overall, our meta-analysis has comprehensively expanded the spectrum of potential factors for SLE-ON based on diverse databases and a larger, more diverse dataset.

### 4.2 General situation and SLE-ON risk

Compared with the SLE-non-ON group, SLE-ON patients had an earlier mean age at onset and diagnosis. These findings are consistent with previous studies (12, 13, 44). Younger age correlates with higher estrogen expression and a more active gut microbiota compared with older patients (53). In turn, high estrogen levels affect thymosin production, leading to the activation of inflammatory T cells, abnormal differentiation of T helper cells, and excessive autoantibody production. Disease activity is an important risk factor for SLE-ON. A higher SLEDAI score is associated with earlier onset of SLE-ON (18, 77), and the SLEDAI score is positively related to SLE-ON susceptibility. A higher SLEDAI score indicates a more active SLE state, which correlates with more severe organ and system damage, such as in the hematologic system and joints. This may also be related to the use of high-dose glucocorticoids to control disease activity and severity. However, some studies do not support the idea that SLEDAI is an independent risk factor for SLE-ON (9, 76). Therefore, future multicenter studies with large sample sizes and diverse patient groups with varying disease activities are needed to follow up and evaluate the impact of the SLEDAI score on SLE-ON susceptibility.

### 4.3 Clinical manifestations and SLE-ON risk

Hypertension is a severe clinical feature characterized by abnormally high pressure exerted by blood flow on vessel walls. It may lead to stroke, coronary heart disease, and heart failure. In previous study, only one study reported a correlation between hypertension and SLE-ON risk (15). However, our meta-analysis revealed a significant association between hypertension and SLE-ON, and we also found a significant association of hyperlipidemia and diabetes mellitus with SLE-ON, respectively. This discrepancy could be attributed to the limited number of SLE-ON patients in these single-center studies. Furthermore, our findings suggest that hypertension, hyperlipidemia, and diabetes mellitus could serve as novel predictive indicators for SLE-ON development. The physiological effects of insulin are disrupted due to imbalances in PI3K and MAPK signaling. Inhibition of the PI3K pathway leads to reduced endothelial nitric oxide (NO) production, resulting in endothelial dysfunction. If the MAPK pathway is not inhibited, it promotes the production of endothelin-1 in vascular smooth muscle cells. Therefore, diabetes mellitus in SLE patients can lead to vascular abnormalities and promote atherosclerosis, which may cause arterial occlusion. Interestingly, on the one hand, insulin resistance activates the sympathetic nervous system, upregulates the expression of angiotensin II (Ang2), and downregulates NO synthesis, leading to increased blood pressure. On the other hand, high expression of leptin and activation of the hypothalamus–pituitary–adrenal (HPA) axis further promote sympathetic nervous system activation. Finally, elevated intra-articular or intraosseous pressure induces venous drainage obstruction, leading to bone necrosis. Moreover, SLE patients with hyperlipidemia exhibit a high expression of free fatty acids (FFA), which inhibit the anti-lipid effects of insulin. High FFA levels stimulate the liver to produce more low-density lipoprotein (LDL), which is prone to oxidation and uptake into the arterial wall. This causes atherosclerosis and activation of the reactive oxygen species (ROS) system, destroying the integrity of endothelial cells and leading to arterial damage.

The findings of this study demonstrated a significant correlation between SLE-ON and multiple-organ involvements, including CNS involvement, neuropsychiatric lupus, serositis, pleuritis, nephritis and renal system involvement, gastrointestinal involvement, musculoskeletal manifestations, hematologic involvement, and arthritis. This may be attributable to the fact that organ involvement increases the intensity of treatment, which in turn elevates the risk of SLE-ON. The higher likelihood of ON development in patients with CNS involvement or neuropsychiatric lupus may be explained by the life-threatening nature of some clinical manifestations of neuropsychiatric lupus. Consequently, these patients are more likely to receive higher doses of glucocorticoids (GC) to shorten the disease course (12). Moreover, GC use is a well-established major risk factor for ON development in SLE patients. An elevated risk of musculoskeletal involvement was observed in the SLE-ON group compared with the controls. This may be attributable to the substantial increase in statin use in these patients, which has been demonstrated to induce neuromuscular side effects (78) and result in musculoskeletal discomfort. As demonstrated by several studies (12, 13, 22, 79), nephritis and renal involvement have been identified as risk factors for SLE-ON development, which is consistent with our findings. It has been hypothesized that patients with renal injury are prone to dyslipidemia, altered concentrations of coagulation-related proteins, and accelerated atherosclerosis, thereby promoting thrombosis and causing osteonecrosis (49). Another viewpoint posits that SLE frequently results in renal injury (80, 81), and the exacerbation of renal injury is often accompanied by short-term administration of large amounts of GC, which indirectly promotes the development of ON (82). In addition, our study found a higher prevalence of gastrointestinal involvement, serositis, pleuritis, and arthritis in SLE-ON patients, which is consistent with the results of several studies (12, 19, 76). However, conflicting results still exist regarding the association between organ involvement and SLE-ON. SLE patients with gastrointestinal involvement sometimes exhibit metabolic dysfunction of the gut microbiota, particularly a disorder in the degradation and metabolism of heterologous biomass. This leads to a disruption of the unsaturated fatty acid biosynthesis function in the gut microbiota, which further results in dysregulation of host lipid metabolism and contributes to the occurrence of osteonecrosis. Serositis and pleuritis are severe clinical features in SLE patients, and those with these conditions are often treated with high doses of GC, which may increase the risk of SLE-ON development. Imbalance between osteoblasts and osteoclasts can lead to bone invasion and loss, joint inflammation, and arthritis. These damages are related to factors such as diabetes mellitus complications, long-term use of high-dose GC, and anticoagulant use. The blood vessels in the joints of SLE patients with arthritis are easily affected by pro-inflammatory factors, such as the high expression of pro-inflammatory cytokines and chemokines present in arthritic joints. All these factors can affect blood supply and cause osteonecrosis.

The results of this study demonstrated that certain vascular-related manifestations (vasculitis, Raynaud’s phenomenon, and thrombophlebitis) were positively correlated with the risk of SLE-ON. This observation is consistent with the established pathological mechanisms of SLE (10). Immune complexes formed by autoantibodies and self-antigens deposit on the blood vessel walls, activating the complement system and triggering inflammation. This leads to the aggregation of inflammatory cells within the vessel walls, causing damage to blood vessels and tissues, and subsequent disturbances in the coagulation and fibrinolysis systems. Ultimately, this can result in thrombophlebitis, thrombosis, and even vascular occlusion, contributing to the development of osteonecrosis (10). SLE patients with Raynaud’s phenomenon exhibit inflammation and spasm of the blood vessels supplying bone tissue, as well as excessive endothelial cell proliferation and luminal stenosis, which exacerbate ischemia in bone tissues. In addition, the inflammation triggered by SLE contributes to the development of ON. For instance, our study found a positive correlation between oral ulcers, malar rash, and the risk of SLE-ON. Osteoporosis is also a risk factor for SLE-ON.

Osteoporosis can lead to a reduction in the number and strength of bone trabeculae, as well as microcirculatory disorders characterized by reduced microvessel density, decreased vascular permeability, and narrowed vessel lumens. These changes may ultimately contribute to the development of ON (83). Furthermore, studies have found a higher incidence of Cushingoid features in patients with SLE-ON. While the exact mechanism remains unclear, it may be associated with the use of glucocorticoids as a potential side effect.

### 4.4 Laboratory features and SLE-ON risk

Regarding laboratory indicators, our study showed that a positive antiphospholipid antibody (aPL) is positively related to the development of SLE-ON, which is consistent with the results of multiple studies (12, 29, 84). However, among different types of aPLs, we did not observe a relationship between lupus anticoagulant and SLE-ON but found a significant correlation between anticardiolipin antibody (ACL) and SLE-ON. This indicates that different types of aPLs may contribute to SLE-ON pathogenesis to varying degrees, and further research is crucial for clarifying their specific roles. It is noteworthy that our study revealed that the positive rates of anti-SSA and anti-SSB antibodies in the SLE-ON group were both lower than those in the SLE-non-ON group. This exploratory finding should be interpreted with caution, as residual confounding cannot be excluded, and the observed association does not imply causation. We hypothesize that a potential negative feedback mechanism may exist, which inhibits the transformation of B cells into plasma cells, suppresses B-cell activation, and prevents B-cell hyperactivity, leading to a lower expression of anti-SSA and anti-SSB antibodies in SLE-ON patients. However, this hypothesis is speculative and requires further validation. Importantly, this result should be regarded as preliminary, and future prospective studies with larger sample sizes and more comprehensive adjustment for potential confounding factors are needed to confirm these findings and clarify the underlying mechanisms. Furthermore, the results of this study indicated that leukopenia, antiphospholipid antibody positivity, and elevated erythrocyte sedimentation rate are also positively related to SLE-ON. SLE patients with leukopenia, which may manifest as neutropenia or lymphopenia, often exhibit a high expression of anti-lymphocyte antibodies (such as anti-DNA antibody and anti-ribosomal P antibody) and increased lymphocyte apoptosis. These abnormalities lead to excessive autoantibody deposition in vessel walls and subsequent vascular dysfunction. SLE patients with a high expression of antiphospholipid antibodies may develop hypercoagulability and damage to the fibrinolytic system, resulting in a pro-thrombotic state in the blood and promoting thrombus formation. Elevated ESR in SLE patients indicates a more active disease state, which may induce macrophages to activate a large number of pro-inflammatory cells and promote osteoclast formation through various pro-inflammatory mediators and cytokines, ultimately contributing to the occurrence of osteonecrosis.

### 4.5 Drug usage and SLE-ON risk

Some studies have shown that glucocorticoid pulse therapy may increase the risk of SLE-ON (8, 21, 85). For example, Moghazy et al. reported that treatment of SLE patients with glucocorticoids was associated with the pathogenesis of ON, particularly long duration of glucocorticoid use and high cumulative doses, which can result in severe ON (18). However, other studies have suggested that glucocorticoid pulse therapy is not a risk factor for SLE-ON (9, 12). These discrepancies may be attributed to differences in study design, such as varying cumulative glucocorticoid doses and sample sizes. In our meta-analysis, we confirmed that glucocorticoid use was overall associated with an increased risk of SLE-ON. There are also conflicting views regarding the relationship between immunosuppressant use and the risk of SLE-ON. Some studies have reported an association between the use of immunosuppressants and an increased risk of SLE-ON (9, 86). In contrast, other studies have found that hydroxychloroquine use is associated with a reduced risk of developing SLE-ON (12, 87, 88). In our analysis, we found that cyclophosphamide use was associated with an increased risk of SLE-ON, whereas no significant association was observed between the risk of SLE-ON and the use of other immunosuppressants, such as azathioprine and methotrexate.

It should be emphasized that the observed association between cyclophosphamide, glucocorticoid pulse therapy, and SLE-ON risk should be interpreted with caution due to the possibility of confounding by indication. Patients receiving these intensive treatments typically have more severe disease or higher disease activity, which may independently increase the risk of osteonecrosis. To address this limitation, we performed a sensitivity analysis restricted to studies that reported baseline SLEDAI scores. After controlling for disease activity, the significant association of both cyclophosphamide and glucocorticoid pulse therapy with SLE-ON risk remained. Notably, heterogeneity decreased substantially for cyclophosphamide (I² from 79.1% to 64.6%), confirming disease activity as an important source of between-study heterogeneity.

### 4.6 Sources of heterogeneity and subgroup analyses

Several of our pooled analysis exhibited moderate to high between-study heterogeneity. To explore the potential sources of this variability, we performed two prespecified subgroup analysis for variables with high heterogeneity (including osteoporosis, mycophenolate mofetil, hydroxychloroquine, age at onset, disease duration, and SLEDAI), stratified by geographic region (Asian vs. other regions) and study quality (NOS score ≥7 vs. <7).

Subgroup analysis revealed that the positive association of osteoporosis and SLEDAI score with SLE-ON risk was statistically significant only in the Asian population, whereas no significant association was observed in other regions. This discrepancy may be attributable to racial/ethnic differences in genetic predisposition, lifestyle factors (e.g., lower vitamin D levels and reduced sun exposure in Asian populations), and regional variations in SLE management strategies, including glucocorticoid dosing and bone protection protocols. These differences likely contributed substantially to the between-study heterogeneity observed in the overall analysis.

Stratification by study quality showed that the association of age at onset and SLEDAI with SLE-ON risk was more pronounced and statistically robust in high-quality studies (NOS ≥7). In contrast, these association was weaker or non-significant in lower-quality studies (NOS <7). This suggests that publication bias, selection bias, or residual confounding in lower-quality studies may have inflated between-study heterogeneity. Our findings highlight the importance of considering study quality when interpreting the results of meta-analysis in this field.

Despite these subgroup analyses providing valuable insights into the sources of heterogeneity, we were unable to perform additional stratification based on age at SLE onset (adult vs. childhood), imaging modalities for ON diagnosis, or definitions of cumulative glucocorticoid dose, due to the limited availability of detailed data in the included primary studies. In addition, high residual heterogeneity persisted within several subgroups even after stratification by geographic region and study quality. This may be related to inconsistent diagnostic criteria for osteonecrosis, variable definitions of risk factors, and differences in glucocorticoid dose regimens, which may partially account for such residual heterogeneity. These limitations should be carefully considered when generalizing our findings.

### 4.7 Publication bias and sensitivity analysis

The Egger’s test detected publication bias in the analysis of alopecia, Raynaud’s phenomenon, proteinuria, antiphospholipid antibody, ACL, and SLEDAI in the meta-analysis. This may lead to over- or underestimation of the pooled effect sizes for these factors, as studies with positive or statistically significant results are more likely to be published, whereas those with null or non-significant findings are often unreported. After trim−and−fill adjustment, alopecia turned from non−significant to significant, indicating that its true risk effect was underestimated by publication bias. Raynaud’s phenomenon and SLEDAI remained stable risk factors unaffected by publication bias, whereas proteinuria, antiphospholipid antibody, and ACL lost statistical significance, suggesting that their initial positive association was spurious and driven mainly by publication bias. However, the leave-one-out sensitivity analysis showed that the overall results remained stable after excluding any single study, suggesting that the main conclusions of this meta-analysis are robust despite the presence of publication bias for some indicators.

## 5 Limitation

Since we have discussed many potential factors that may correlate with SLE-ON risk, this study has some limitations. First, all the studies included are observational studies. Therefore, the causal relationship of the factors and SLE-ON susceptibility needs experimental studies to confirm. Second, some studies have relatively small sample sizes. Selection of the population in the study may have suffered from selection bias. Third, despite our systematic summary of the operational definitions for key variables, inherent heterogeneity in the operational definitions of several variables across the included studies persists. Coupled with inconsistent standards for data processing and confounding factor adjustment in the original studies, this heterogeneity may have compromised the overall methodological quality of the pooled evidence. Additionally, some of the analyzed variables are clinically overlapping (e.g., neuropsychiatric lupus includes CNS lupus, and serositis includes pleuritis). However, due to the lack of detailed subclassification data in the included studies, we were only able to analyze these variables independently, in line with previous literature. This may limit the interpretability of the results, and future studies with more granular data should consider both individual and combined analysis of these overlapping manifestations. Moreover, publication bias existed in some factors in the current meta-analysis. Thus, conclusion regarding these factors should be interpreted with caution. Lastly, we only analyzed steroid pulse therapy as a binary variable in this meta-analysis, since most included primary studies did not report detailed data on glucocorticoid cumulative dose, treatment duration, or daily dose. These important dose-related confounders could not be further assessed, which represents a major limitation of the present study.

## 6 Conclusion

This study comprehensively discussed potential factors related to SLE-ON development based on available databases up to date. We found many clinical and laboratory features, treatment usage, and general situation either positively correlating with SLE-ON risk or negatively correlating with SLE-ON susceptibility. In the future, regarding diagnosis and treatment of SLE-ON patients, we suggest the physicians pay more attention to these factors and select precise interventions for prevention to treat SLE-ON patients as soon as possible.

## Data Availability

The original contributions presented in the study are included in the article/**Supplementary Material**. Further inquiries can be directed to the corresponding authors.
