# Supplementary material for: Meta-analysis of factors for osteonecrosis in systemic lupus erythematosus: integration of comprehensive literatures and multicenter databases
Source: Front Immunol. 2026 Jul 2;17:1679237. doi: 10.3389/fimmu.2026.1679237 (PMC13372907; doi:10.3389/fimmu.2026.1679237)
Supplement: Supplementary file 1 [file DataSheet1.zip › Supplementary Material/Supplementary table 1.docx]

#### **Supplementary table 1 Operational definitions of key variables in this meta-analysis.**

| Variable | Operational definitions in This Meta-Analysis |
| --- | --- |
| Osteonecrosis | Osteonecrosis is defined as a condition confirmed by at least one imaging modality (X-ray, MRI, CT, or bone scan). |
| Steroid pulse therapy | Steroid pulse therapy is defined as the intravenous administration of corticosteroids at a daily dose of ≥250 mg prednisone equivalent for at least 1 day. |
| SLEDAI | SLEDAI is used to assess and define the disease activity status of patients with SLE. |
| Neuropsychiatric lupus | Neuropsychiatric lupus is defined as one or more neuropsychiatric syndromes defined by the American College of Rheumatology occurring in patients with SLE, after excluding other identifiable etiologies. |
| Serositis | Serositis refers to inflammation of the serous membranes, whose clinical manifestations are mainly pleurisy, pericarditis, or peritonitis. |
| Arthritis | Arthritis can be diagnosed when the clinical manifestations are joint pain, swelling and limited mobility, combined with imaging examinations (such as X-ray and ultrasound) showing joint inflammatory changes, after excluding similar diseases such as traumatic joint injury and gout. |
| Discoid lupus | Discoid lupus can be diagnosed when the clinical manifestation is characteristic discoid erythema (round or oval, well-demarcated, with adherent scales), combined with skin histopathological examination showing hyperkeratosis, follicular plugging, and liquefaction degeneration of basal cells, after excluding other similar skin diseases (such as psoriasis and seborrheic dermatitis). |
| Photosensitivity | Photosensitivity can be diagnosed when the clinical manifestation is the rapid appearance of erythema, pruritus and burning sensation on the skin after exposure to sunlight (or ultraviolet rays), accompanied by blisters and erosion in severe cases, after excluding similar diseases such as sunburn and contact dermatitis. |
| Vasculitis | Vasculitis can be diagnosed when the clinical manifestations are symptoms related to vascular involvement (such as skin ecchymoses, joint pain, fatigue), combined with vascular imaging examinations (such as vascular ultrasound, CTA) or histopathological examination showing vascular wall inflammation and necrosis, after excluding similar diseases such as infectious vascular lesions and thrombosis. |
| Nephritis | Nephritis is inflammation of the kidneys, typically presenting with hematuria, proteinuria, and possibly accompanied by edema. |
| Anemia | Anemia can be diagnosed when the clinical manifestations are symptoms such as fatigue, dizziness, pale complexion and palpitations, combined with blood routine examination showing that the hemoglobin level is lower than the normal reference value, after excluding similar etiologies such as blood loss, hematopoietic dysfunction and hemolytic diseases. |
| Renal involvement | Renal involvement can be diagnosed when the clinical manifestations are kidney-related symptoms such as proteinuria, hematuria, edema and hypertension, combined with routine urine, renal function and renal imaging examinations showing renal abnormalities, after excluding similar diseases such as primary kidney diseases and infectious nephropathy. |
| Gastrointestinal involvement | Gastrointestinal involvement can be diagnosed when the clinical manifestations are gastrointestinal-related symptoms such as abdominal pain, diarrhea, nausea, vomiting and gastrointestinal bleeding, combined with gastroenteroscopy and imaging examinations showing abnormalities or lesions of the gastrointestinal mucosa, after excluding similar diseases such as primary gastrointestinal diseases and infectious gastroenteropathy. |
| CNS involvement | CNS involvement can be diagnosed when the clinical manifestations are central nervous system-related symptoms such as headache, epileptic seizures, disturbance of consciousness, and limb numbness and weakness, combined with skull CT, MRI and cerebrospinal fluid examinations showing abnormalities of the central nervous system, after excluding similar diseases such as primary nervous system diseases and infectious encephalopathy. |
| Haematological disturbance | Haematological disturbance can be diagnosed when the clinical manifestations are blood-related symptoms such as anemia, bleeding, fever and fatigue, combined with abnormal blood cell count, morphology or function shown by blood routine, bone marrow puncture and coagulation function examinations, after excluding similar etiologies such as infection, drug influence and hematological tumors. |
| Reynaud’s phenomenon | Reynaud’s phenomenon can be diagnosed when the clinical manifestation is the three-phase skin color change of “pallor-cyanosis-flushing” in the extremities (fingers, toes) after cold or emotional stimulation, accompanied by numbness, coldness or tingling, combined with vascular ultrasound showing peripheral vasospasm, after excluding similar diseases such as thromboangiitis obliterans and systemic sclerosis. |
| Cushingoid | Cushingoid can be diagnosed when the clinical manifestations are characteristic symptoms such as central obesity, skin striae, hirsutism, acne and hypertension, combined with abnormal elevation shown by cortisol level detection (blood, urine), after excluding primary Cushing’s syndrome such as Cushing’s disease and adrenal tumors, and confirming the presence of glucocorticoid use history or related incentives. |
| Antiphospholipid syndrom | Antiphospholipid Syndrome can be diagnosed when the clinical manifestations are characteristic symptoms such as thrombosis (venous or arterial thrombosis), recurrent abortion and thrombocytopenia, combined with persistent positive detection of antiphospholipid antibodies (anticardiolipin antibody, anti-β2 glycoprotein 1 antibody, etc.), after excluding other incentives such as infection, tumor and autoimmune diseases. |
| Thrombophlebitis | Thrombophlebitis can be diagnosed when the clinical manifestations are symptoms such as redness, swelling, pain and increased skin temperature in the distribution area of the affected vein, with palpable cord-like induration, combined with vascular ultrasound showing venous thrombosis and vascular wall inflammation, after excluding similar diseases such as deep vein thrombosis and cellulitis. |
| ANA | ANA is a heterogeneous group of autoantibodies directed against nuclear antigens, a key serological marker for systemic autoimmune diseases, particularly SLE, and is detected by IIF on HEp-2 cells, with a titer ≥1:100 defined as positive. |
| Anti-SSA | Anti-SSA antibody is an autoantibody targeting the Sjögren’s syndrome A (Ro) ribonucleoprotein, commonly associated with SLE, Sjögren’s syndrome, and neonatal lupus, and is detected by ELISA and immunoblotting. |
| Anti-SSB | Anti-SSB antibody is an autoantibody targeting the Sjögren’s syndrome B (La) ribonucleoprotein, often coexisting with anti-SSA antibodies in autoimmune diseases, and is detected by ELISA and immunoblotting. |
| Antiphospholipid antibody | Antiphospholipid antibody is a heterogeneous group of autoantibody that binds to anionic phospholipids or phospholipid-binding proteins, a core marker for antiphospholipid syndrome characterized by thrombosis and pregnancy morbidity, and is detected by ELISA, immunoblotting, and coagulation-based assays. |
| Leukopenia | Leukopenia is a hematological condition defined as a peripheral white blood cell count <4.0×10^9^/L, a common manifestation of SLE or immunosuppressive therapy, and detected by complete blood count analysis and bone marrow aspiration if necessary. |
| Anti-dsDNA antibody | Anti-dsDNA antibody is a highly specific autoantibody for SLE that targets double-stranded DNA, correlating with SLE disease activity, especially renal involvement, and is detected by ELISA. |
| Anticardiolipin antibody | Anticardiolipin antibody is a major subclass of antiphospholipid antibody targeting cardiolipin, a key component in the classification criteria for antiphospholipid syndrome, and is detected by ELISA. |
| Lupus anticoagulant | LA is a functional marker for antiphospholipid antibodies that prolong phospholipid-dependent coagulation tests, which is linked to increased thrombotic risk in antiphospholipid syndrome, and is detected by aPTT, lupus anticoagulant screening test, and confirmation test. |
| RF | RF is an autoantibody (predominantly IgM) targeting the Fc portion of IgG, associated with rheumatoid arthritis and other autoimmune diseases including SLE, and is detected by latex agglutination test and ELISA. |

MRI: magnetic resonance imaging; CT: computed tomography; SLEDAI: systemic lupus erythematosus disease activity index; SLE: systemic lupus erythematosus; CTA: computed tomography angiography; CNS: central nervous system; ANA: anti-nuclear antibodies; IIF: indirect immunofluorescence; Anti-SSA: anti-Sjogren Syndrome A antibody; Anti-SSB: anti-Sjogren Syndrome B antibody; Anti-dsDNA: Anti-double stranded DNA antibody; ELISA: enzyme-linked immunosorbent assay; aPTT: activated partial thromboplastin time; RF: rheumatoid factor.
