# Supplementary material for: Meta-analysis of factors for osteonecrosis in systemic lupus erythematosus: integration of comprehensive literatures and multicenter databases
Source: Front Immunol. 2026 Jul 2;17:1679237. doi: 10.3389/fimmu.2026.1679237 (PMC13372907; doi:10.3389/fimmu.2026.1679237)
Supplement: Supplementary file 1 [file DataSheet1.zip › Supplementary Material/Supplementary table 10.docx]

Supplementary table 10 Sensitivity analysis for diabetes mellitus in the meta-analysis.

| Sensitivity analysis | Heterogeneity (I^2^) | Combined effect size (95% CI) | P value |
| --- | --- | --- | --- |
| Omitting Abdelkawy, et al. 2022 | 24.7% | 1.153 (0.993, 1.339) | 0.0622 |
| Omitting Xiong, et al. 2022 | 27.7% | 1.186 (1.033, 1.362) | 0.0155 |
| Omitting Long, et al. 2021 | 27.8% | 1.185 (1.032, 1.362) | 0.0165 |
| Omitting Shaharir, et al. 2021 | 27.3% | 1.177 (1.022, 1.355) | 0.0233 |
| Omitting Dogan, et al. 2020 | 26.7% | 1.195 (1.041, 1.372) | 0.0115 |
| Omitting Tse, et al. 2016 | 16.9% | 1.168 (1.016, 1.342) | 0.0289 |
| Omitting Sekiya, et al. 2009 | 26.9% | 1.193 (1.039, 1.370) | 0.0123 |
| Omitting Mok, et al. 1998 | 26.4% | 1.184 (1.031, 1.360) | 0.0168 |
| Omitting Al Saleh, et al. 2010 | 25.9% | 1.200 (1.044, 1.379) | 0.1010 |
| Omitting Joo, et al. 2014 | 18.7% | 1.296 (1.116, 1.504) | 0.0007 |
| Omitting Calvo-Alen, et al. 2006 | 27.1% | 1.181 (1.028, 1.357) | 0.0191 |
| Omitting Lee, et al. 2013 | 26.6% | 1.194 (1.040, 1.371) | 0.0120 |
| Omitting Sayarlioglu, et al. 2010 | 26.1% | 1.178 (1.025, 1.353) | 0.0214 |
| Omitting Kunyakham, et al. 2012 | 24.5% | 1.209 (1.009, 1.450) | 0.0399 |
| Omitting Wu, et al. 2014 | 26.9% | 1.195 (1.040, 1.372) | 0.0119 |
| Omitting Li, et al. 2021 | 26.7% | 1.202 (1.045, 1.382) | 0.0101 |
| Omitting Vílchez-Oya, et al. 2019 | 22.8% | 1.177 (1.025, 1.352) | 0.0214 |
| Omitting Xu, et al. 2024 | 28.0% | 1.187 (1.033, 1.364) | 0.0159 |
| Omitting Chen, et al. 2021 | 27.5% | 1.192 (1.038, 1.368) | 0.0130 |
| Omitting Wang, et al. 2009 | 27.2% | 1.180 (1.027, 1.357) | 0.0196 |
| Omitting AHSMU. 2023 | 0.0% | 1.131 (0.980, 1.305) | 0.0911 |
| Omitting WCHSCU. 2020 | 24.0% | 1.207 (1.052, 1.386) | 0.0075 |
| Omitting MHMU. 2023 | 27.4% | 1.196 (1.041, 1.375) | 0.0117 |
| Before omitting | 24.6% | 1.188 (1.035, 1.365) | 0.0144 |

CI: confidence interval; AHSMU: Affiliated Hospital of Southwest Medical University; WCHSCU: West China Hospital of Sichuan University; MHMU: Minda Hospital of Hubei Minzu University.
