# Supplementary material for: Meta-analysis of factors for osteonecrosis in systemic lupus erythematosus: integration of comprehensive literatures and multicenter databases
Source: Front Immunol. 2026 Jul 2;17:1679237. doi: 10.3389/fimmu.2026.1679237 (PMC13372907; doi:10.3389/fimmu.2026.1679237)
Supplement: Supplementary file 1 [file DataSheet1.zip › Supplementary Material/Supplementary table 11.docx]

Supplementary table 11 Sensitivity analysis for oral ulcers in the meta-analysis.

| Sensitivity analysis | Heterogeneity (I^2^) | Combined effect size (95% CI) | P value |
| --- | --- | --- | --- |
| Omitting Xiong, et al. 2022 | 58.2% | 1.113 (0.959, 1.292) | 0.1583 |
| Omitting Long, et al. 2021 | 59.4% | 1.127 (0.966, 1.316) | 0.1297 |
| Omitting Dogan, et al. 2020 | 58.4% | 1.136 (0.979, 1.319) | 0.0939 |
| Omitting Hisada, et al. 2018 | 59.2% | 1.117 (0.962, 1.297) | 0.1482 |
| Omitting Tse, et al. 2016 | 57.5% | 1.154 (0.992, 1.342) | 0.0637 |
| Omitting Kuroda, et al. 2015 | 58.4% | 1.135 (0.978, 1.318) | 0.0954 |
| Omitting Mok, et al. 1998 | 59.4% | 1.125 (0.969, 1.307) | 0.1228 |
| Omitting Al Saleh, et al. 2010 | 59.3% | 1.118 (0.963, 1.298) | 0.1438 |
| Omitting Griffiths, et al. 1979 | 59.2% | 1.127 (0.971, 1.309) | 0.1149 |
| Omitting Weiner, et al. 1989 | 59.4% | 1.124 (0.968, 1.305) | 0.1240 |
| Omitting Lee, et al. 2013 | 59.3% | 1.112 (0.955, 1.295) | 0.1702 |
| Omitting Faezi, et al. 2014 | 37.4% | 1.295 (1.105, 1.517) | 0.0014 |
| Omitting Sayarlioglu, et al. 2010 | 55.9% | 1.078 (0.925, 1.256) | 0.3372 |
| Omitting Smith, et al. 1976 | 59.3% | 1.121 (0.965, 1.301) | 0.1344 |
| Omitting Li, et al. 2008 | 59.3% | 1.116 (0.960, 1.297) | 0.1518 |
| Omitting Qi, et al. 2010 | 55.5% | 1.081 (0.929, 1.258) | 0.3159 |
| Omitting Xuan, et al. 2011 | 59.0% | 1.111 (0.956, 1.292) | 0.1703 |
| Omitting Shen, et al. 2012 | 58.6% | 1.113 (0.959, 1.293) | 0.1600 |
| Omitting Shi, et al. 2013 | 59.4% | 1.117 (0.961, 1.299) | 0.1502 |
| Omitting Wu, et al. 2014 | 58.1% | 1.109 (0.954, 1.288) | 0.1772 |
| Omitting Lin, et al. 2014 | 59.4% | 1.122 (0.966, 1.304) | 0.1317 |
| Omitting Wang, et al. 2018 | 59.4% | 1.128 (0.970, 1.311) | 0.1186 |
| Omitting Li, et al. 2021 | 58.8% | 1.100 (0.944, 1.283) | 0.2213 |
| Omitting Zhang, et al. 2008 | 52.7% | 1.077 (0.926, 1.252) | 0.3387 |
| Omitting Liu, et al. 2011 | 57.1% | 1.094 (0.941, 1.272) | 0.2435 |
| Omitting Li, et al. 2014 | 58.9% | 1.137 (0.978, 1.322) | 0.0948 |
| Omitting Vilchez-Oya, et al. 2019 | 58.4% | 1.115 (0.961, 1.294) | 0.1517 |
| Omitting Kwon, et al. 2018 | 56.9% | 1.192 (1.016, 1.397) | 0.0308 |
| Omitting Xu, et al. 2024 | 59.4% | 1.120 (0.961, 1.305) | 0.1470 |
| Omitting Wang, et al. 2009 | 59.0% | 1.109 (0.950, 1.290) | 0.1776 |
| Omitting AHSMU. 2023 | 58.7% | 1.135 (0.977, 1.317) | 0.0970 |
| Before omitting | 58.0% | 1.123 (0.968, 1.303) | 0.1256 |

CI: confidence interval; AHSMU: Affiliated Hospital of Southwest Medical University.
