# Supplementary material for: Meta-analysis of factors for osteonecrosis in systemic lupus erythematosus: integration of comprehensive literatures and multicenter databases
Source: Front Immunol. 2026 Jul 2;17:1679237. doi: 10.3389/fimmu.2026.1679237 (PMC13372907; doi:10.3389/fimmu.2026.1679237)
Supplement: Supplementary file 1 [file DataSheet1.zip › Supplementary Material/Supplementary table 12.docx]

Supplementary table 12 Sensitivity analysis for pleuritis in the meta-analysis.

| Sensitivity analysis | Heterogeneity (I^2^) | Combined effect size (95% CI) | P value |
| --- | --- | --- | --- |
| Omitting Massardo, et al. 1992 | 0.0% | 2.506 (1.399, 4.489) | 0.0020 |
| Omitting Sayarlioglu, et al. 2010 | 0.0% | 1.592 (0.769, 3.296) | 0.2108 |
| Omitting Smith, et al. 1976 | 0.0% | 2.346 (1.398, 3.936) | 0.0012 |
| Omitting Xuan, et al. 2011 | 0.0% | 2.240 (1.310, 3.829) | 0.0032 |
| Omitting Vilchez-Oya, et al. 2019 | 0.0% | 2.313 (1.387, 3.855) | 0.0013 |
| Omitting AHSMU. 2023 | 0.0% | 2.192 (1.319, 3.642) | 0.0025 |
| Before omitting | 0.0% | 2.225 (1.346, 3.679) | 0.0018 |

CI: confidence interval; AHSMU: Affiliated Hospital of Southwest Medical University.
