# Supplementary material for: Meta-analysis of factors for osteonecrosis in systemic lupus erythematosus: integration of comprehensive literatures and multicenter databases
Source: Front Immunol. 2026 Jul 2;17:1679237. doi: 10.3389/fimmu.2026.1679237 (PMC13372907; doi:10.3389/fimmu.2026.1679237)
Supplement: Supplementary file 1 [file DataSheet1.zip › Supplementary Material/Supplementary table 13.docx]

Supplementary table 13 Sensitivity analysis for malar rash in the meta-analysis.

| Sensitivity analysis | Heterogeneity (I^2^) | Combined effect size (95% CI) | P value |
| --- | --- | --- | --- |
| Omitting Xiong, et al. 2022 | 48.3% | 1.279 (1.119, 1.461) | 0.0003 |
| Omitting Long, et al. 2021 | 48.3% | 1.272 (1.107, 1.462) | 0.0007 |
| Omitting Dogan, et al. 2020 | 48.3% | 1.274 (1.115, 1.455) | 0.0004 |
| Omitting Hisada, et al. 2018 | 46.0% | 1.254 (1.097, 1.434) | 0.0009 |
| Omitting Jokar, et al. 2016 | 46.6% | 1.255 (1.097, 1.436) | 0.0009 |
| Omitting Kuroda, et al. 2015 | 46.9% | 1.289 (1.128, 1.473) | 0.0002 |
| Omitting Watanabe, et al. 1997 | 48.3% | 1.273 (1.114, 1.454) | 0.0004 |
| Omitting Mok, et al. 1998 | 42.0% | 1.309 (1.146, 1.497) | <0.0001 |
| Omitting Al Saleh, et al. 2010 | 47.9% | 1.269 (1.110, 1.449) | 0.0004 |
| Omitting Massardo, et al. 1992 | 46.1% | 1.258 (1.100, 1.437) | 0.0008 |
| Omitting Ono, et al. 1992 | 47.9% | 1.280 (1.121, 1.462) | 0.0003 |
| Omitting Griffiths, et al. 1979 | 47.7% | 1.282 (1.123, 1.464) | 0.0003 |
| Omitting Weiner, et al. 1989 | 47.3% | 1.268 (1.101, 1.448) | 0.0005 |
| Omitting Lee, et al. 2013 | 46.8% | 1.257 (1.099, 1.438) | 0.0008 |
| Omitting Faezi, et al. 2014 | 6.8% | 1.408 (1.225, 1.617) | <0.0001 |
| Omitting Sayarlioglu, et al. 2010 | 47.5% | 1.259 (1.101, 1.441) | 0.0008 |
| Omitting Zizic, et al. 1985 | 48.0% | 1.268 (1.110, 1.449) | 0.0005 |
| Omitting Kunyakham, et al. 2012 | 47.4% | 1.257 (1.097, 1.440) | 0.0010 |
| Omitting Smith, et al. 1976 | 47.5% | 1.270 (1.112, 1.450) | 0.0004 |
| Omitting Qi, et al. 2010 | 47.9% | 1.285 (1.124, 1.470) | 0.0003 |
| Omitting Xuan, et al. 2011 | 46.3% | 1.254 (1.097, 1.435) | 0.0009 |
| Omitting Wu, et al. 2014 | 48.3% | 1.274 (1.115, 1.455) | 0.0004 |
| Omitting Li, et al. 2021 | 47.6% | 1.297 (1.131, 1.487) | 0.0002 |
| Omitting Lei, et al. 2024 | 47.1% | 1.257 (1.098, 1.438) | 0.0009 |
| Omitting Liu, et al. 2011 | 48.3% | 1.278 (1.118, 1.461) | 0.0003 |
| Omitting Li, et al. 2014 | 48.3% | 1.271 (1.112, 1.454) | 0.0005 |
| Omitting Kwon, et al. 2018 | 46.8% | 1.240 (1.077, 1.426) | 0.0027 |
| Omitting Xu, et al. 2024 | 48.3% | 1.280 (1.115, 1.469) | 0.0005 |
| Omitting Chen, et al. 2021 | 44.7% | 1.241 (1.084, 1.421) | 0.0017 |
| Omitting Wang, et al. 2009 | 47.2% | 1.291 (1.129, 1.476) | 0.0002 |
| Omitting AHSMU. 2023 | 47.8% | 1.289 (1.126, 1.475) | 0.0002 |
| Before omitting | 46.6% | 1.275 (1.117, 1.455) | 0.0003 |

CI: confidence interval; AHSMU: Affiliated Hospital of Southwest Medical University.
