# Supplementary material for: Meta-analysis of factors for osteonecrosis in systemic lupus erythematosus: integration of comprehensive literatures and multicenter databases
Source: Front Immunol. 2026 Jul 2;17:1679237. doi: 10.3389/fimmu.2026.1679237 (PMC13372907; doi:10.3389/fimmu.2026.1679237)
Supplement: Supplementary file 1 [file DataSheet1.zip › Supplementary Material/Supplementary table 14.docx]

Supplementary table 14 Sensitivity analysis for vasculitis in the meta-analysis.

| Sensitivity analysis | Heterogeneity (I^2^) | Combined effect size (95% CI) | P value |
| --- | --- | --- | --- |
| Omitting Xiong, et al. 2022 | 53.7% | 2.348 (1.989, 2.771) | <0.0001 |
| Omitting Shaharir, et al. 2021 | 53.1% | 2.439 (2.056, 2.893) | <0.0001 |
| Omitting Dogan, et al. 2020 | 53.2% | 2.400 (2.033, 2.833) | <0.0001 |
| Omitting Tse, et al. 2016 | 54.6% | 2.369 (2.000, 2.808) | <0.0001 |
| Omitting Jokar, et al. 2016 | 54.4% | 2.382 (2.015, 2.816) | <0.0001 |
| Omitting Mok, et al. 1998 | 45.6% | 2.527 (2.134, 2.993) | <0.0001 |
| Omitting Al Saleh, et al. 2010 | 54.5% | 2.365 (2.003, 2.793) | <0.0001 |
| Omitting Massardo, et al. 1992 | 54.5% | 2.375 (2.009, 2.807) | <0.0001 |
| Omitting Hamijoyo, et al. 2008 | 53.3% | 2.319 (1.960, 2.743) | <0.0001 |
| Omitting Weiner, et al. 1989 | 53.7% | 2.392 (2.027, 2.824) | <0.0001 |
| Omitting Lee, et al. 2013 | 54.3% | 2.394 (2.022, 2.833) | <0.0001 |
| Omitting Fialho, et al. 2007 | 54.3% | 2.382 (2.018, 2.812) | <0.0001 |
| Omitting Sayarlioglu, et al. 2010 | 54.5% | 2.359 (1.994, 2.791) | <0.0001 |
| Omitting Gladman, et al. 2001 | 53.3% | 2.438 (2.057, 2.891) | <0.0001 |
| Omitting Kunyakham, et al. 2012 | 46.0% | 2.274 (1.921, 2.691) | <0.0001 |
| Omitting Mont, et al. 1997 | 54.2% | 2.342 (1.981, 2.770) | <0.0001 |
| Omitting Liu, et al. 2022 | 52.0% | 2.314 (1.958, 2.734) | <0.0001 |
| Omitting Li, et al. 2008 | 53.4% | 2.329 (1.971, 2.752) | <0.0001 |
| Omitting Qi, et al. 2010 | 54.6% | 2.374 (2.005, 2.810) | <0.0001 |
| Omitting Shen, et al. 2012 | 54.1% | 2.350 (1.990, 2.774) | <0.0001 |
| Omitting Shi, et al. 2013 | 53.6% | 2.323 (1.964, 2.748) | <0.0001 |
| Omitting Wu, et al. 2014 | 53.9% | 2.348 (1.989, 2.773) | <0.0001 |
| Omitting Wang, et al. 2018 | 53.1% | 2.320 (1.963, 2.743) | <0.0001 |
| Omitting Li, et al. 2021 | 54.3% | 2.346 (1.985, 2.773) | <0.0001 |
| Omitting Zhang, et al. 2008 | 46.6% | 2.248 (1.901, 2.659) | <0.0001 |
| Omitting Liu, et al. 2011 | 54.3% | 2.345 (1.982, 2.773) | <0.0001 |
| Omitting Li, et al. 2014 | 51.8% | 2.429 (2.056, 2.869) | <0.0001 |
| Omitting Tang, et al. 1999 | 54.3% | 2.350 (1.989, 2.777) | <0.0001 |
| Omitting Shen, et al. 2005 | 54.5% | 2.379 (2.013, 2.812) | <0.0001 |
| Omitting Gladman, et al. 2018 | 46.8% | 2.625 (2.202, 3.129) | <0.0001 |
| Omitting Wang, et al. 2009 | 53.8% | 2.338 (1.979, 2.762) | <0.0001 |
| Omitting AHSMU. 2023 | 54.6% | 2.369 (2.007, 2.797) | <0.0001 |
| Before omitting | 53.1% | 2.370 (2.009, 2.795) | <0.0001 |

CI: confidence interval; AHSMU: Affiliated Hospital of Southwest Medical University.
