# Supplementary material for: Meta-analysis of factors for osteonecrosis in systemic lupus erythematosus: integration of comprehensive literatures and multicenter databases
Source: Front Immunol. 2026 Jul 2;17:1679237. doi: 10.3389/fimmu.2026.1679237 (PMC13372907; doi:10.3389/fimmu.2026.1679237)
Supplement: Supplementary file 1 [file DataSheet1.zip › Supplementary Material/Supplementary table 15.docx]

Supplementary table 15 Sensitivity analysis for serositis in the meta-analysis.

| Sensitivity analysis | Heterogeneity (I^2^) | Combined effect size (95% CI) | P value |
| --- | --- | --- | --- |
| Omitting Cheng, et al. 2023 | 0.0% | 1.474 (1.218, 1.783) | <0.0001 |
| Omitting Long, et al. 2021 | 0.0% | 1.550 (1.285, 1.869) | <0.0001 |
| Omitting Dogan, et al. 2020 | 0.0% | 1.528 (1.276, 1.829) | <0.0001 |
| Omitting Hisada, et al. 2018 | 0.0% | 1.524 (1.272, 1.827) | <0.0001 |
| Omitting Tse, et al. 2016 | 0.0% | 1.581 (1.316, 1.901) | <0.0001 |
| Omitting Jokar, et al. 2016 | 0.0% | 1.516 (1.266, 1.816) | <0.0001 |
| Omitting Kuroda, et al. 2015 | 0.0% | 1.524 (1.273, 1.825) | <0.0001 |
| Omitting Mok, et al. 1998 | 0.0% | 1.574 (1.319, 1.889) | <0.0001 |
| Omitting Al Saleh, et al. 2010 | 0.0% | 1.499 (1.252, 1.795) | <0.0001 |
| Omitting Ono, et al. 1992 | 0.0% | 1.517 (1.269, 1.815) | <0.0001 |
| Omitting Lee, et al. 2013 | 0.0% | 1.501 (1.250, 1.804) | <0.0001 |
| Omitting Sayarlioglu, et al. 2010 | 0.0% | 1.450 (1.204, 1.746) | <0.0001 |
| Omitting Kunyakham, et al. 2012 | 0.0% | 1.514 (1.264, 1.814) | <0.0001 |
| Omitting Li, et al. 2021 | 0.0% | 1.488 (1.232, 1.797) | <0.0001 |
| Omitting Li, et al. 2014 | 0.0% | 1.489 (1.241, 1.787) | <0.0001 |
| Omitting Kwon, et al. 2018 | 0.0% | 1.467 (1.207, 1.792) | 0.0002 |
| Omitting Xu, et al. 2024 | 0.0% | 1.552 (1.290, 1.867) | <0.0001 |
| Omitting Chen, et al. 2021 | 0.0% | 1.497 (1.248, 1.795) | <0.0001 |
| Before omitting | 0.0% | 1.514 (1.266, 1.810) | <0.0001 |

CI: confidence interval.
