# Supplementary material for: Meta-analysis of factors for osteonecrosis in systemic lupus erythematosus: integration of comprehensive literatures and multicenter databases
Source: Front Immunol. 2026 Jul 2;17:1679237. doi: 10.3389/fimmu.2026.1679237 (PMC13372907; doi:10.3389/fimmu.2026.1679237)
Supplement: Supplementary file 1 [file DataSheet1.zip › Supplementary Material/Supplementary table 16.docx]

Supplementary table 16 Sensitivity analysis for nephritis in the meta-analysis.

| Sensitivity analysis | Heterogeneity (I^2^) | Combined effect size (95% CI) | P value |
| --- | --- | --- | --- |
| Omitting Xiong, et al. 2022 | 46.6% | 1.433 (1.233, 1.666) | <0.0001 |
| Omitting Long, et al. 2021 | 52.5% | 1.407 (1.200, 1.649) | <0.0001 |
| Omitting Shaharir, et al. 2021 | 53.8% | 1.502 (1.287, 1.752) | <0.0001 |
| Omitting Jokar, et al. 2016 | 52.0% | 1.432 (1.230, 1.668) | <0.0001 |
| Omitting Yang, et al. 2015 | 43.3% | 1.406 (1.207, 1.637) | <0.0001 |
| Omitting Ono, et al. 1992 | 55.3% | 1.470 (1.265, 1.707) | <0.0001 |
| Omitting Nagasawa, et al. 2005 | 55.7% | 1.463 (1.259, 1.700) | <0.0001 |
| Omitting Gladman, et al. 2001 | 53.8% | 1.494 (1.282, 1.740) | <0.0001 |
| Omitting Kunyakham, et al. 2012 | 55.4% | 1.485 (1.270, 1.736) | <0.0001 |
| Omitting Xuan, et al. 2011 | 55.7% | 1.460 (1.254, 1.700) | <0.0001 |
| Omitting Li, et al. 2021 | 54.9% | 1.494 (1.278, 1.746) | <0.0001 |
| Omitting Vilchez-Oya, et al. 2019 | 55.8% | 1.466 (1.262, 1.702) | <0.0001 |
| Omitting Xu, et al. 2024 | 53.0% | 1.412 (1.205, 1.654) | <0.0001 |
| Omitting Chen, et al. 2021 | 54.8% | 1.443 (1.238, 1.683) | <0.0001 |
| Omitting AHSMU. 2023 | 40.0% | 1.588 (1.356, 1.861) | <0.0001 |
| Omitting WCHSCU. 2020 | 53.1% | 1.510 (1.293, 1.762) | <0.0001 |
| Omitting MHMU. 2023 | 55.8% | 1.471 (1.253, 1.726) | <0.0001 |
| Before omitting | 52.8% | 1.466 (1.262, 1.702) | <0.0001 |

CI: confidence interval; AHSMU: Affiliated Hospital of Southwest Medical University; WCHSCU: West China Hospital of Sichuan University; MHMU: Minda Hospital of Hubei Minzu University.
