# Supplementary material for: Meta-analysis of factors for osteonecrosis in systemic lupus erythematosus: integration of comprehensive literatures and multicenter databases
Source: Front Immunol. 2026 Jul 2;17:1679237. doi: 10.3389/fimmu.2026.1679237 (PMC13372907; doi:10.3389/fimmu.2026.1679237)
Supplement: Supplementary file 1 [file DataSheet1.zip › Supplementary Material/Supplementary table 17.docx]

Supplementary table 17 Sensitivity analysis for renal involvement in the meta-analysis.

| Sensitivity analysis | Heterogeneity (I^2^) | Combined effect size (95% CI) | P value |
| --- | --- | --- | --- |
| Omitting Cheng, et al. 2023 | 41.8% | 1.501 (1.303, 1.730) | <0.0001 |
| Omitting Dogan, et al. 2020 | 48.2% | 1.402 (1.226, 1.603) | <0.0001 |
| Omitting Hisada, et al. 2018 | 48.6% | 1.418 (1.239, 1.622) | <0.0001 |
| Omitting Tse, et al. 2016 | 41.7% | 1.355 (1.183, 1.553) | <0.0001 |
| Omitting Kuroda, et al. 2015 | 47.9% | 1.423 (1.244, 1.628) | <0.0001 |
| Omitting Sheikh, et al. 1998 | 48.8% | 1.412 (1.236, 1.619) | <0.0001 |
| Omitting Watanabe, et al. 1997 | 48.2% | 1.417 (1.240, 1.620) | <0.0001 |
| Omitting Mok, et al. 1998 | 47.2% | 1.388 (1.212, 1.589) | <0.0001 |
| Omitting Al Saleh, et al. 2010 | 47.5% | 1.398 (1.223, 1.598) | <0.0001 |
| Omitting Massardo, et al. 1992 | 48.8% | 1.408 (1.231, 1.610) | <0.0001 |
| Omitting Joo, et al. 2014 | 48.4% | 1.387 (1.199, 1.604) | <0.0001 |
| Omitting Nagasawa, et al. 1989 | 47.7% | 1.400 (1.224, 1.600) | <0.0001 |
| Omitting Weiner, et al. 1989 | 47.0% | 1.421 (1.243, 1.624) | <0.0001 |
| Omitting Lee, et al. 2013 | 48.8% | 1.408 (1.230, 1.612) | <0.0001 |
| Omitting Faezi, et al. 2014 | 48.8% | 1.408 (1.229, 1.613) | <0.0001 |
| Omitting Sayarlioglu, et al. 2010 | 48.0% | 1.392 (1.215, 1.595) | <0.0001 |
| Omitting Prasad, et al. 2007 | 48.8% | 1.412 (1.235, 1.615) | <0.0001 |
| Omitting Uea-areewongsa, et al. 2009 | 43.8% | 1.389 (1.215, 1.588) | <0.0001 |
| Omitting Liu, et al. 2022 | 48.1% | 1.422 (1.244, 1.627) | <0.0001 |
| Omitting Wu, et al. 2014 | 48.3% | 1.418 (1.246, 1.622) | <0.0001 |
| Omitting Lei, et al. 2024 | 47.0% | 1.436 (1.254, 1.644) | <0.0001 |
| Omitting Li, et al. 2014 | 48.5% | 1.401 (1.224, 1.603) | <0.0001 |
| Omitting Tang, et al. 1999 | 48.8% | 1.410 (1.233, 1.613) | <0.0001 |
| Omitting Shen, et al. 2005 | 45.2% | 1.442 (1.260, 1.651) | <0.0001 |
| Omitting Gladman, et al. 2018 | 41.6% | 1.491 (1.297, 1.715) | <0.0001 |
| Omitting Kwon, et al. 2018 | 36.4% | 1.305 (1.133, 1.503) | 0.0002 |
| Omitting Chen, et al. 2021 | 47.6% | 1.401 (1.226, 1.602) | <0.0001 |
| Omitting Wang, et al. 2009 | 45.7% | 1.433 (1.253, 1.639) | <0.0001 |
| Before omitting | 46.9% | 1.410 (1.235, 1.611) | <0.0001 |

CI: confidence interval.
