# Supplementary material for: Meta-analysis of factors for osteonecrosis in systemic lupus erythematosus: integration of comprehensive literatures and multicenter databases
Source: Front Immunol. 2026 Jul 2;17:1679237. doi: 10.3389/fimmu.2026.1679237 (PMC13372907; doi:10.3389/fimmu.2026.1679237)
Supplement: Supplementary file 1 [file DataSheet1.zip › Supplementary Material/Supplementary table 18.docx]

Supplementary table 18 Sensitivity analysis for gastrointestinal involvement in the meta-analysis.

| Sensitivity analysis | Heterogeneity (I^2^) | Combined effect size (95% CI) | P value |
| --- | --- | --- | --- |
| Omitting Long, et al. 2021 | 0.0% | 2.029 (1.467, 2.806) | <0.0001 |
| Omitting Tse, et al. 2016 | 0.0% | 2.111 (1.509, 2.953) | <0.0001 |
| Omitting Kunyakham, et al. 2012 | 0.0% | 1.891 (1.323, 2.704) | 0.0005 |
| Omitting Li, et al. 2021 | 0.0% | 1.930 (1.367, 2.724) | 0.0002 |
| Omitting Xu, et al. 2024 | 0.0% | 2.035 (1.324, 3.127) | 0.0012 |
| Before omitting | 0.0% | 1.998 (1.457, 2.741) | 0.0001 |

CI: confidence interval.
