# Supplementary material for: Meta-analysis of factors for osteonecrosis in systemic lupus erythematosus: integration of comprehensive literatures and multicenter databases
Source: Front Immunol. 2026 Jul 2;17:1679237. doi: 10.3389/fimmu.2026.1679237 (PMC13372907; doi:10.3389/fimmu.2026.1679237)
Supplement: Supplementary file 1 [file DataSheet1.zip › Supplementary Material/Supplementary table 19.docx]

Supplementary table 19 Sensitivity analysis for CNS involvement in the meta-analysis.

| Sensitivity analysis | Heterogeneity (I^2^) | Combined effect size (95% CI) | P value |
| --- | --- | --- | --- |
| Omitting Jokar, et al. 2016 | 23.0% | 2.042 (1.423, 2.929) | 0.0001 |
| Omitting Kuroda, et al. 2015 | 8.8% | 2.125 (1.521, 2.970) | <0.0001 |
| Omitting Sheikh, et al. 1998 | 22.0% | 2.047 (1.465, 2.859) | <0.0001 |
| Omitting Mok, et al. 1998 | 2.3% | 1.759 (1.224, 2.530) | 0.0023 |
| Omitting Massardo, et al. 1992 | 3.9% | 2.179 (1.550, 3.063) | <0.0001 |
| Omitting Yang, et al. 2015 | 15.4% | 1.822 (1.268, 2.617) | 0.0012 |
| Omitting Griffiths, et al. 1979 | 8.9% | 2.125 (1.521, 2.971) | <0.0001 |
| Omitting Nagasawa, et al. 2005 | 18.4% | 1.935 (1.382, 2.711) | 0.0001 |
| Omitting Nagasawa, et al. 1989 | 18.1% | 2.105 (1.492, 2.970) | <0.0001 |
| Omitting Weiner, et al. 1989 | 19.7% | 1.949 (1.394, 2.725) | <0.0001 |
| Omitting Sayarlioglu, et al. 2010 | 23.1% | 2.038 (1.438, 2.888) | <0.0001 |
| Omitting Smith, et al. 1976 | 23.4% | 1.997 (1.435, 2.779) | <0.0001 |
| Before omitting | 16.2% | 2.010 (1.448, 2.792) | <0.0001 |

CI: confidence interval; CNS, central nervous system.
