# Supplementary material for: Meta-analysis of factors for osteonecrosis in systemic lupus erythematosus: integration of comprehensive literatures and multicenter databases
Source: Front Immunol. 2026 Jul 2;17:1679237. doi: 10.3389/fimmu.2026.1679237 (PMC13372907; doi:10.3389/fimmu.2026.1679237)
Supplement: Supplementary file 1 [file DataSheet1.zip › Supplementary Material/Supplementary table 2.docx]

Supplementary table 2 General information of the patients from AHSMU cohort.

| Variable | Total (n=4727) | SLE-ON (n=82) | SLE-non-ON (n=4645) | P value |
| --- | --- | --- | --- | --- |
| Demographic and clinical characteristics |  |  |  |  |
| Female, N (%) | 4069 (86.08) | 58 (70.73) | 4011 (86.35) | <0.001 |
| Age, year | 42.60±15.57 | 44.67±13.23 | 42.57±15.60 | 0.225 |
| Epilepsy (+), N (%) | 21 (0.44) | 0 (0.00) | 21 (0.45) | 0.542 |
| Arthritis (+), N (%) | 500 (10.58) | 7 (8.54) | 493 (10.61) | 0.544 |
| Alopecia (+), N (%) | 280 (5.92) | 2 (2.44) | 278 (5.98) | 0.178 |
| Neuropsychiatric lupus (+), N (%) | 19 (0.40) | 0 (0.00) | 19 (0.41) | 0.562 |
| Hypertension (+), N (%) | 1323 (27.99) | 30 (36.59) | 1293 (27.84) | 0.080 |
| Diabetes mellitus (+), N (%) | 207 (4.38) | 11 (13.41) | 196 (4.22) | <0.001 |
| Oral ulcers (+), N (%) | 118 (2.50) | 0 (0.00) | 118 (2.54) | 0.269 |
| Pleuritis (+), N (%) | 4 (0.08) | 0 (0.00) | 4 (0.09) | 1.000 |
| Malar rash (+), N (%) | 537 (11.36) | 9 (10.98) | 528 (11.37) | 0.912 |
| Vasculitis (+), N (%) | 49 (1.04) | 2 (2.44) | 47 (1.01) | 0.475 |
| Pericarditis (+), N (%) | 26 (0.55) | 0 (0.00) | 26 (0.56) | 1.000 |
| Nephritis (+), N (%) | 3108 (65.75) | 49 (59.76) | 3059 (65.86) | 0.249 |
| Anemia (+), N (%) | 703 (14.87) | 6 (7.32) | 697 (15.01) | 0.052 |
| Cataract (+), N (%) | 39 (0.83) | 0 (0.00) | 39 (0.84) | 0.828 |
| Pulmonary arterial hypertension (+), N (%) | 134 (2.83) | 1 (1.22) | 133 (2.86) | 0.580 |
| Hematologic involvement (+), N (%) | 1671 (35.35) | 23 (28.05) | 1648 (35.48) | 0.163 |
| Reynaud’s phenomenon (+), N (%) | 55 (1.16) | 1 (1.22) | 54 (1.16) | 1.000 |
| Cushingoid (+), N (%) | 4 (0.08) | 0 (0.00) | 4 (0.09) | 1.000 |
| Osteoporosis (+), N (%) | 535 (11.32) | 33 (40.24) | 502 (10.81) | <0.001 |
| Antiphospholipid syndrome (+), N (%) | 301 (6.37) | 3 (3.66) | 298 (6.42) | 0.311 |
| Livedo reticularis (+), N (%) | 2 (0.04) | 0 (0.00) | 2 (0.04) | 1.000 |
| Sjögren’s syndrome (+), N (%) | 144 (3.05) | 7 (8.54) | 137 (2.95) | 0.009 |
| Laboratory characteristics |  |  |  |  |
| Proteinurial (+), N (%) | 3053 (64.59) | 49 (59.76) | 3004 (64.67) | 0.356 |
| ANA (+), N (%) | 4558 (96.42) | 80 (97.56) | 4478 (96.40) | 0.796 |
| Anti-dsDNA (+), N (%) | 929 (19.65) | 13 (15.85) | 916 (19.72) | 0.382 |
| Anti-Sm (+), N (%) | 1201 (25.41) | 17 (20.73) | 1184 (25.49) | 0.327 |
| Anti-SSA (+), N (%) | 3176 (67.19) | 55 (67.07) | 3121 (67.19) | 0.982 |
| Anti-SSB (+), N (%) | 542 (11.47) | 9 (10.98) | 533 (11.47) | 0.888 |
| Anti-RNP (+), N (%) | 2002 (42.35) | 39 (47.56) | 1963 (42.26) | 0.336 |
| Lupus anticoagulant (+), N (%) | 2655 (56.17) | 42 (51.22) | 2613 (56.25) | 0.362 |
| Leukopenia (+), N (%) | 128 (2.71) | 1 (1.22) | 127 (2.73) | 0.621 |
| Thrombocytopenia (+), N (%) | 201 (4.25) | 0 (0.00) | 201 (4.33) | 0.099 |
| RF (+), N (%) | 710 (15.02) | 10 (12.20) | 700 (15.07) | 0.470 |
| Hypocomplementemia (+), N (%) | 2614 (55.30) | 28 (34.15) | 2586 (55.67) | <0.001 |

AHSMU: Affiliated Hospital of Southwest Medical University; SLE: systemic lupus erythematosus; ON: osteonecrosis; SD: standard deviation; ANA: antinuclear antibody; Anti-dsDNA: anti-double stranded DNA antibody; Anti-Sm: anti-smith antibody; Anti-SSA: anti-Sjogren Syndrome A antibody; Anti-SSB: anti-Sjogren Syndrome B antibody; Anti-RNP: anti-ribonucleoprotein antibody; RF: rheumatoid factor
