# Supplementary material for: Meta-analysis of factors for osteonecrosis in systemic lupus erythematosus: integration of comprehensive literatures and multicenter databases
Source: Front Immunol. 2026 Jul 2;17:1679237. doi: 10.3389/fimmu.2026.1679237 (PMC13372907; doi:10.3389/fimmu.2026.1679237)
Supplement: Supplementary file 1 [file DataSheet1.zip › Supplementary Material/Supplementary table 20.docx]

Supplementary table 20 Sensitivity analysis for musculoskeletal in the meta-analysis.

| Sensitivity analysis | Heterogeneity (I^2^) | Combined effect size (95% CI) | P value |
| --- | --- | --- | --- |
| Omitting Mok, et al. 1998 | 0.0% | 2.400 (1.288, 4.475) | 0.0059 |
| Omitting Li, et al. 2008 | 29.1% | 2.217 (1.197, 4.106) | 0.0114 |
| Omitting Xuan, et al. 2011 | 39.4% | 1.776 (0.873, 3.615) | 0.1129 |
| Omitting Wu, et al. 2014 | 32.7% | 1.767 (0.924, 3.378) | 0.0851 |
| Omitting Lei, et al. 2024 | 32.7% | 1.662 (0.852, 3.245) | 0.1364 |
| Omitting Li, et al. 2014 | 36.8% | 2.183 (1.164, 4.097) | 0.0150 |
| Before omitting | 28.7% | 2.002 (1.110, 3.613) | 0.0212 |

CI: confidence interval.
