# Supplementary material for: Meta-analysis of factors for osteonecrosis in systemic lupus erythematosus: integration of comprehensive literatures and multicenter databases
Source: Front Immunol. 2026 Jul 2;17:1679237. doi: 10.3389/fimmu.2026.1679237 (PMC13372907; doi:10.3389/fimmu.2026.1679237)
Supplement: Supplementary file 1 [file DataSheet1.zip › Supplementary Material/Supplementary table 21.docx]

Supplementary table 21 Sensitivity analysis for hematologic involvement in the meta-analysis.

| Sensitivity analysis | Heterogeneity (I^2^) | Combined effect size (95% CI) | P value |
| --- | --- | --- | --- |
| Omitting Cheng, et al. 2023 | 20.1% | 1.138 (0.963, 1.345) | 0.1292 |
| Omitting Xiong, et al. 2022 | 24.2% | 1.180 (1.010, 1.379) | 0.0368 |
| Omitting Long, et al. 2021 | 17.9% | 1.241 (1.054, 1.463) | 0.0098 |
| Omitting Shaharir, et al. 2021 | 25.7% | 1.192 (1.017, 1.397) | 0.0303 |
| Omitting Dogan, et al. 2020 | 25.3% | 1.194 (1.023, 1.394) | 0.0245 |
| Omitting Hisada, et al. 2018 | 24.4% | 1.198 (1.026, 1.399) | 0.0222 |
| Omitting Tse, et al. 2016 | 17.5% | 1.159 (0.991, 1.355) | 0.0653 |
| Omitting Al Saleh, et al. 2010 | 23.8% | 1.199 (1.027, 1.399) | 0.0216 |
| Omitting Griffiths, et al. 1979 | 25.2% | 1.194 (1.023, 1.393) | 0.0245 |
| Omitting Kunyakham, et al. 2012 | 0.0% | 1.123 (0.957, 1.317) | 0.1542 |
| Omitting Wu, et al. 2014 | 25.7% | 1.189 (1.019, 1.388) | 0.0279 |
| Omitting Li, et al. 2021 | 22.9% | 1.163 (0.990, 1.366) | 0.0657 |
| Omitting Lei, et al. 2024 | 25.2% | 1.183 (1.012, 1.383) | 0.0351 |
| Omitting Kwon, et al. 2018 | 25.3% | 1.197 (1.023, 1.400) | 0.0247 |
| Omitting Xu, et al. 2024 | 20.1% | 1.228 (1.045, 1.442) | 0.0126 |
| Omitting Chen, et al. 2021 | 25.6% | 1.192 (1.018, 1.396) | 0.0292 |
| Omitting AHSMU. 2023 | 4.6% | 1.267 (1.076, 1.491) | 0.0045 |
| Omitting WCHSCU. 2020 | 25.2% | 1.193 (1.023, 1.391) | 0.0247 |
| Omitting MHMU. 2023 | 24.5% | 1.194 (1.024, 1.393) | 0.0236 |
| Before omitting | 21.3% | 1.190 (1.021, 1.388) | 0.0261 |

CI: confidence interval; AHSMU: Affiliated Hospital of Southwest Medical University; WCHSCU: West China Hospital of Sichuan University; MHMU: Minda Hospital of Hubei Minzu University.
