# Supplementary material for: Meta-analysis of factors for osteonecrosis in systemic lupus erythematosus: integration of comprehensive literatures and multicenter databases
Source: Front Immunol. 2026 Jul 2;17:1679237. doi: 10.3389/fimmu.2026.1679237 (PMC13372907; doi:10.3389/fimmu.2026.1679237)
Supplement: Supplementary file 1 [file DataSheet1.zip › Supplementary Material/Supplementary table 22.docx]

Supplementary table 22 Sensitivity analysis for Reynaud’s phenomenon in the meta-analysis.

| Sensitivity analysis | Heterogeneity (I^2^) | Combined effect size (95% CI) | P value |
| --- | --- | --- | --- |
| Omitting Xiong, et al. 2022 | 50.1% | 1.457 (1.272, 1.669) | <0.0001 |
| Omitting Dogan, et al. 2020 | 50.0% | 1.454 (1.269, 1.666) | <0.0001 |
| Omitting Tse, et al. 2016 | 50.8% | 1.479 (1.289, 1.698) | <0.0001 |
| Omitting Jokar, et al. 2016 | 50.9% | 1.464 (1.278, 1.678) | <0.0001 |
| Omitting Sheikh, et al. 1998 | 50.8% | 1.473 (1.286, 1.687) | <0.0001 |
| Omitting Watanabe, et al. 1997 | 50.9% | 1.470 (1.283, 1.683) | <0.0001 |
| Omitting Mok, et al. 1998 | 50.4% | 1.485 (1.295, 1.704) | <0.0001 |
| Omitting Al Saleh, et al. 2010 | 50.7% | 1.464 (1.278, 1.676) | <0.0001 |
| Omitting Massardo, et al. 1992 | 50.1% | 1.455 (1.270, 1.667) | <0.0001 |
| Omitting Yang, et al. 2015 | 50.9% | 1.472 (1.284, 1.689) | <0.0001 |
| Omitting Ono, et al. 1992 | 50.9% | 1.468 (1.282, 1.681) | <0.0001 |
| Omitting Griffiths, et al. 1979 | 50.8% | 1.474 (1.287, 1.688) | <0.0001 |
| Omitting Calvo-Alen, et al. 2006 | 50.3% | 1.486 (1.296, 1.704) | <0.0001 |
| Omitting Hamijoyo, et al. 2008 | 50.9% | 1.467 (1.280, 1.682) | <0.0001 |
| Omitting Nagasawa, et al. 1989 | 50.9% | 1.474 (1.286, 1.690) | <0.0001 |
| Omitting Weiner, et al. 1989 | 49.5% | 1.483 (1.295, 1.699) | <0.0001 |
| Omitting Lee, et al. 2013 | 50.4% | 1.450 (1.263, 1.664) | <0.0001 |
| Omitting Faezi, et al. 2014 | 50.5% | 1.489 (1.295, 1.711) | <0.0001 |
| Omitting Fialho, et al. 2007 | 48.5% | 1.490 (1.301, 1.707) | <0.0001 |
| Omitting Sayarlioglu, et al. 2010 | 50.2% | 1.444 (1.258, 1.659) | <0.0001 |
| Omitting Prasad, et al. 2007 | 50.8% | 1.480 (1.289, 1.699) | <0.0001 |
| Omitting Zizic, et al. 1985 | 50.9% | 1.466 (1.279, 1.680) | <0.0001 |
| Omitting Gladman, et al. 2001 | 48.7% | 1.513 (1.317, 1.736) | <0.0001 |
| Omitting Smith, et al. 1976 | 50.0% | 1.459 (1.275, 1.671) | <0.0001 |
| Omitting Li, et al. 2008 | 47.4% | 1.437 (1.254, 1.647) | <0.0001 |
| Omitting Qi, et al. 2010 | 50.2% | 1.450 (1.264, 1.663) | <0.0001 |
| Omitting Xuan, et al. 2011 | 49.5% | 1.496 (1.304, 1.715) | <0.0001 |
| Omitting Shen, et al. 2012 | 50.8% | 1.473 (1.286, 1.687) | <0.0001 |
| Omitting Shi, et al. 2013 | 46.4% | 1.428 (1.246, 1.637) | <0.0001 |
| Omitting Wu, et al. 2014 | 50.6% | 1.459 (1.275, 1.672) | <0.0001 |
| Omitting Lin, et al. 2014 | 49.0% | 1.445 (1.261, 1.656) | <0.0001 |
| Omitting Wang, et al. 2018 | 46.8% | 1.432 (1.250, 1.642) | <0.0001 |
| Omitting Li, et al. 2021 | 50.9% | 1.473 (1.282, 1.694) | <0.0001 |
| Omitting Lei, et al. 2024 | 50.0% | 1.489 (1.299, 1.708) | <0.0001 |
| Omitting Zhang, et al. 2008 | 43.5% | 1.416 (1.234, 1.623) | <0.0001 |
| Omitting Liu, et al. 2011 | 49.5% | 1.445 (1.260, 1.656) | <0.0001 |
| Omitting Li, et al. 2014 | 50.9% | 1.467 (1.281, 1.681) | <0.0001 |
| Omitting Tang, et al. 1999 | 50.9% | 1.473 (1.285, 1.688) | <0.0001 |
| Omitting Shen, et al. 2005 | 48.5% | 1.443 (1.258, 1.654) | <0.0001 |
| Omitting Gladman, et al. 2018 | 45.2% | 1.579 (1.370, 1.820) | <0.0001 |
| Omitting Xu, et al. 2024 | 50.9% | 1.479 (1.286, 1.700) | <0.0001 |
| Omitting Wang, et al. 2009 | 50.5% | 1.481 (1.292, 1.698) | <0.0001 |
| Omitting AHSMU. 2023 | 50.9% | 1.470 (1.284, 1.683) | <0.0001 |
| Omitting WCHSCU. 2020 | 46.4% | 1.459 (1.275, 1.671) | <0.0001 |
| Omitting MHMU. 2023 | 50.9% | 1.467 (1.282, 1.680) | <0.0001 |
| Before omitting | 49.8% | 1.468 (1.282, 1.680) | <0.0001 |

CI: confidence interval; AHSMU: Affiliated Hospital of Southwest Medical University; WCHSCU: West China Hospital of Sichuan University; MHMU: Minda Hospital of Hubei Minzu University.
