# Supplementary material for: Meta-analysis of factors for osteonecrosis in systemic lupus erythematosus: integration of comprehensive literatures and multicenter databases
Source: Front Immunol. 2026 Jul 2;17:1679237. doi: 10.3389/fimmu.2026.1679237 (PMC13372907; doi:10.3389/fimmu.2026.1679237)
Supplement: Supplementary file 1 [file DataSheet1.zip › Supplementary Material/Supplementary table 23.docx]

Supplementary table 23 Sensitivity analysis for Cushingoid in the meta-analysis.

| Sensitivity analysis | Heterogeneity (I^2^) | Combined effect size (95% CI) | P value |
| --- | --- | --- | --- |
| Omitting Dogan, et al. 2020 | 44.3% | 3.288 (2.493, 4.337) | <0.0001 |
| Omitting Tse, et al. 2016 | 48.4% | 3.401 (2.521, 4.588) | <0.0001 |
| Omitting Mok, et al. 1998 | 48.5% | 3.412 (2.562, 4.544) | <0.0001 |
| Omitting Massardo, et al. 1992 | 46.8% | 3.303 (2.501, 4.362) | <0.0001 |
| Omitting Hamijoyo, et al. 2008 | 29.0% | 3.957 (2.946, 5.315) | <0.0001 |
| Omitting Weiner, et al. 1989 | 47.5% | 3.433 (2.613, 4.510) | <0.0001 |
| Omitting Lee, et al. 2013 | 40.0% | 3.161 (2.398, 4.167) | <0.0001 |
| Omitting Fialho, et al. 2007 | 48.4% | 3.389 (2.581, 4.451) | <0.0001 |
| Omitting Prasad, et al. 2007 | 45.0% | 3.593 (2.705, 4.773) | <0.0001 |
| Omitting Zizic, et al. 1985 | 16.9% | 3.106 (2.350, 4.105) | <0.0001 |
| Omitting Gladman, et al. 2001 | 48.3% | 3.379 (2.532, 4.509) | <0.0001 |
| Omitting Mont, et al. 1997 | 48.0% | 3.348 (2.521, 4.446) | <0.0001 |
| Omitting Tang, et al. 1999 | 45.4% | 3.563 (2.687, 4.723) | <0.0001 |
| Omitting AHSMU. 2023 | 48.1% | 3.393 (2.585, 4.454) | <0.0001 |
| Omitting MHMU. 2023 | 48.3% | 3.415 (2.597, 4.491) | <0.0001 |
| Before omitting | 44.5% | 3.401 (2.592, 4.461) | <0.0001 |

CI: confidence interval; AHSMU: Affiliated Hospital of Southwest Medical University; MHMU: Minda Hospital of Hubei Minzu University.
