# Supplementary material for: Meta-analysis of factors for osteonecrosis in systemic lupus erythematosus: integration of comprehensive literatures and multicenter databases
Source: Front Immunol. 2026 Jul 2;17:1679237. doi: 10.3389/fimmu.2026.1679237 (PMC13372907; doi:10.3389/fimmu.2026.1679237)
Supplement: Supplementary file 1 [file DataSheet1.zip › Supplementary Material/Supplementary table 24.docx]

Supplementary table 24 Sensitivity analysis for osteoporosis in the meta-analysis.

| Sensitivity analysis | Heterogeneity (I^2^) | Combined effect size (95% CI) | P value |
| --- | --- | --- | --- |
| Omitting Cheng, et al. 2023 | 82.6% | 1.947 (1.665, 2.277) | <0.0001 |
| Omitting Shaharir, et al. 2021 | 82.0% | 1.899 (1.619, 2.227) | <0.0001 |
| Omitting Dogan, et al. 2020 | 82.6% | 1.922 (1.643, 2.249) | <0.0001 |
| Omitting Joo, et al. 2014 | 81.4% | 2.224 (1.822, 2.715) | <0.0001 |
| Omitting Lee, et al. 2013 | 82.9% | 1.978 (1.690, 2.316) | <0.0001 |
| Omitting Faezi, et al. 2014 | 81.0% | 2.165 (1.833, 2.558) | <0.0001 |
| Omitting Prasad, et al. 2007 | 81.3% | 2.031 (1.736, 2.376) | <0.0001 |
| Omitting Liu, et al. 2022 | 81.4% | 1.896 (1.618, 2.221) | <0.0001 |
| Omitting Wu, et al. 2014 | 82.2% | 1.923 (1.643, 2.251) | <0.0001 |
| Omitting Li, et al. 2021 | 80.8% | 1.825 (1.554, 2.142) | <0.0001 |
| Omitting Li, et al. 2014 | 82.2% | 2.007 (1.715, 2.349) | <0.0001 |
| Omitting Xu, et al. 2024 | 82.5% | 1.906 (1.623, 2.238) | <0.0001 |
| Omitting Chen, et al. 2021 | 83.0% | 1.955 (1.670, 2.288) | <0.0001 |
| Omitting AHSMU. 2023 | 77.1% | 1.762 (1.492, 2.081) | <0.0001 |
| Omitting WCHSCU. 2020 | 82.4% | 2.003 (1.711, 2.344) | <0.0001 |
| Omitting MHMU. 2023 | 81.3% | 2.077 (1.768, 2.439) | <0.0001 |
| Before omitting | 81.8% | 1.961 (1.678, 2.291) | <0.0001 |

CI: confidence interval; AHSMU: Affiliated Hospital of Southwest Medical University; WCHSCU: West China Hospital of Sichuan University; MHMU: Minda Hospital of Hubei Minzu University.
