# Supplementary material for: Meta-analysis of factors for osteonecrosis in systemic lupus erythematosus: integration of comprehensive literatures and multicenter databases
Source: Front Immunol. 2026 Jul 2;17:1679237. doi: 10.3389/fimmu.2026.1679237 (PMC13372907; doi:10.3389/fimmu.2026.1679237)
Supplement: Supplementary file 1 [file DataSheet1.zip › Supplementary Material/Supplementary table 25.docx]

Supplementary table 25 Sensitivity analysis for thrombophlebitis in the meta-analysis.

| Sensitivity analysis | Heterogeneity (I^2^) | Combined effect size (95% CI) | P value |
| --- | --- | --- | --- |
| Omitting Weiner, et al. 1989 | 13.8% | 1.890 (1.015, 3.517) | 0.0446 |
| Omitting Sayarlioglu, et al. 2010 | 0.1% | 2.191 (1.061, 4.525) | 0.0341 |
| Omitting Gladman, et al. 2001 | 14.3% | 1.955 (0.987, 3.873) | 0.0545 |
| Omitting Mont, et al. 1997 | 0.0% | 1.470 (0.768, 2.815) | 0.2446 |
| Omitting Li, et al. 2021 | 14.5% | 1.930 (0.918, 4.057) | 0.0830 |
| Before omitting | 0.0% | 1.856 (1.012, 3.406) | 0.0457 |

CI: confidence interval.
