# Supplementary material for: Meta-analysis of factors for osteonecrosis in systemic lupus erythematosus: integration of comprehensive literatures and multicenter databases
Source: Front Immunol. 2026 Jul 2;17:1679237. doi: 10.3389/fimmu.2026.1679237 (PMC13372907; doi:10.3389/fimmu.2026.1679237)
Supplement: Supplementary file 1 [file DataSheet1.zip › Supplementary Material/Supplementary table 26.docx]

Supplementary table 26 Sensitivity analysis for proteinurial in the meta-analysis.

| Sensitivity analysis | Heterogeneity (I^2^) | Combined effect size (95% CI) | P value |
| --- | --- | --- | --- |
| Omitting Dogan, et al. 2020 | 36.7% | 1.321 (1.014, 1.721) | 0.0391 |
| Omitting Al Saleh, et al. 2010 | 43.2% | 1.322 (1.013, 1.724) | 0.0396 |
| Omitting Massardo, et al. 1992 | 46.1% | 1.336 (1.020, 1.748) | 0.0351 |
| Omitting Griffiths, et al. 1979 | 45.7% | 1.346 (1.033, 1.753) | 0.0276 |
| Omitting Sayarlioglu, et al. 2010 | 47.1% | 1.344 (1.012, 1.786) | 0.0413 |
| Omitting Smith, et al. 1976 | 47.3% | 1.364 (1.050, 1.774) | 0.0202 |
| Omitting Li, et al. 2008 | 47.4% | 1.375 (1.050, 1.801) | 0.0205 |
| Omitting Xuan, et al. 2011 | 38.5% | 1.452 (1.109, 1.901) | 0.0066 |
| Omitting Shen, et al. 2012 | 47.4% | 1.371 (1.053, 1.786) | 0.0193 |
| Omitting Lin, et al. 014 | 47.4% | 1.370 (1.048, 1.792) | 0.0212 |
| Omitting Wang, et al. 2018 | 24.0% | 1.240 (0.945, 1.627) | 0.1215 |
| Omitting Zhang, et al. 2008 | 45.7% | 1.352 (1.041, 1.757) | 0.0237 |
| Omitting AHSMU. 2023 | 14.6% | 1.787 (1.297, 2.461) | 0.0004 |
| Before omitting | 42.6% | 1.370 (1.056, 1.777) | 0.0178 |

CI: confidence interval; AHSMU: Affiliated Hospital of Southwest Medical University.
