# Supplementary material for: Meta-analysis of factors for osteonecrosis in systemic lupus erythematosus: integration of comprehensive literatures and multicenter databases
Source: Front Immunol. 2026 Jul 2;17:1679237. doi: 10.3389/fimmu.2026.1679237 (PMC13372907; doi:10.3389/fimmu.2026.1679237)
Supplement: Supplementary file 1 [file DataSheet1.zip › Supplementary Material/Supplementary table 27.docx]

Supplementary table 27 Sensitivity analysis for anti-SSA in the meta-analysis.

| Sensitivity analysis | Heterogeneity (I^2^) | Combined effect size (95% CI) | P value |
| --- | --- | --- | --- |
| Omitting Cheng, et al. 2023 | 4.6% | 0.753 (0.637, 0.890) | 0.0009 |
| Omitting Xiong, et al. 2022 | 21.7% | 0.809 (0.693, 0.945) | 0.0076 |
| Omitting Long, et al. 2021 | 21.9% | 0.795 (0.676, 0.937) | 0.0061 |
| Omitting Tse, et al. 2016 | 16.5% | 0.825 (0.704, 0.967) | 0.0176 |
| Omitting Sekiya, et al. 2009 | 22.4% | 0.806 (0.691, 0.940) | 0.0059 |
| Omitting Watanabe, et al. 1997 | 3.9% | 0.791 (0.678, 0.923) | 0.0029 |
| Omitting Mok, et al. 1998 | 22.5% | 0.804 (0.687, 0.940) | 0.0063 |
| Omitting Al Saleh, et al. 2010 | 18.4% | 0.813 (0.697, 0.949) | 0.0086 |
| Omitting Lee, et al. 2013 | 0.0% | 0.841 (0.719, 0.984) | 0.0304 |
| Omitting Wu, et al. 2014 | 20.8% | 0.799 (0.685, 0.933) | 0.0045 |
| Omitting Li, et al. 2021 | 18.0% | 0.825 (0.703, 0.967) | 0.0179 |
| Omitting Liu, et al. 2011 | 20.7% | 0.801 (0.687, 0.934) | 0.0047 |
| Omitting Li, et al. 2014 | 22.2% | 0.808 (0.692, 0.945) | 0.0075 |
| Omitting Vílchez-Oya, et al. 2019 | 2.4% | 0.806 (0.691, 0.939) | 0.0058 |
| Omitting Kwon, et al. 2018 | 2.5% | 0.807 (0.685, 0.952) | 0.0110 |
| Omitting Xu, et al. 2024 | 19.0% | 0.823 (0.702, 0.964) | 0.0159 |
| Omitting AHSMU. 2023 | 19.1% | 0.784 (0.667, 0.923) | 0.0034 |
| Omitting WCHSCU. 2020 | 20.5% | 0.795 (0.680, 0.930) | 0.0042 |
| Before omitting | 17.7% | 0.805 (0.691, 0.938) | 0.0055 |

Anti-SSA: anti-Sjogren Syndrome A antibody; CI: confidence interval; AHSMU: Affiliated Hospital of Southwest Medical University; WCHSCU: West China Hospital of Sichuan University.
