# Supplementary material for: Meta-analysis of factors for osteonecrosis in systemic lupus erythematosus: integration of comprehensive literatures and multicenter databases
Source: Front Immunol. 2026 Jul 2;17:1679237. doi: 10.3389/fimmu.2026.1679237 (PMC13372907; doi:10.3389/fimmu.2026.1679237)
Supplement: Supplementary file 1 [file DataSheet1.zip › Supplementary Material/Supplementary table 28.docx]

Supplementary table 28 Sensitivity analysis for anti-SSB in the meta-analysis.

| Sensitivity analysis | Heterogeneity (I^2^) | Combined effect size (95% CI) | P value |
| --- | --- | --- | --- |
| Omitting Cheng, et al. 2023 | 0.0% | 0.724 (0.580, 0.904) | 0.0043 |
| Omitting Xiong, et al. 2022 | 4.5% | 0.770 (0.627, 0.946) | 0.0130 |
| Omitting Long, et al. 2021 | 6.4% | 0.766 (0.618, 0.950) | 0.0150 |
| Omitting Tse, et al. 2016 | 0.0% | 0.811 (0.658, 1.000) | 0.0490 |
| Omitting Watanabe, et al. 1997 | 0.6% | 0.764 (0.623, 0.937) | 0.0098 |
| Omitting Mok, et al. 1998 | 0.0% | 0.735 (0.597, 0.906) | 0.0038 |
| Omitting Al Saleh, et al. 2010 | 0.6% | 0.763 (0.621, 0.937) | 0.0099 |
| Omitting Lee, et al. 2013 | 0.0% | 0.787 (0.637, 0.971) | 0.0254 |
| Omitting Xuan, et al. 2011 | 6.6% | 0.763 (0.619, 0.940) | 0.0111 |
| Omitting Li, et al. 2021 | 5.1% | 0.774 (0.625, 0.958) | 0.0184 |
| Omitting Liu, et al. 2011 | 0.3% | 0.762 (0.621, 0.935) | 0.0092 |
| Omitting Li, et al. 2014 | 0.0% | 0.782 (0.636, 0.963) | 0.0205 |
| Omitting Kwon, et al. 2018 | 6.6% | 0.764 (0.619, 0.943) | 0.0121 |
| Omitting Xu, et al. 2024 | 5.8% | 0.772 (0.619, 0.963) | 0.0216 |
| Omitting AHSMU. 2023 | 4.7% | 0.749 (0.605, 0.926) | 0.0077 |
| Omitting WCHSCU. 2020 | 0.0% | 0.736 (0.596, 0.908) | 0.0042 |
| Before omitting | 0.0% | 0.764 (0.623, 0.936) | 0.0095 |

Anti-SSB: anti-Sjogren Syndrome B antibody; CI: confidence interval; AHSMU: Affiliated Hospital of Southwest Medical University; WCHSCU: West China Hospital of Sichuan University.
