# Supplementary material for: Meta-analysis of factors for osteonecrosis in systemic lupus erythematosus: integration of comprehensive literatures and multicenter databases
Source: Front Immunol. 2026 Jul 2;17:1679237. doi: 10.3389/fimmu.2026.1679237 (PMC13372907; doi:10.3389/fimmu.2026.1679237)
Supplement: Supplementary file 1 [file DataSheet1.zip › Supplementary Material/Supplementary table 29.docx]

Supplementary table 29 Sensitivity analysis for antiphospholipid antibody in the meta-analysis.

| Sensitivity analysis | Heterogeneity (I^2^) | Combined effect size (95% CI) | P value |
| --- | --- | --- | --- |
| Omitting Xiong, et al. 2022 | 57.1% | 1.148 (0.935, 1.410) | 0.1884 |
| Omitting Long, et al. 2021 | 46.6% | 1.325 (1.065, 1.649) | 0.0115 |
| Omitting Dogan, et al. 2020 | 56.2% | 1.148 (0.935, 1.409) | 0.1870 |
| Omitting Hisada, et al. 2018 | 53.2% | 1.117 (0.907, 1.376) | 0.2985 |
| Omitting Tse, et al. 2016 | 59.8% | 1.157 (0.934, 1.432) | 0.1825 |
| Omitting Kuroda, et al. 2015 | 57.6% | 1.207 (0.983, 1.482) | 0.0720 |
| Omitting Nagasawa, et al. 2005 | 58.0% | 1.161 (0.947, 1.423) | 0.1522 |
| Omitting Li, et al. 2014 | 59.5% | 1.167 (0.952, 1.431) | 0.1366 |
| Omitting Tang, et al. 1999 | 59.2% | 1.167 (0.953, 1.431) | 0.1359 |
| Omitting Shen, et al. 2005 | 58.7% | 1.151 (0.935, 1.416) | 0.1854 |
| Omitting Gladman, et al. 2018 | 57.0% | 1.248 (1.002, 1.554) | 0.0477 |
| Omitting Kwon, et al. 2018 | 55.9% | 1.286 (1.019, 1.623) | 0.0339 |
| Omitting Chen, et al. 2021 | 50.8% | 1.103 (0.893, 1.363) | 0.3624 |
| Before omitting | 56.5% | 1.179 (0.963, 1.442) | 0.1106 |

CI: confidence interval.
