# Supplementary material for: Meta-analysis of factors for osteonecrosis in systemic lupus erythematosus: integration of comprehensive literatures and multicenter databases
Source: Front Immunol. 2026 Jul 2;17:1679237. doi: 10.3389/fimmu.2026.1679237 (PMC13372907; doi:10.3389/fimmu.2026.1679237)
Supplement: Supplementary file 1 [file DataSheet1.zip › Supplementary Material/Supplementary table 3.docx]

Supplementary table 3 General information of the patients from WCHSCU cohort.

| Variable | Total (n=2295) | SLE-ON (n=53) | SLE-non-ON (n=2242) | P value |
| --- | --- | --- | --- | --- |
| Demographic and clinical characteristics |  |  |  |  |
| Female, N (%) | 2012 (87.67) | 44 (83.02) | 1968 (87.78) | 0.298 |
| Age, year | 37.86±13.26 | 38.61±12.09 | 37.84±13.28 | 0.686 |
| Arthritis (+), N (%) | 2170 (94.55) | 3 (5.66) | 75 (3.35) | 0.592 |
| Hypertension (+), N (%) | 761 (33.16) | 8 (15.09) | 753 (33.59) | 0.005 |
| Diabetes mellitus (+), N (%) | 98 (4.27) | 0 (0.00) | 98 (4.37) | 0.226 |
| Nephritis (+), N (%) | 1479 (64.44) | 34 (64.15) | 1445 (64.45) | 0.964 |
| Anemia (+), N (%) | 425 (18.52) | 8 (15.09) | 417 (18.60) | 0.516 |
| Cataract (+), N (%) | 21 (0.92) | 1 (1.89) | 20 (0.89) | 0.982 |
| Pulmonary arterial hypertension (+), N (%) | 103 (4.49) | 0 (0.00) | 103 (4.59) | 0.207 |
| Hematologic involvement (+), N (%) | 30 (1.31) | 0 (0.00) | 30 (1.34) | 0.813 |
| Reynaud’s phenomenon (+), N (%) | 1 (0.04) | 1 (1.89) | 0 (0.00) | <0.001 |
| Osteoporosis (+), N (%) | 137 (5.97) | 2 (3.77) | 135 (6.02) | 0.697 |
| Antiphospholipid syndrome (+), N (%) | 134 (5.84) | 1 (1.89) | 133 (5.93) | 0.345 |
| Sjögren’s syndrome (+), N (%) | 216 (9.41) | 2 (3.77) | 214 (9.55) | 0.236 |
| Laboratory characteristics |  |  |  |  |
| ANA (+), N (%) | 2283 (99.48) | 53 (100.00) | 2230 (99.46) | 1.000 |
| Anti-dsDNA (+), N (%) | 153 (6.67) | 6 (11.32) | 147 (6.56) | 0.273 |
| Anti-Sm (+), N (%) | 478 (20.84) | 16 (30.19) | 462 (20.61) | 0.090 |
| Anti-SSA (+), N (%) | 1930 (84.10) | 45 (84.91) | 1885 (84.08) | 0.870 |
| Anti-SSB (+), N (%) | 214 (9.32) | 7 (13.21) | 207 (9.23) | 0.457 |
| Anti-RNP (+), N (%) | 650 (28.32) | 14 (26.42) | 636 (28.37) | 0.755 |
| Lupus anticoagulant (+), N (%) | 1152 (50.20) | 28 (52.83) | 1124 (50.13) | 0.698 |
| Leukopenia (+), N (%) | 122 (5.32) | 4 (7.55) | 118 (5.26) | 0.672 |
| Thrombocytopenia (+), N (%) | 113 (4.92) | 3 (5.66) | 110 (4.91) | 1.000 |
| RF (+), N (%) | 2295 (100.00) | 53 (100.00) | 2242 (100.00) | 1.000 |

WCHSCU: West China Hospital of Sichuan University; SLE: systemic lupus erythematosus; ON: osteonecrosis; SD: standard deviation; ANA: antinuclear antibody; Anti-dsDNA: anti-double stranded DNA antibody; Anti-Sm: anti-smith antibody; Anti-SSA: anti-Sjogren Syndrome A antibody; Anti-SSB: anti-Sjogren Syndrome B antibody; Anti-RNP: anti-ribonucleoprotein antibody; RF: rheumatoid factor.
