# Supplementary material for: Meta-analysis of factors for osteonecrosis in systemic lupus erythematosus: integration of comprehensive literatures and multicenter databases
Source: Front Immunol. 2026 Jul 2;17:1679237. doi: 10.3389/fimmu.2026.1679237 (PMC13372907; doi:10.3389/fimmu.2026.1679237)
Supplement: Supplementary file 1 [file DataSheet1.zip › Supplementary Material/Supplementary table 30.docx]

Supplementary table 30 Sensitivity analysis for ACL in the meta-analysis.

| Sensitivity analysis | Heterogeneity (I^2^) | Combined effect size (95% CI) | P value |
| --- | --- | --- | --- |
| Omitting Xiong, et al. 2022 | 60.6% | 1.381 (1.094, 1.743) | 0.0066 |
| Omitting Long, et al. 2021 | 50.9% | 1.572 (1.230, 2.009) | 0.0003 |
| Omitting Dogan, et al. 2020 | 60.5% | 1.357 (1.073, 1.716) | 0.0107 |
| Omitting Watanabe, et al. 1997 | 59.4% | 1.354 (1.072, 1.708) | 0.0108 |
| Omitting Yang, et al. 2015 | 59.7% | 1.426 (1.119, 1.818) | 0.0041 |
| Omitting Faezi, et al. 2014 | 57.9% | 1.483 (1.155, 1.903) | 0.0020 |
| Omitting Gladman, et al. 2001 | 60.8% | 1.373 (1.087, 1.734) | 0.0079 |
| Omitting Li, et al. 2008 | 56.4% | 1.302 (1.028, 1.650) | 0.0288 |
| Omitting Xuan, et al. 2011 | 60.1% | 1.405 (1.107, 1.782) | 0.0051 |
| Omitting Shen, et al. 2012 | 59.1% | 1.339 (1.060, 1.693) | 0.0145 |
| Omitting Shi, et al. 2013 | 48.1% | 1.240 (0.976, 1.575) | 0.0781 |
| Omitting Wu, et al. 2014 | 58.6% | 1.345 (1.065, 1.698) | 0.0127 |
| Omitting Wang, et al. 2018 | 59.8% | 1.411 (1.112, 1.791) | 0.0046 |
| Omitting Li, et al. 2021 | 58.7% | 1.454 (1.139, 1.856) | 0.0027 |
| Omitting Liu, et al. 2011 | 56.9% | 1.303 (1.028, 1.652) | 0.0285 |
| Omitting Chen, et al. 2021 | 56.1% | 1.285 (1.008, 1.638) | 0.0433 |
| Omitting Wang, et al. 2009 | 60.7% | 1.363 (1.076, 1.725) | 0.0101 |
| Before omitting | 58.1% | 1.372 (1.089, 1.729) | 0.0073 |

ACL: anticardiolipin antibody; CI: confidence interval.
