# Supplementary material for: Meta-analysis of factors for osteonecrosis in systemic lupus erythematosus: integration of comprehensive literatures and multicenter databases
Source: Front Immunol. 2026 Jul 2;17:1679237. doi: 10.3389/fimmu.2026.1679237 (PMC13372907; doi:10.3389/fimmu.2026.1679237)
Supplement: Supplementary file 1 [file DataSheet1.zip › Supplementary Material/Supplementary table 31.docx]

Supplementary table 31 Sensitivity analysis for elevated ESR in the meta-analysis.

| Sensitivity analysis | Heterogeneity (I^2^) | Combined effect size (95% CI) | P value |
| --- | --- | --- | --- |
| Omitting Long, et al. 2021 | 0.0% | 1.345 (0.713, 2.537) | 0.3598 |
| Omitting Jokar, et al. 2016 | 0.0% | 1.655 (1.062, 2.579) | 0.0260 |
| Omitting Wu, et al. 2014 | 0.0% | 1.738 (1.154, 2.616) | 0.0081 |
| Before omitting | 0.0% | 1.623 (1.104, 2.387) | 0.0138 |

ESR: erythrocytes sedimentation rate; CI: confidence interval.
