# Supplementary material for: Meta-analysis of factors for osteonecrosis in systemic lupus erythematosus: integration of comprehensive literatures and multicenter databases
Source: Front Immunol. 2026 Jul 2;17:1679237. doi: 10.3389/fimmu.2026.1679237 (PMC13372907; doi:10.3389/fimmu.2026.1679237)
Supplement: Supplementary file 1 [file DataSheet1.zip › Supplementary Material/Supplementary table 32.docx]

Supplementary table 32 Sensitivity analysis for leukopenia in the meta-analysis.

| Sensitivity analysis | Heterogeneity (I^2^) | Combined effect size (95% CI) | P value |
| --- | --- | --- | --- |
| Omitting Jokar, et al. 2016 | 22.3% | 1.278 (1.013, 1.612) | 0.0388 |
| Omitting Watanabe, et al. 1997 | 23.7% | 1.305 (1.043, 1.633) | 0.0199 |
| Omitting Mok, et al. 1998 | 23.6% | 1.332 (1.055, 1.681) | 0.0160 |
| Omitting Al Saleh, et al. 2010 | 24.1% | 1.323 (1.056, 1.658) | 0.0150 |
| Omitting Massardo, et al. 1992 | 24.6% | 1.316 (1.048, 1.652) | 0.0182 |
| Omitting Lee, et al. 2013 | 12.7% | 1.242 (0.984, 1.567) | 0.0676 |
| Omitting Faezi, et al. 2014 | 13.2% | 1.429 (1.105, 1.849) | 0.0066 |
| Omitting Sayarlioglu, et al. 2010 | 24.3% | 1.293 (1.021, 1.637) | 0.0327 |
| Omitting Zizic, et al. 1985 | 24.1% | 1.324 (1.055, 1.661) | 0.0154 |
| Omitting Smith, et al. 1976 | 0.0% | 1.280 (1.023, 1.601) | 0.0307 |
| Omitting Xuan, et al. 2011 | 13.7% | 1.250 (0.992, 1.574) | 0.0583 |
| Omitting Vílchez-Oya, et al. 2019 | 22.6% | 1.327 (1.062, 1.660) | 0.0130 |
| Omitting AHSMU. 2023 | 18.3% | 1.346 (1.075, 1.685) | 0.0096 |
| Omitting WCHSCU. 2020 | 24.6% | 1.311 (1.045, 1.645) | 0.0195 |
| Omitting MHMU. 2023 | 1.6% | 1.429 (1.137, 1.794) | 0.0022 |
| Before omitting | 18.8% | 1.317 (1.055, 1.644) | 0.0151 |

CI: confidence interval; AHSMU: Affiliated Hospital of Southwest Medical University; WCHSCU: West China Hospital of Sichuan University; MHMU: Minda Hospital of Hubei Minzu University.
