# Supplementary material for: Meta-analysis of factors for osteonecrosis in systemic lupus erythematosus: integration of comprehensive literatures and multicenter databases
Source: Front Immunol. 2026 Jul 2;17:1679237. doi: 10.3389/fimmu.2026.1679237 (PMC13372907; doi:10.3389/fimmu.2026.1679237)
Supplement: Supplementary file 1 [file DataSheet1.zip › Supplementary Material/Supplementary table 33.docx]

Supplementary table 33 Sensitivity analysis for steroid pulse therapy in the meta-analysis.

| Sensitivity analysis | Heterogeneity (I^2^) | Combined effect size (95% CI) | P value |
| --- | --- | --- | --- |
| Omitting Long, et al. 2021 | 39.1% | 1.761 (1.473, 2.106) | <0.0001 |
| Omitting Dogan, et al. 2020 | 38.0% | 1.785 (1.493, 2.134) | <0.0001 |
| Omitting Hisada, et al. 2018 | 39.1% | 1.767 (1.475, 2.117) | <0.0001 |
| Omitting Kuroda, et al. 2015 | 34.6% | 1.806 (1.511, 2.159) | <0.0001 |
| Omitting Sekiya, et al. 2009 | 35.4% | 1.789 (1.498, 2.137) | <0.0001 |
| Omitting Mok, et al. 1998 | 37.4% | 1.794 (1.499, 2.146) | <0.0001 |
| Omitting Massardo, et al. 1992 | 36.8% | 1.734 (1.450, 2.075) | <0.0001 |
| Omitting Nagasawa, et al. 2005 | 31.5% | 1.712 (1.432, 2.047) | <0.0001 |
| Omitting Oinuma, et al. 2001 | 39.1% | 1.765 (1.475, 2.114) | <0.0001 |
| Omitting Nagasawa, et al. 1989 | 38.1% | 1.783 (1.492, 2.132) | <0.0001 |
| Omitting Lee, et al. 2013 | 38.3% | 1.791 (1.495, 2.146) | <0.0001 |
| Omitting Sayarlioglu, et al. 2010 | 38.3% | 1.798 (1.496, 2.160) | <0.0001 |
| Omitting Prasad, et al. 2007 | 37.2% | 1.800 (1.504, 2.154) | <0.0001 |
| Omitting Li, et al. 2008 | 37.0% | 1.729 (1.445, 2.069) | <0.0001 |
| Omitting Xuan, et al. 2011 | 38.7% | 1.747 (1.460, 2.091) | <0.0001 |
| Omitting Shen, et al. 2012 | 37.5% | 1.784 (1.493, 2.132) | <0.0001 |
| Omitting Shi, et al. 2013 | 36.3% | 1.720 (1.436, 2.060) | <0.0001 |
| Omitting Wu, et al. 2014 | 38.6% | 1.751 (1.464, 2.093) | <0.0001 |
| Omitting Lin, et al. 2014 | 36.4% | 1.732 (1.449, 2.071) | <0.0001 |
| Omitting Wang,MC 2018 | 31.1% | 1.699 (1.419, 2.034) | <0.0001 |
| Omitting Li, et al. 2021 | 30.4% | 1.901 (1.578, 2.291) | <0.0001 |
| Omitting Lei, et al. 2024 | 38.8% | 1.748 (1.460, 2.093) | <0.0001 |
| Omitting Li, et al. 2014 | 38.8% | 1.754 (1.468, 2.096) | <0.0001 |
| Omitting Shen, et al. 2005 | 36.5% | 1.742 (1.457, 2.082) | <0.0001 |
| Omitting Vílchez-Oya, et al. 2019 | 33.8% | 1.739 (1.456, 2.077) | <0.0001 |
| Omitting Kwon, et al. 2018 | 37.5% | 1.711 (1.419, 2.064) | <0.0001 |
| Omitting Xu, et al. 2024 | 39.1% | 1.763 (1.463, 2.125) | <0.0001 |
| Omitting Wang, et al. 2009 | 34.8% | 1.820 (1.520, 2.179) | <0.0001 |
| Before omitting | 36.8% | 1.764 (1.478, 2.106) | <0.0001 |

CI: confidence interval.
