# Supplementary material for: Meta-analysis of factors for osteonecrosis in systemic lupus erythematosus: integration of comprehensive literatures and multicenter databases
Source: Front Immunol. 2026 Jul 2;17:1679237. doi: 10.3389/fimmu.2026.1679237 (PMC13372907; doi:10.3389/fimmu.2026.1679237)
Supplement: Supplementary file 1 [file DataSheet1.zip › Supplementary Material/Supplementary table 34.docx]

Supplementary table 34 Sensitivity analysis for cyclophosphamide in the meta-analysis.

| Sensitivity analysis | Heterogeneity (I^2^) | Combined effect size (95% CI) | P value |
| --- | --- | --- | --- |
| Omitting Abdelkawy, et al. 2022 | 80.4% | 1.724 (1.437, 2.068) | <0.0001 |
| Omitting Xiong, et al. 2022 | 80.7% | 1.779 (1.492, 2.121) | <0.0001 |
| Omitting Long, et al. 2021 | 79.2% | 1.893 (1.573, 2.278) | <0.0001 |
| Omitting Shaharir, et al. 2021 | 76.4% | 1.962 (1.635, 2.355) | <0.0001 |
| Omitting Mok, et al. 1998 | 79.4% | 1.715 (1.436, 2.048) | <0.0001 |
| Omitting Al Saleh, et al. 2010 | 78.8% | 1.727 (1.450, 2.058) | <0.0001 |
| Omitting Lee, et al. 2013 | 79.2% | 1.698 (1.421, 2.028) | <0.0001 |
| Omitting Faezi, et al. 2014 | 71.1% | 2.055 (1.711, 2.467) | <0.0001 |
| Omitting Uea-areewongsa, et al. 2009 | 80.7% | 1.776 (1.491, 2.115) | <0.0001 |
| Omitting Lin, et al. 2014 | 80.6% | 1.788 (1.501, 2.131) | <0.0001 |
| Omitting Wang, et al. 2018 | 80.3% | 1.814 (1.520, 2.165) | <0.0001 |
| Omitting Li, et al. 2014 | 80.6% | 1.791 (1.502, 2.134) | <0.0001 |
| Omitting Kwon, et al. 2018 | 76.1% | 1.558 (1.287, 1.886) | <0.0001 |
| Omitting Xu, et al. 2024 | 79.1% | 1.656 (1.375, 1.994) | <0.0001 |
| Before omitting | 79.1% | 1.777 (1.495, 2.113) | <0.0001 |

CI: confidence interval.
