# Supplementary material for: Meta-analysis of factors for osteonecrosis in systemic lupus erythematosus: integration of comprehensive literatures and multicenter databases
Source: Front Immunol. 2026 Jul 2;17:1679237. doi: 10.3389/fimmu.2026.1679237 (PMC13372907; doi:10.3389/fimmu.2026.1679237)
Supplement: Supplementary file 1 [file DataSheet1.zip › Supplementary Material/Supplementary table 35.docx]

Supplementary table 35 Sensitivity analysis for age in the meta-analysis.

| Sensitivity analysis | Heterogeneity (I^2^) | Combined effect size (95% CI) | P value |
| --- | --- | --- | --- |
| Omitting Abdelkawy, et al. 2022 | 74.5% | -0.189 (-0.341, -0.037) | 0.0147 |
| Omitting Shaharir, et al. 2021 | 71.1% | -0.201 (-0.345, -0.057) | 0.0062 |
| Omitting Dogan, et al. 2020 | 75.4% | -0.171 (-0.323, -0.019) | 0.0271 |
| Omitting Hisada, et al. 2018 | 75.3% | -0.183 (-0.336, -0.030) | 0.0185 |
| Omitting Jokar, et al. 2016 | 74.6% | -0.175 (-0.323, -0.027) | 0.0203 |
| Omitting Sekiya, et al. 2009 | 75.4% | -0.172 (-0.322, -0.023) | 0.0244 |
| Omitting Migliaresi, et al. 1994 | 74.8% | -0.161 (-0.309, -0.013) | 0.0333 |
| Omitting Al Saleh, et al. 2010 | 75.1% | -0.187 (-0.337, -0.037) | 0.0144 |
| Omitting Massardo, et al. 1992 | 75.5% | -0.178 (-0.307, -0.025) | 0.0224 |
| Omitting Calvo-Alen, et al. 2006 | 75.1% | -0.167 (-0.319, -0.015) | 0.0317 |
| Omitting Nagasawa, et al. 2005 | 74.6% | -0.175 (-0.323, -0.027) | 0.0203 |
| Omitting Ghaleb, et al. 2011 | 73.9% | -0.197 (-0.342, -0.051) | 0.0079 |
| Omitting Oinuma, et al. 2001 | 75.4% | -0.181 (-0.334, -0.027) | 0.0199 |
| Omitting Sayarlioglu, et al. 2010 | 74.7% | -0.164 (-0.316, -0.012) | 0.0348 |
| Omitting Zizic, et al. 1985 | 75.5% | -0.175 (-0.327, -0.022) | 0.0249 |
| Omitting Uea-areewongsa, et al. 2009 | 75.4% | -0.182 (-0.334, -0.030) | 0.0182 |
| Omitting Gladman, et al. 2001 | 75.5% | -0.178 (-0.332, -0.024) | 0.0233 |
| Omitting Liu, et al. 2022 | 75.4% | -0.179 (-0.332, -0.026) | 0.0215 |
| Omitting Qi, et al. 2010 | 74.8% | -0.189 (-0.340, -0.038) | 0.0139 |
| Omitting Wang, et al. 2018 | 71.2% | -0.142 (-0.281, 0.005) | 0.0445 |
| Omitting Lei, et al. 2024 | 75.4% | -0.174 (-0.328, -0.020) | 0.0268 |
| Omitting Liu, et al. 2011 | 72.3% | -0.146 (-0.288, -0.004) | 0.0433 |
| Omitting Tang, et al. 1999 | 75.0% | -0.164 (-0.315, -0.013) | 0.0332 |
| Omitting Shen, et al. 2005 | 75.4% | -0.174 (-0.328, -0.020) | 0.0261 |
| Omitting Vílchez-Oya, et al. 2019 | 75.5% | -0.177 (-0.327, -0.027) | 0.0209 |
| Omitting Gladman, et al. 2018 | 75.3% | -0.182 (-0.336, -0.027) | 0.0210 |
| Omitting Kwon, et al. 2018 | 75.0% | -0.171 (-0.326, -0.015) | 0.0302 |
| Omitting Chen, et al. 2021 | 66.8% | -0.136 (-0.270, 0.002) | 0.0469 |
| Omitting AHSMU. 2023 | 73.9% | -0.190 (-0.342, -0.038) | 0.0145 |
| Omitting WCHSCU. 2020 | 75.0% | -0.186 (-0.339, -0.032) | 0.0175 |
| Omitting MHMU. 2023 | 75.4% | -0.175 (-0.330, -0.020) | 0.0270 |
| Before omitting | 74.6% | -0.175 (-0.323, -0.027) | 0.0203 |

CI: confidence interval; AHSMU: Affiliated Hospital of Southwest Medical University; WCHSCU: West China Hospital of Sichuan University; MHMU: Minda Hospital of Hubei Minzu University.
