# Supplementary material for: Meta-analysis of factors for osteonecrosis in systemic lupus erythematosus: integration of comprehensive literatures and multicenter databases
Source: Front Immunol. 2026 Jul 2;17:1679237. doi: 10.3389/fimmu.2026.1679237 (PMC13372907; doi:10.3389/fimmu.2026.1679237)
Supplement: Supplementary file 1 [file DataSheet1.zip › Supplementary Material/Supplementary table 36.docx]

Supplementary table 36 Sensitivity analysis for age at onset in the meta-analysis.

| Sensitivity analysis | Heterogeneity (I^2^) | Combined effect size (95% CI) | P value |
| --- | --- | --- | --- |
| Omitting Abdelkawy, et al. 2022 | 92.3% | -0.440 (-0.881, 0.001) | 0.0503 |
| Omitting Cheng, et al. 2023 | 91.0% | -0.405 (-0.843, 0.034) | 0.0704 |
| Omitting Xiong, et al. 2022 | 92.2% | -0.419 (-0.859, 0.021) | 0.0617 |
| Omitting Long, et al. 2021 | 92.3% | -0.436 (-0.877, 0.005) | 0.0528 |
| Omitting Shaharir, et al. 2021 | 92.1% | -0.452 (-0.890, -0.014) | 0.0432 |
| Omitting Dogan, et al. 2020 | 92.3% | -0.435 (-0.739, 0.005) | 0.0525 |
| Omitting Tse, et al. 2016 | 92.1% | -0.450 (-0.889, -0.118) | 0.0441 |
| Omitting Kuroda, et al. 2015 | 92.3% | -0.438 (-0.878, 0.002) | 0.0510 |
| Omitting Mok, et al. 1998 | 91.1% | -0.376 (-0.802, 0.049) | 0.0830 |
| Omitting Al Saleh, et al. 2010 | 86.8% | -0.246 (-0.459, -0.034) | 0.0230 |
| Omitting Yang, et al. 2015 | 92.2% | -0.449 (-0.887, -0.001) | 0.0450 |
| Omitting Ghaleb, et al. 2011 | 92.0% | -0.473 (-0.900, -0.045) | 0.0301 |
| Omitting Hamijoyo, et al. 2008 | 92.2% | -0.451 (-0.889, -0.013) | 0.0433 |
| Omitting Lee, et al. 2013 | 92.1% | -0.454 (-0.891, -0.017) | 0.0417 |
| Omitting Sayarlioglu, et al. 2010 | 92.3% | -0.429 (-0.870, 0.012) | 0.0567 |
| Omitting Prasad, et al. 2007 | 92.2% | -0.450 (-0.888, -0.015) | 0.0443 |
| Omitting Uea-areewongsa, et al. 2009 | 92.2% | -0.453 (-0.889, -0.017) | 0.0418 |
| Omitting Gladman, et al. 2001 | 92.3% | -0.441 (-0.881, -0.001) | 0.0494 |
| Omitting Kunyakham, et al. 2012 | 92.0% | -0.452 (-0.890, -0.014) | 0.0432 |
| Omitting Lei, et al. 2024 | 91.1% | -0.377 (-0.802, 0.049) | 0.0829 |
| Omitting Xu, et al. 2024 | 92.2% | -0.443 (-0.883, -0.003) | 0.0485 |
| Before omitting | 91.9% | -0.426 (-0.843, -0.009) | 0.0452 |

CI: confidence interval.
