# Supplementary material for: Meta-analysis of factors for osteonecrosis in systemic lupus erythematosus: integration of comprehensive literatures and multicenter databases
Source: Front Immunol. 2026 Jul 2;17:1679237. doi: 10.3389/fimmu.2026.1679237 (PMC13372907; doi:10.3389/fimmu.2026.1679237)
Supplement: Supplementary file 1 [file DataSheet1.zip › Supplementary Material/Supplementary table 37.docx]

Supplementary table 37 Sensitivity analysis for SLEDAI in the meta-analysis.

| Sensitivity analysis | Heterogeneity (I^2^) | Combined effect size (95% CI) | P value |
| --- | --- | --- | --- |
| Omitting Abdelkawy, et al. 2022 | 98.7% | 0.999 (0.015, 1.985) | 0.0466 |
| Omitting Cheng, et al. 2023 | 98.5% | 1.039 (0.063, 2.015) | 0.0369 |
| Omitting Long, et al. 2021 | 98.7% | 1.006 (0.022, 1.990) | 0.0450 |
| Omitting Dogan, et al. 2020 | 98.7% | 0.995 (0.010, 1.980) | 0.0477 |
| Omitting Hisada, et al. 2018 | 98.7% | 0.996 (0.011, 1.981) | 0.0475 |
| Omitting Kuroda, et al. 2015 | 98.7% | 1.009 (0.026, 1.992) | 0.0443 |
| Omitting Sekiya, et al. 2009 | 98.7% | 0.946 (-0.038, 1.930) | 0.0596 |
| Omitting Mok, et al. 1998 | 98.6% | 0.881 (-0.090, 1.852) | 0.0755 |
| Omitting Al Saleh, et al. 2010 | 98.6% | 0.812 (-0.122, 1.746) | 0.0884 |
| Omitting Calvo-Alen, et al. 2006 | 98.7% | 1.013 (0.031, 1.996) | 0.0432 |
| Omitting Ghaleb, et al. 2011 | 98.7% | 1.026 (0.047, 2.005) | 0.0400 |
| Omitting Oinuma, et al. 2001 | 98.7% | 0.999 (0.014, 1.984) | 0.0468 |
| Omitting Prasad, et al. 2007 | 98.7% | 1.001 (0.017, 1.986) | 0.0463 |
| Omitting Uea-areewongsa, et al. 2009 | 98.7% | 0.983 (-0.003, 1.969) | 0.0506 |
| Omitting Gladman, et al. 2001 | 98.7% | 1.008 (0.025, 1.992) | 0.0445 |
| Omitting Li, et al. 2008 | 98.7% | 0.997 (0.012, 1.982) | 0.0472 |
| Omitting Xuan, et al. 2011 | 98.7% | 0.983 (-0.004, 1.969) | 0.0509 |
| Omitting Shen, et al. 2012 | 98.7% | 0.996 (0.012, 1.981) | 0.0473 |
| Omitting Lin, et al. 2014 | 98.7% | 1.004 (0.020, 1.988) | 0.0455 |
| Omitting Wang, et al. 2018 | 98.7% | 0.981 (-0.006, 1.968) | 0.0513 |
| Omitting Li, et al. 2021 | 98.7% | 0.991 (0.005, 1.977) | 0.0488 |
| Omitting Vílchez-Oya, et al. 2019 | 98.7% | 1.008 (0.026, 1.990) | 0.0441 |
| Omitting Kwon, et al. 2018 | 98.7% | 1.002 (0.017, 1.986) | 0.0461 |
| Omitting Xu, et al. 2024 | 94.6% | 0.521 (0.076, 0.966) | 0.0217 |
| Omitting Chen, et al. 2021 | 98.6% | 0.944 (-0.042, 1.930) | 0.0606 |
| Before omitting | 98.6% | 0.966 (0.020, 1.913) | 0.0454 |

SLEDAI: systemic lupus erythematosus disease activity index; CI: confidence interval.
