# Supplementary material for: Meta-analysis of factors for osteonecrosis in systemic lupus erythematosus: integration of comprehensive literatures and multicenter databases
Source: Front Immunol. 2026 Jul 2;17:1679237. doi: 10.3389/fimmu.2026.1679237 (PMC13372907; doi:10.3389/fimmu.2026.1679237)
Supplement: Supplementary file 1 [file DataSheet1.zip › Supplementary Material/Supplementary table 38.docx]

Supplementary table 38 Sensitivity analysis for arthritis in the meta-analysis.

| Sensitivity analysis | Heterogeneity (I^2^) | Combined effect size (95% CI) | P value |
| --- | --- | --- | --- |
| Omitting Cheng, et al. 2023 | 12.3% | 1.227 (1.077, 1.398) | 0.0021 |
| Omitting Xiong, et al. 2022 | 9.9% | 1.273 (1.123, 1.444) | 0.0002 |
| Omitting Long, et al. 2021 | 9.3% | 1.219 (1.071, 1.388) | 0.0027 |
| Omitting Dogan, et al. 2020 | 15.2% | 1.256 (1.108, 1.422) | 0.0003 |
| Omitting Hisada, et al. 2018 | 8.5% | 1.278 (1.126, 1.449) | 0.0001 |
| Omitting Tse, et al. 2016 | 15.0% | 1.251 (1.102, 1.421) | 0.0005 |
| Omitting Jokar, et al. 2016 | 15.1% | 1.259 (1.110, 1.428) | 0.0003 |
| Omitting Sheikh, et al. 1998 | 12.8% | 1.256 (1.109, 1.423) | 0.0003 |
| Omitting Watanabe, et al. 1997 | 12.7% | 1.249 (1.103, 1.415) | 0.0005 |
| Omitting Al Saleh, et al. 2010 | 15.0% | 1.255 (1.108, 1.421) | 0.0004 |
| Omitting Massardo, et al. 1992 | 12.1% | 1.246 (1.100, 1.412) | 0.0005 |
| Omitting Ono, et al. 1992 | 15.1% | 1.255 (1.108, 1.421) | 0.0004 |
| Omitting Griffiths, et al. 1979 | 15.1% | 1.255 (1.109, 1.422) | 0.0003 |
| Omitting Calvo-Alen, et al. 2006 | 14.9% | 1.259 (1.112, 1.427) | 0.0003 |
| Omitting Weiner, et al. 1989 | 15.0% | 1.257 (1.110, 1.424) | 0.0003 |
| Omitting Lee, et al. 2013 | 14.3% | 1.248 (1.101, 1.416) | 0.0006 |
| Omitting Faezi, et al. 2014 | 14.9% | 1.248 (1.096, 1.420) | 0.0008 |
| Omitting Sayarlioglu, et al. 2010 | 12.3% | 1.243 (1.097, 1.409) | 0.0007 |
| Omitting Prasad, et al. 2007 | 14.6% | 1.264 (1.114, 1.433) | 0.0003 |
| Omitting Zizic, et al. 1985 | 14.0% | 1.259 (1.112, 1.426) | 0.0003 |
| Omitting Gladman, et al. 2001 | 3.2% | 1.225 (1.080, 1.390) | 0.0016 |
| Omitting Smith, et al. 1976 | 8.9% | 1.247 (1.101, 1.413) | 0.0005 |
| Omitting Qi, et al. 2010 | 15.1% | 1.253 (1.105, 1.421) | 0.0004 |
| Omitting Wu, et al. 2014 | 15.1% | 1.258 (1.110, 1.426) | 0.0003 |
| Omitting Lin, et al. 2014 | 14.9% | 1.254 (1.106, 1.420) | 0.0004 |
| Omitting Li, et al. 2021 | 14.9% | 1.265 (1.113, 1.437) | 0.0003 |
| Omitting Lei, et al. 2024 | 13.5% | 1.244 (1.097, 1.412) | 0.0007 |
| Omitting Zhang, et al. 2008 | 14.7% | 1.253 (1.106, 1.420) | 0.0004 |
| Omitting Liu, et al. 2011 | 13.4% | 1.266 (1.117, 1.436) | 0.0002 |
| Omitting Li, et al. 2014 | 13.2% | 1.245 (1.098, 1.412) | 0.0006 |
| Omitting AHSMU. 2023 | 12.4% | 1.273 (1.122, 1.445) | 0.0002 |
| Omitting WCHSCU. 2020 | 14.6% | 1.253 (1.106, 1.419) | 0.0004 |
| Omitting MHMU. 2023 | 11.5% | 1.271 (1.122, 1.441) | 0.0002 |
| Omitting Vílchez-Oya, et al. 2019 | 14.6% | 1.254 (1.107, 1.420) | 0.0004 |
| Omitting Gladman, et al. 2018 | 10.0% | 1.291 (1.135, 1.469) | 0.0001 |
| Omitting Kwon, et al. 2018 | 3.5% | 1.316 (1.155, 1.500) | <0.0001 |
| Omitting Xu, et al. 2024 | 11.9% | 1.229 (1.081, 1.399) | 0.0017 |
| Omitting Chen, et al. 2021 | 8.2% | 1.285 (1.132, 1.460) | 0.0001 |
| Before omitting | 12.8% | 1.256 (1.109, 1.423) | 0.0003 |

CI: confidence interval; AHSMU: Affiliated Hospital of Southwest Medical University; WCHSCU: West China Hospital of Sichuan University; MHMU: Minda Hospital of Hubei Minzu University.
