# Supplementary material for: Meta-analysis of factors for osteonecrosis in systemic lupus erythematosus: integration of comprehensive literatures and multicenter databases
Source: Front Immunol. 2026 Jul 2;17:1679237. doi: 10.3389/fimmu.2026.1679237 (PMC13372907; doi:10.3389/fimmu.2026.1679237)
Supplement: Supplementary file 1 [file DataSheet1.zip › Supplementary Material/Supplementary table 4.docx]

Supplementary table 4 General information of the patients from MHMU cohort.

| Variable | Total (n=3365) | SLE-ON (n=93) | SLE-non-ON (n=3272) | P value |
| --- | --- | --- | --- | --- |
| Demographic and clinical characteristics |  |  |  |  |
| Female, N (%) | 3119 (92.69) | 75 (80.65) | 3044 (93.03) | <0.001 |
| Age, year | 39.31±14.16 | 36.68±11.70 | 39.38±14.22 | 0.069 |
| Arthritis (+), N (%) | 184 (5.47) | 3 (3.23) | 181 (5.53) | 0.335 |
| Hypertension (+), N (%) | 622 (18.48) | 11 (11.83) | 611 (18.67) | 0.094 |
| Diabetes mellitus (+), N (%) | 151 (4.49) | 4 (4.30) | 147 (4.49) | 1.000 |
| Nephritis (+), N (%) | 1136 (33.76) | 39 (41.94) | 1097 (33.53) | 0.091 |
| Anemia (+), N (%) | 566 (16.82) | 12 (12.90) | 554 (16.93) | 0.306 |
| Cataract (+), N (%) | 60 (1.78) | 0 (0.00) | 60 (1.83) | 0.357 |
| Pulmonary arterial hypertension (+), N (%) | 98 (2.91) | 0 (0.00) | 98 (3.00) | 0.167 |
| Hematologic involvement (+), N (%) | 33 (0.98) | 0 (0.00) | 33 (1.01) | 0.660 |
| Reynaud’s phenomenon (+), N (%) | 9 (0.27) | 0 (0.00) | 9 (0.28) | 1.000 |
| Cushingoid (+), N (%) | 16 (0.48) | 1 (1.08) | 15 (0.46) | 0.930 |
| Osteoporosis (+), N (%) | 340 (10.10) | 7 (7.53) | 333 (10.18) | 0.403 |
| Antiphospholipid syndrome (+), N (%) | 28 (0.83) | 0 (0.00) | 28 (0.86) | 0.751 |
| Sjögren’s syndrome (+), N (%) | 67 (1.99) | 0 (0.00) | 67 (2.05) | 0.309 |
| Laboratory characteristics |  |  |  |  |
| Leukopenia (+), N (%) | 201 (5.97) | 0 (0.00) | 201 (6.14) | 0.014 |
| Thrombocytopenia (+), N (%) | 301 (8.95) | 0 (0.00) | 301 (9.20) | 0.002 |
| RF (+), N (%) | 817 (24.28) | 23 (24.73) | 794 (24.27) | 0.918 |

MHMU: Minda Hospital of Hubei Minzu University; SLE: systemic lupus erythematosus; ON: osteonecrosis; SD: standard deviation; RF: rheumatoid factor.
