# Supplementary material for: Meta-analysis of factors for osteonecrosis in systemic lupus erythematosus: integration of comprehensive literatures and multicenter databases
Source: Front Immunol. 2026 Jul 2;17:1679237. doi: 10.3389/fimmu.2026.1679237 (PMC13372907; doi:10.3389/fimmu.2026.1679237)
Supplement: Supplementary file 1 [file DataSheet1.zip › Supplementary Material/Supplementary table 5.docx]

Supplementary table 5 Comparison of drug use in patients with SLE and SLE-ON.

| Drug | No. of study | Association of ON in patients with SLE | |  | Heterogeneity | | Egger’s test  (P value) |
| --- | --- | --- | --- | --- | --- | --- | --- |
|  |  | OR (95% CI) | P value |  | I^2^, % | P value |  |
| Immunosuppressants |  |  |  |  |  |  |  |
| Cyclophosphamide^a^ | 14 | 1.869 (1.264, 2.765) | 0.0017 |  | 79.1 | <0.0001 | 0.8148 |
| Azathioprine^a^ | 9 | 1.055 (0.689, 1.614) | 0.8051 |  | 62.5 | 0.0064 | 0.9510 |
| Mycophenolate Mofetil^a^ | 11 | 1.410 (0.840, 2.368) | 0.1937 |  | 83.7 | <0.0001 | 0.4396 |
| Methotrexate | 5 | 1.024 (0.779, 1.345) | 0.8656 |  | 0.0 | 0.4290 | 0.5846 |
| Cyclosporine | 5 | 0.999 (0.646, 1.543) | 0.9952 |  | 0.0 | 0.4589 | 0.2626 |
| Bisphosphonate | 3 | 0.689 (0.450, 1.057) | 0.0880 |  | 0.0 | 0.5264 | 0.7543 |
| Hydroxychloroquine^a^ | 24 | 0.778 (0.560, 1.081) | 0.1350 |  | 80.2 | <0.0001 | 0.0748 |
| Steroid pulse therapy^a^ | 28 | 1.829 (1.460, 2.291) | <0.0001 |  | 36.8 | 0.0279 | 0.3781 |

^a^random-effects model; SLE: systemic lupus erythematosus; ON: osteonecrosis; OR: odd ratio; CI: confidence interval
