# Supplementary material for: Meta-analysis of factors for osteonecrosis in systemic lupus erythematosus: integration of comprehensive literatures and multicenter databases
Source: Front Immunol. 2026 Jul 2;17:1679237. doi: 10.3389/fimmu.2026.1679237 (PMC13372907; doi:10.3389/fimmu.2026.1679237)
Supplement: Supplementary file 1 [file DataSheet1.zip › Supplementary Material/Supplementary table 6.docx]

Supplementary table 6 Comparison of general situation in patients with SLE and SLE-ON.

| General situation | No. of study | Association of ON in patients with SLE | |  | Heterogeneity | | Egger’s test  (P value) |
| --- | --- | --- | --- | --- | --- | --- | --- |
|  |  | SMD (95% CI) | P value |  | I^2^, % | P value |  |
| Age, year^a^ | 31 | -0.175 (-0.323, -0.027) | 0.0203 |  | 74.6 | <0.0001 | 0.3944 |
| Age at onset, year^a^ | 21 | -0.426 (-0.842, -0.009) | 0.0452 |  | 91.9 | <0.0001 | 0.4293 |
| Disease duration, year^a^ | 22 | 0.632 (-0.128, 1.391) | 0.1033 |  | 97.2 | <0.0001 | 0.5460 |
| SLEDAI^a^ | 25 | 0.966 ( 0.020, 1.913) | 0.0454 |  | 98.6 | <0.0001 | 0.0498 |

^a^random-effects model; SLE: systemic lupus erythematosus; ON: osteonecrosis; SMD: standardized mean difference; CI: confidence interval; SLEDAI: SLE disease activity index.
