# Supplementary material for: Meta-analysis of factors for osteonecrosis in systemic lupus erythematosus: integration of comprehensive literatures and multicenter databases
Source: Front Immunol. 2026 Jul 2;17:1679237. doi: 10.3389/fimmu.2026.1679237 (PMC13372907; doi:10.3389/fimmu.2026.1679237)
Supplement: Supplementary file 1 [file DataSheet1.zip › Supplementary Material/Supplementary table 7.docx]

Supplementary table 7 Sensitivity analysis for hypertension in the meta-analysis.

| Sensitivity analysis | Heterogeneity (I^2^) | Combined effect size (95% CI) | P value |
| --- | --- | --- | --- |
| Omitting Abdelkawy, et al. 2022 | 46.4% | 1.371 (1.206, 1.559) | <0.0001 |
| Omitting Xiong, et al. 2022 | 45.2% | 1.377 (1.214, 1.562) | <0.0001 |
| Omitting Long, et al. 2021 | 43.5% | 1.413 (1.245, 1.604) | <0.0001 |
| Omitting Shaharir, et al. 2021 | 45.7% | 1.406 (1.237, 1.599) | <0.0001 |
| Omitting Dogan, et al. 2020 | 45.8% | 1.379 (1.216, 1.564) | <0.0001 |
| Omitting Tse, et al. 2016 | 44.4% | 1.372 (1.209, 1.556) | <0.0001 |
| Omitting Sekiya, et al. 2009 | 46.8% | 1.388 (1.224, 1.574) | <0.0001 |
| Omitting Mok, et al. 1998 | 46.7% | 1.384 (1.221, 1.570) | <0.0001 |
| Omitting Al Saleh, et al. 2010 | 46.8% | 1.386 (1.222, 1.572) | <0.0001 |
| Omitting Joo, et al. 2014 | 40.9% | 1.233 (1.059, 1.437) | 0.0072 |
| Omitting Calvo-Alen, et al. 2006 | 46.8% | 1.385 (1.220, 1.572) | <0.0001 |
| Omitting Nagasawa, et al. 1989 | 46.8% | 1.388 (1.224, 1.574) | <0.0001 |
| Omitting Lee, et al. 2013 | 45.0% | 1.369 (1.206, 1.554) | <0.0001 |
| Omitting Sayarlioglu, et al. 2010 | 46.4% | 1.376 (1.211, 1.563) | <0.0001 |
| Omitting Prasad, et al. 2007 | 46.6% | 1.395 (1.228, 1.584) | <0.0001 |
| Omitting Zizic, et al. 1985 | 46.7% | 1.384 (1.221, 1.570) | <0.0001 |
| Omitting Uea-areewongsa, et al. 2009 | 46.8% | 1.387 (1.223, 1.572) | <0.0001 |
| Omitting Kunyakham, et al. 2012 | 46.8% | 1.389 (1.223, 1.578) | <0.0001 |
| Omitting Smith, et al. 1976 | 39.0% | 1.407 (1.241, 1.596) | <0.0001 |
| Omitting Qi, et al. 2010 | 46.8% | 1.388 (1.223, 1.575) | <0.0001 |
| Omitting Wu, et al. 2014 | 46.9% | 1.387 (1.223, 1.573) | <0.0001 |
| Omitting Li, et al. 2021 | 46.3% | 1.371 (1.207, 1.559) | <0.0001 |
| Omitting Tang, et al. 1999 | 44.1% | 1.402 (1.236, 1.590) | <0.0001 |
| Omitting Vilchez-Oya, et al. 2019 | 43.1% | 1.376 (1.214, 1.561) | <0.0001 |
| Omitting Xu, et al. 2024 | 45.0% | 1.411 (1.241, 1.604) | <0.0001 |
| Omitting Wang, et al. 2009 | 46.8% | 1.386 (1.222, 1.573) | <0.0001 |
| Omitting AHSMU. 2023 | 46.8% | 1.380 (1.211, 1.572) | <0.0001 |
| Omitting WCHSCU. 2020 | 27.0% | 1.470 (1.293, 1.671) | <0.0001 |
| Omitting MHMU. 2023 | 36.7% | 1.452 (1.277, 1.652) | <0.0001 |
| Before omitting | 44.9% | 1.388 (1.224, 1.573) | <0.0001 |

CI: confidence interval; AHSMU: Affiliated Hospital of Sothwest Medical University; WCHSCU: West China Hospital of Sichuan University; MHMU: Minda Hospital of Hubei Minzu University.
