# Supplementary material for: Meta-analysis of factors for osteonecrosis in systemic lupus erythematosus: integration of comprehensive literatures and multicenter databases
Source: Front Immunol. 2026 Jul 2;17:1679237. doi: 10.3389/fimmu.2026.1679237 (PMC13372907; doi:10.3389/fimmu.2026.1679237)
Supplement: Supplementary file 1 [file DataSheet1.zip › Supplementary Material/Supplementary table 8.docx]

Supplementary table 8 Sensitivity analysis for neuropsychiatric lupus in the meta-analysis.

| Sensitivity analysis | Heterogeneity (I^2^) | Combined effect size (95% CI) | P value |
| --- | --- | --- | --- |
| Omitting Cheng, et al. 2023 | 0.0% | 1.646 (1.373, 1.972) | <0.0001 |
| Omitting Xiong, et al. 2022 | 0.0% | 1.638 (1.378, 1.947) | <0.0001 |
| Omitting Long, et al. 2021 | 0.0% | 1.605 (1.342, 1.919) | <0.0001 |
| Omitting Shaharir, et al. 2021 | 0.0% | 1.669 (1.398, 1.991) | <0.0001 |
| Omitting Dogan, et al. 2020 | 0.0% | 1.659 (1.395, 1.972) | <0.0001 |
| Omitting Hisada, et al. 2018 | 0.0% | 1.653 (1.388, 1.969) | <0.0001 |
| Omitting Tse, et al. 2016 | 0.0% | 1.617 (1.353, 1.932) | <0.0001 |
| Omitting Watanabe, et al. 1997 | 0.0% | 1.643 (1.382, 1.953) | <0.0001 |
| Omitting Al Saleh, et al. 2010 | 0.0% | 1.634 (1.374, 1.944) | <0.0001 |
| Omitting Ono, et al. 1992 | 0.0% | 1.653 (1.391, 1.964) | <0.0001 |
| Omitting Lee, et al. 2013 | 0.0% | 1.606 (1.347, 1.915) | <0.0001 |
| Omitting Faezi, et al. 2014 | 0.0% | 1.708 (1.426, 2.046) | <0.0001 |
| Omitting Sayarlioglu, et al. 2010 | 0.0% | 1.662 (1.397, 1.976) | <0.0001 |
| Omitting Prasad, et al. 2007 | 0.0% | 1.675 (1.406, 1.994) | <0.0001 |
| Omitting Zizic, et al. 1985 | 0.0% | 1.667 (1.401, 1.985) | <0.0001 |
| Omitting Uea-areewongsa, et al. 2009 | 0.0% | 1.701 (1.430, 2.022) | <0.0001 |
| Omitting Gladman, et al. 2001 | 0.0% | 1.704 (1.426, 2.036) | <0.0001 |
| Omitting Kunyakham, et al. 2012 | 0.0% | 1.643 (1.375, 1.964) | <0.0001 |
| Omitting Wu, et al. 2014 | 0.0% | 1.674 (1.407, 1.991) | <0.0001 |
| Omitting Li, et al. 2021 | 0.0% | 1.658 (1.385, 1.986) | <0.0001 |
| Omitting Lei, et al. 2024 | 0.0% | 1.626 (1.363, 1.939) | <0.0001 |
| Omitting Li, et al. 2014 | 0.0% | 1.662 (1.397, 1.978) | <0.0001 |
| Omitting Kwon, et al. 2018 | 0.0% | 1.621 (1.355, 1.939) | <0.0001 |
| Omitting Xu, et al. 2024 | 0.0% | 1.652 (1.386, 1.969) | <0.0001 |
| Omitting Chen, et al. 2021 | 0.0% | 1.642 (1.380, 1.953) | <0.0001 |
| Omitting AHSMU. 2023 | 0.0% | 1.653 (1.391, 1.965) | <0.0001 |
| Before omitting | 0.0% | 1.652 (1.391, 1.963) | <0.0001 |

CI: confidence interval; AHSMU: Affiliated Hospital of Southwest Medical University.
