# Supplementary material for: Meta-analysis of factors for osteonecrosis in systemic lupus erythematosus: integration of comprehensive literatures and multicenter databases
Source: Front Immunol. 2026 Jul 2;17:1679237. doi: 10.3389/fimmu.2026.1679237 (PMC13372907; doi:10.3389/fimmu.2026.1679237)
Supplement: Supplementary file 1 [file DataSheet1.zip › Supplementary Material/Supplementary table 9.docx]

Supplementary table 9 Sensitivity analysis for hyperlipidemia in the meta-analysis.

| Sensitivity analysis | Heterogeneity (I^2^) | Combined effect size (95% CI) | P value |
| --- | --- | --- | --- |
| Omitting Xiong, et al. 2022 | 21.2% | 1.382 (1.086, 1.758) | 0.0085 |
| Omitting Long, et al. 2021 | 21.9% | 1.380 (1.083, 1.759) | 0.0092 |
| Omitting Shaharir, et al. 2021 | 17.0% | 1.305 (1.013, 1.681) | 0.0395 |
| Omitting Dogan, et al. 2020 | 0.0% | 1.315 (1.037, 1.668) | 0.0238 |
| Omitting Faezi, et al. 2014 | 0.0% | 1.482 (1.137, 1.932) | 0.0037 |
| Omitting Li, et al. 2021 | 25.1% | 1.345 (1.034, 1.749) | 0.0272 |
| Omitting Xu, et al. 2024 | 23.5% | 1.312 (0.996, 1.728) | 0.0532 |
| Before omitting | 10.3% | 1.358 (1.075, 1.717) | 0.0103 |

CI: confidence interval.
