# Supplementary figures and images for: Meta-analysis of factors for osteonecrosis in systemic lupus erythematosus: integration of comprehensive literatures and multicenter databases
Source: Front Immunol. 2026 Jul 2;17:1679237. doi: 10.3389/fimmu.2026.1679237 (PMC13372907; doi:10.3389/fimmu.2026.1679237)

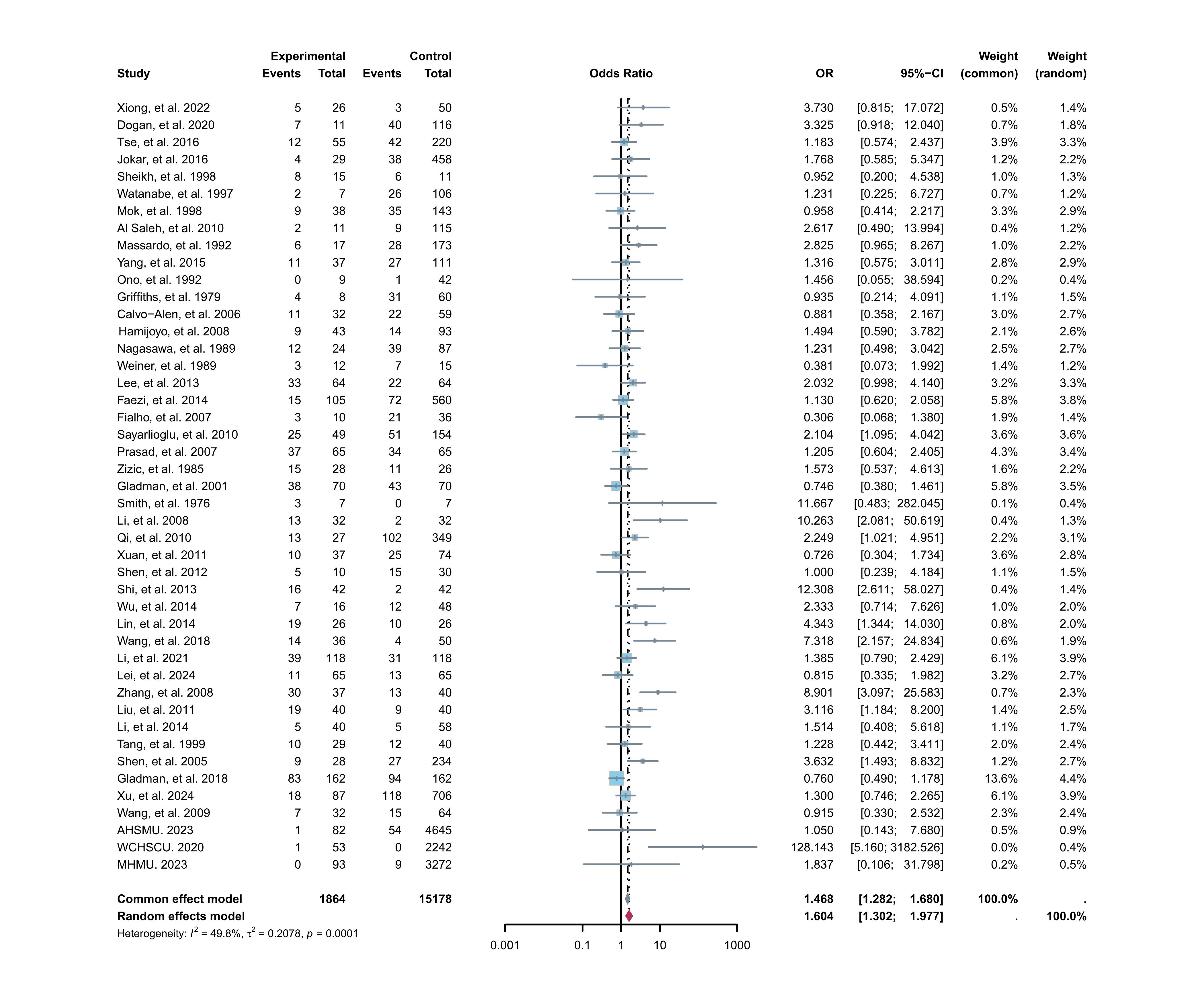

Supplement: Supplementary file 1 [file DataSheet1.zip › Supplementary Material/Supplementary figure 15.tif]

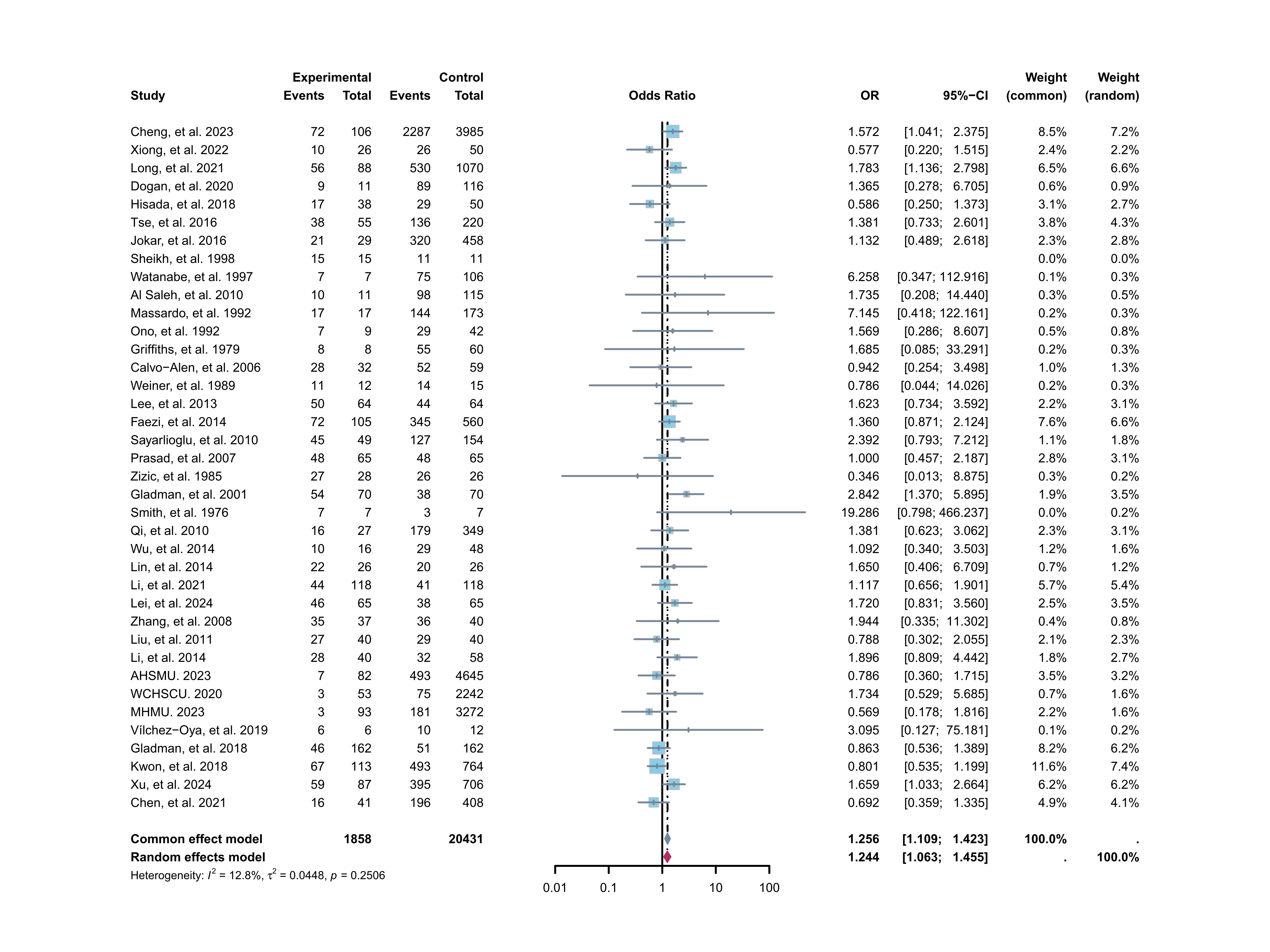

Supplement: Supplementary file 1 [file DataSheet1.zip › Supplementary Material/Supplementary figure 19.tif]

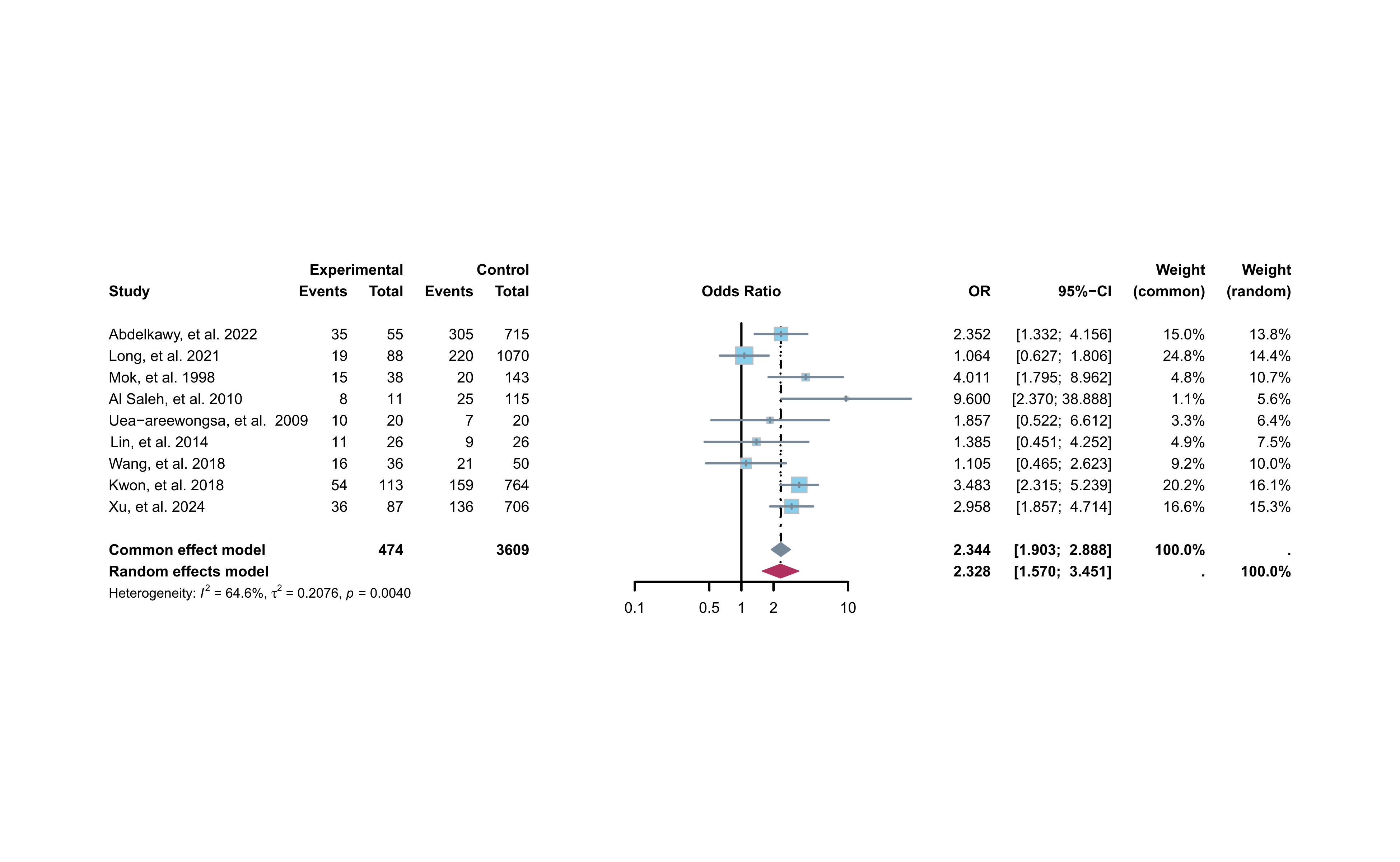

Supplement: Supplementary file 1 [file DataSheet1.zip › Supplementary Material/Supplementary figure 27.tif]

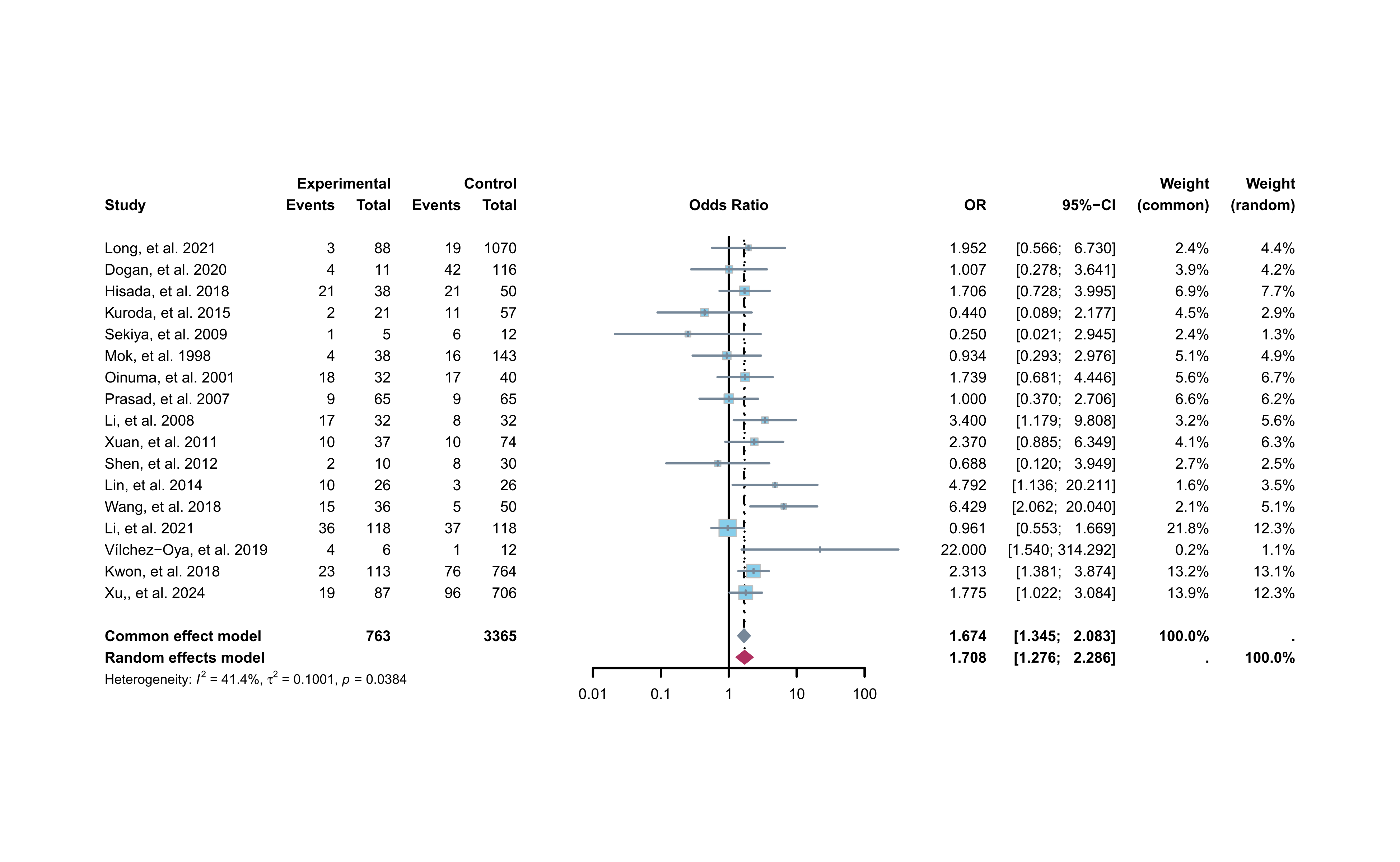

Supplement: Supplementary file 1 [file DataSheet1.zip › Supplementary Material/Supplementary figure 29.tif]

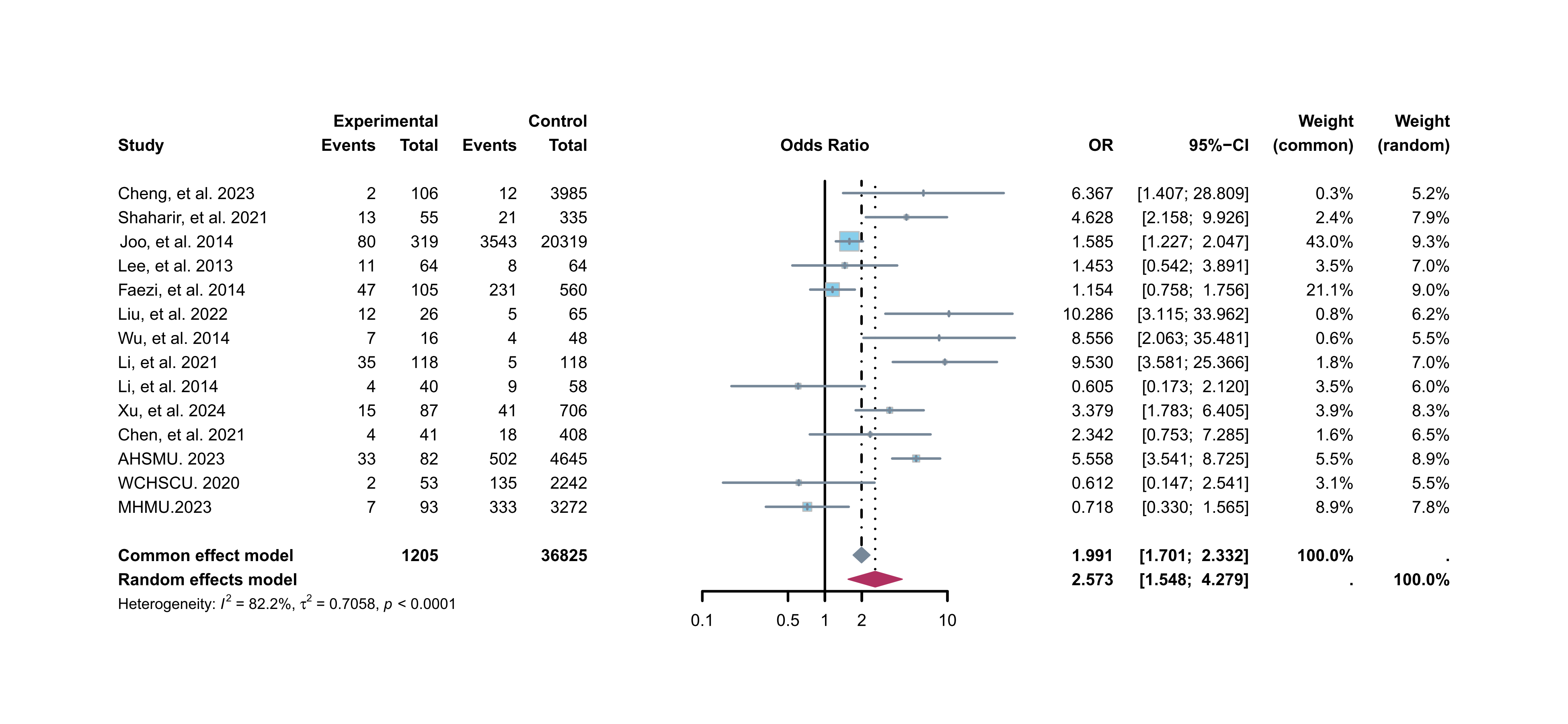

Supplement: Supplementary file 1 [file DataSheet1.zip › Supplementary Material/Supplementary figure 33.tif]

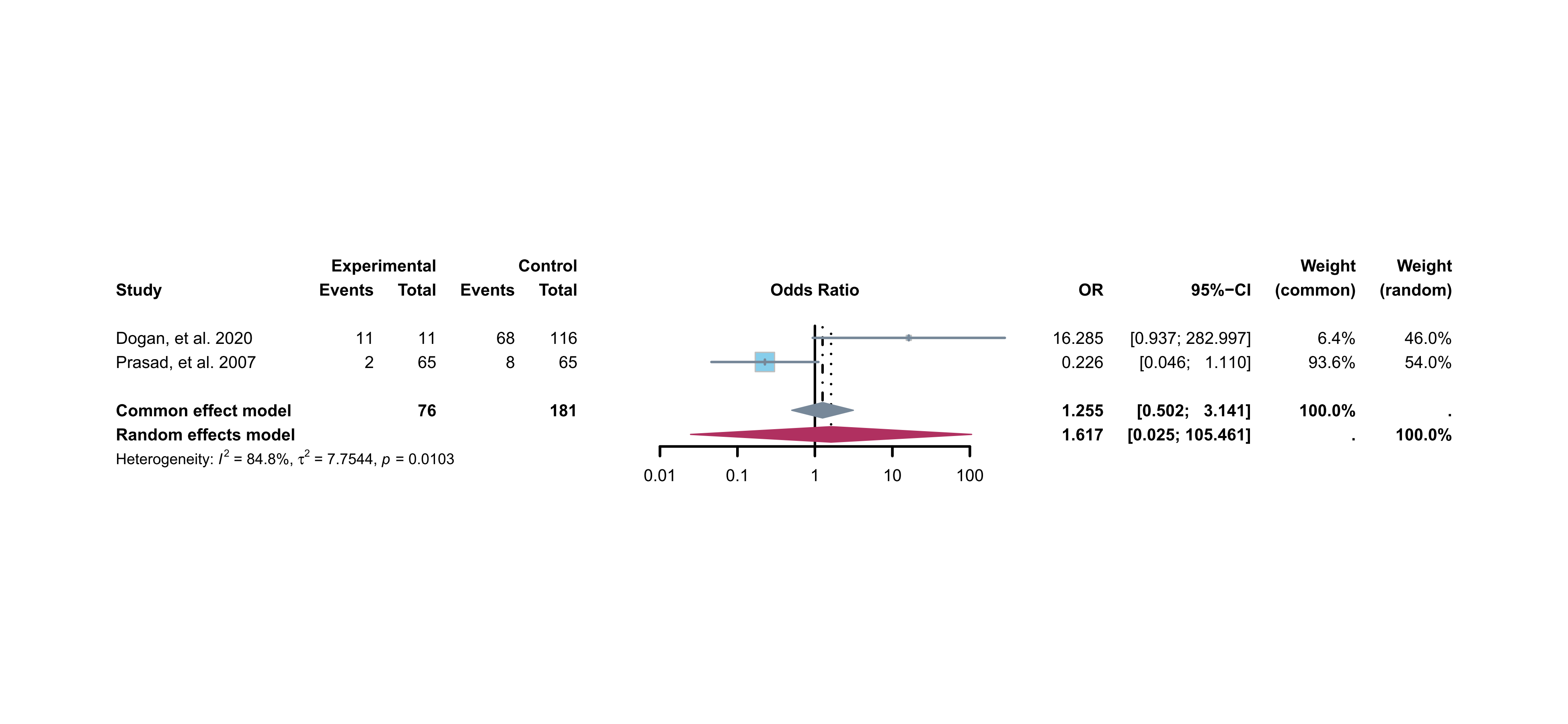

Supplement: Supplementary file 1 [file DataSheet1.zip › Supplementary Material/Supplementary figure 34.tif]

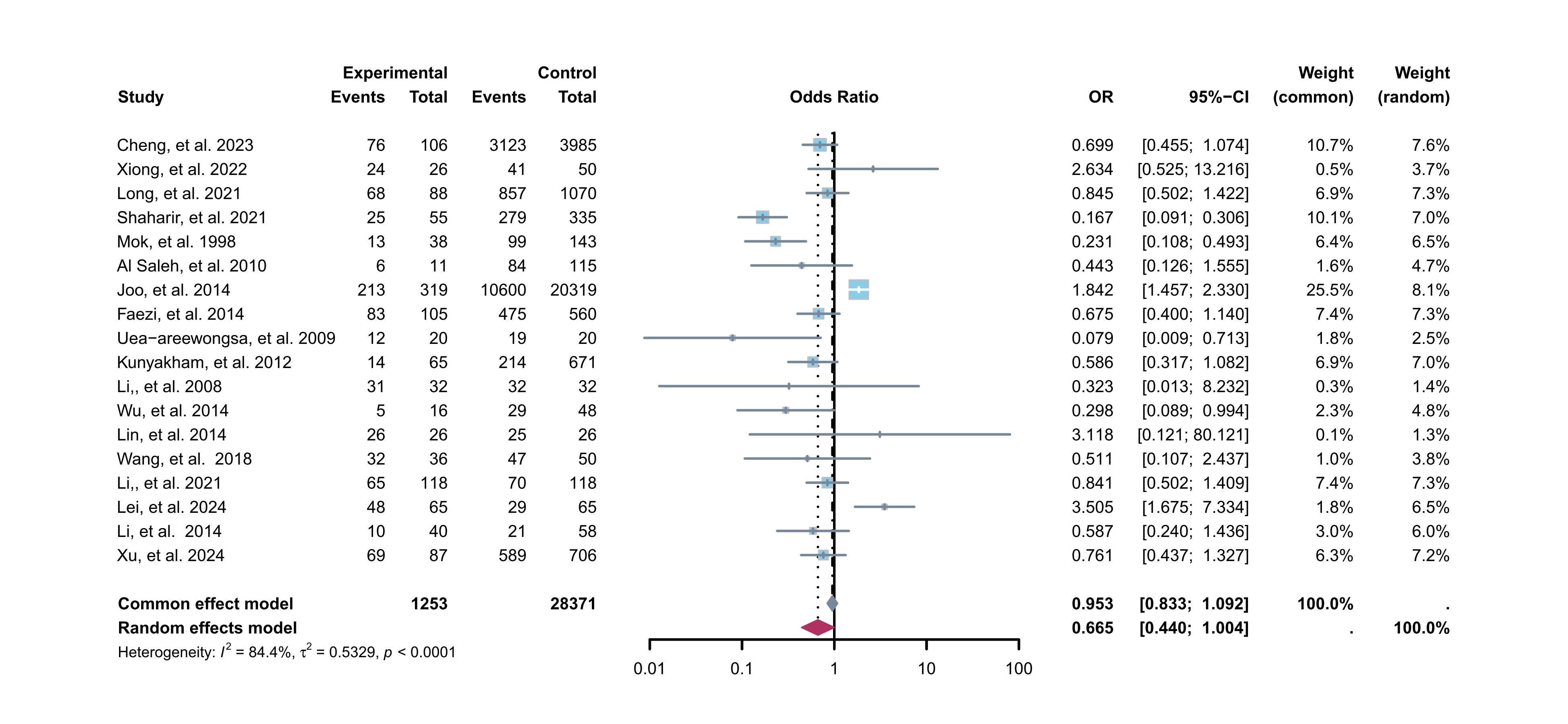

Supplement: Supplementary file 1 [file DataSheet1.zip › Supplementary Material/Supplementary figure 35.tif]

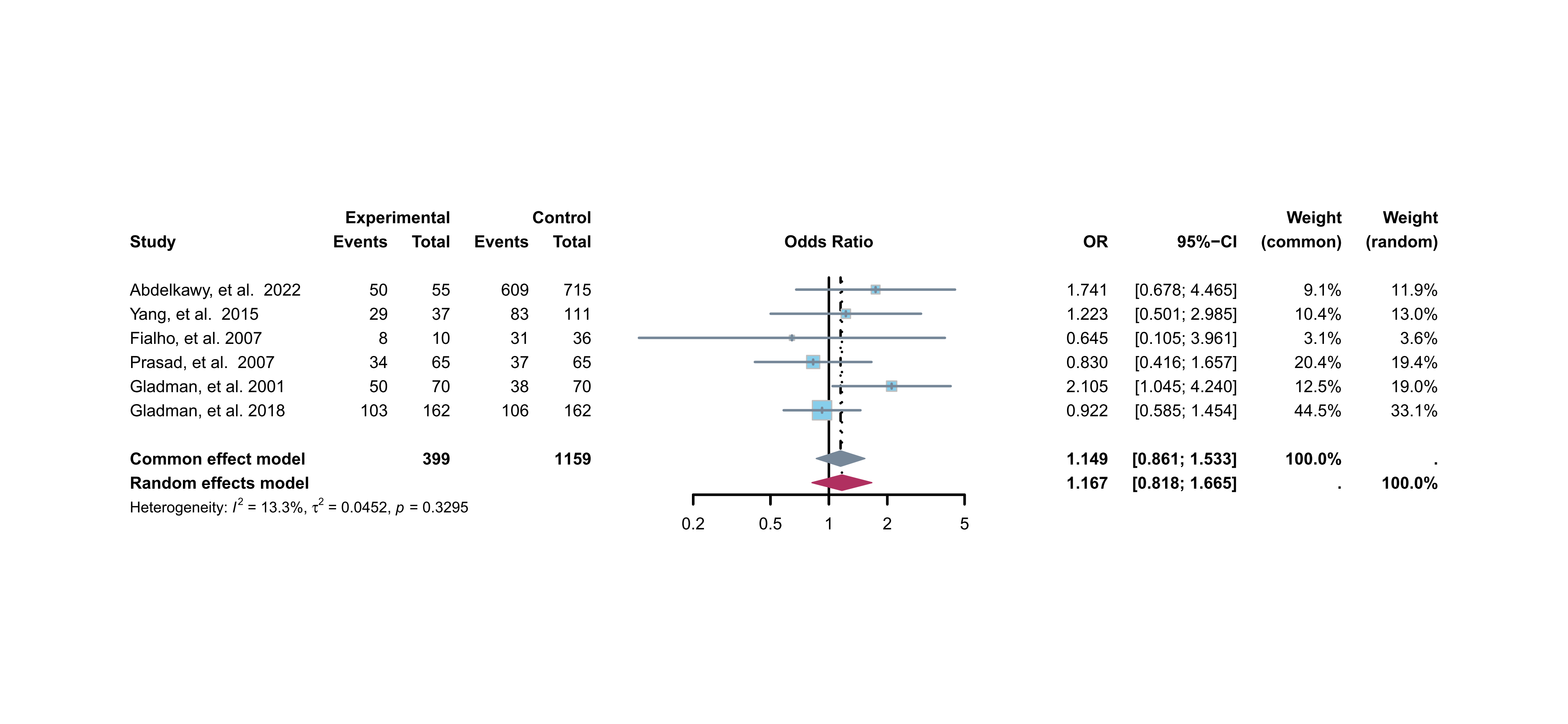

Supplement: Supplementary file 1 [file DataSheet1.zip › Supplementary Material/Supplementary figure 36.tif]

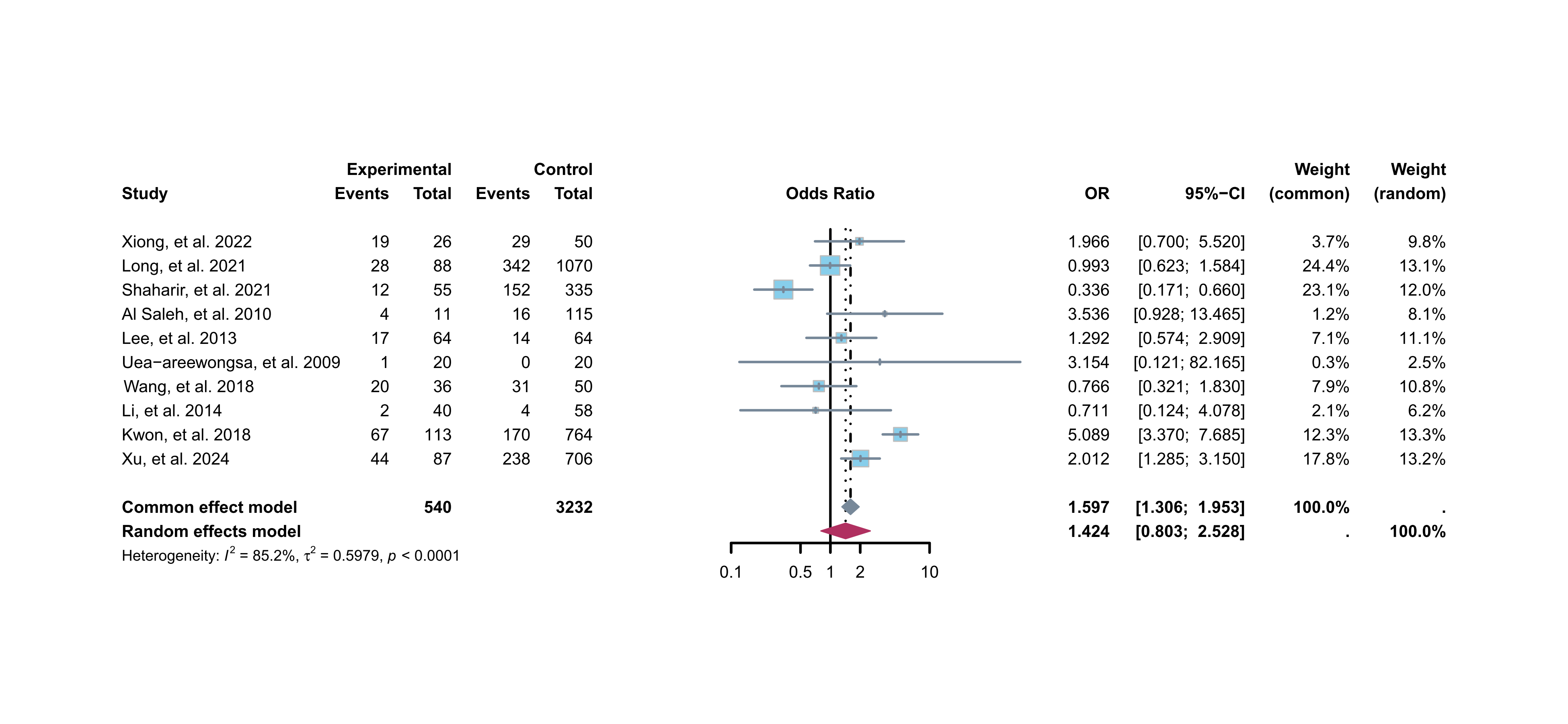

Supplement: Supplementary file 1 [file DataSheet1.zip › Supplementary Material/Supplementary figure 37.tif]

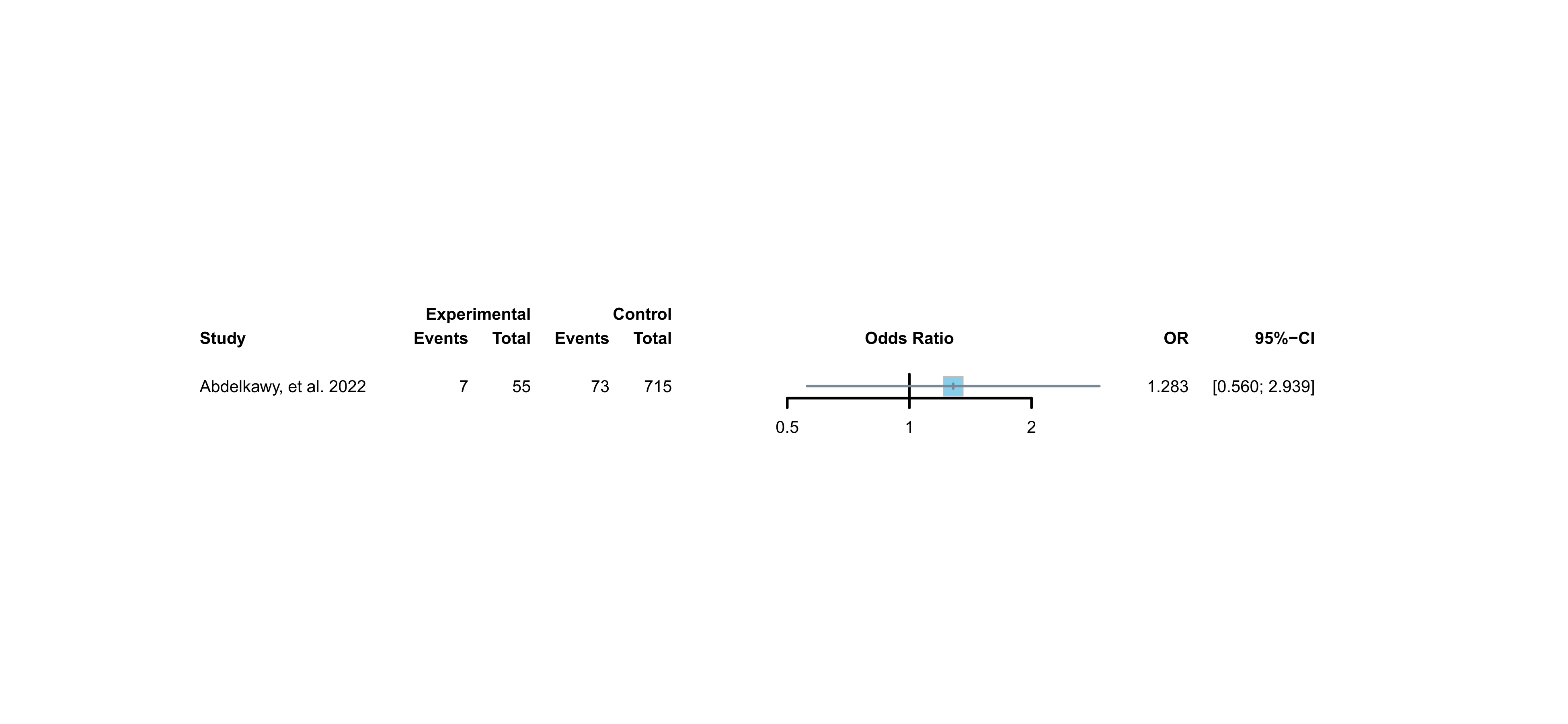

Supplement: Supplementary file 1 [file DataSheet1.zip › Supplementary Material/Supplementary figure 38.tif]

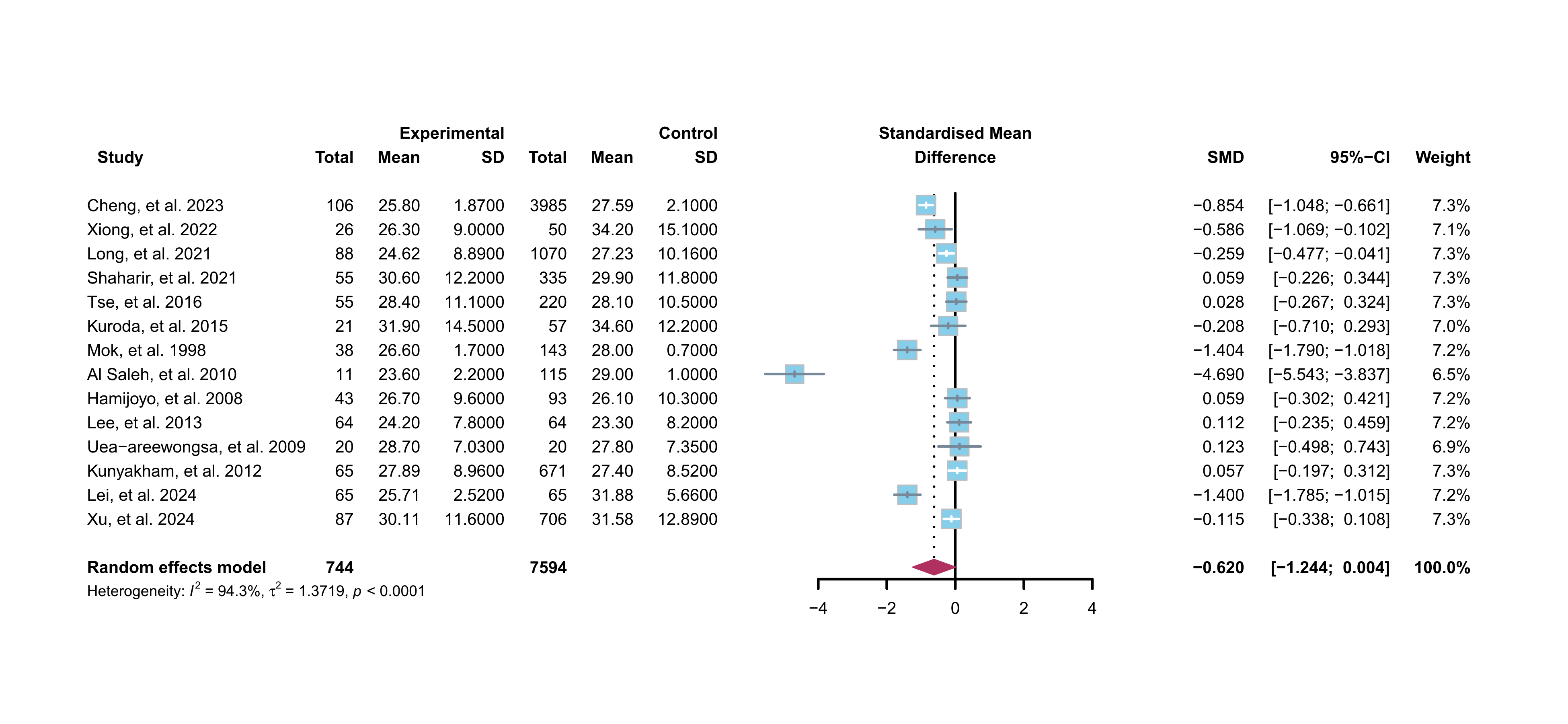

Supplement: Supplementary file 1 [file DataSheet1.zip › Supplementary Material/Supplementary figure 39.tif]

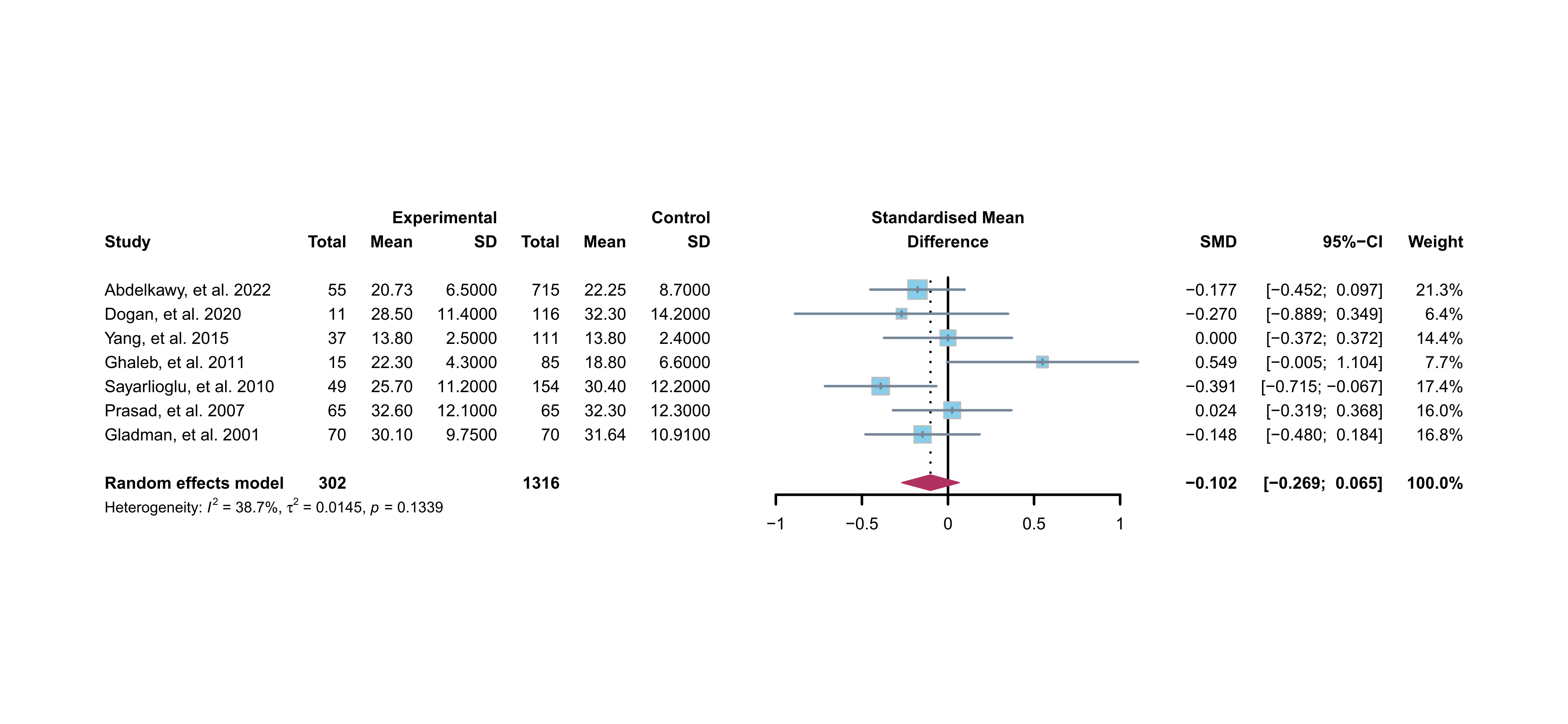

Supplement: Supplementary file 1 [file DataSheet1.zip › Supplementary Material/Supplementary figure 40.tif]

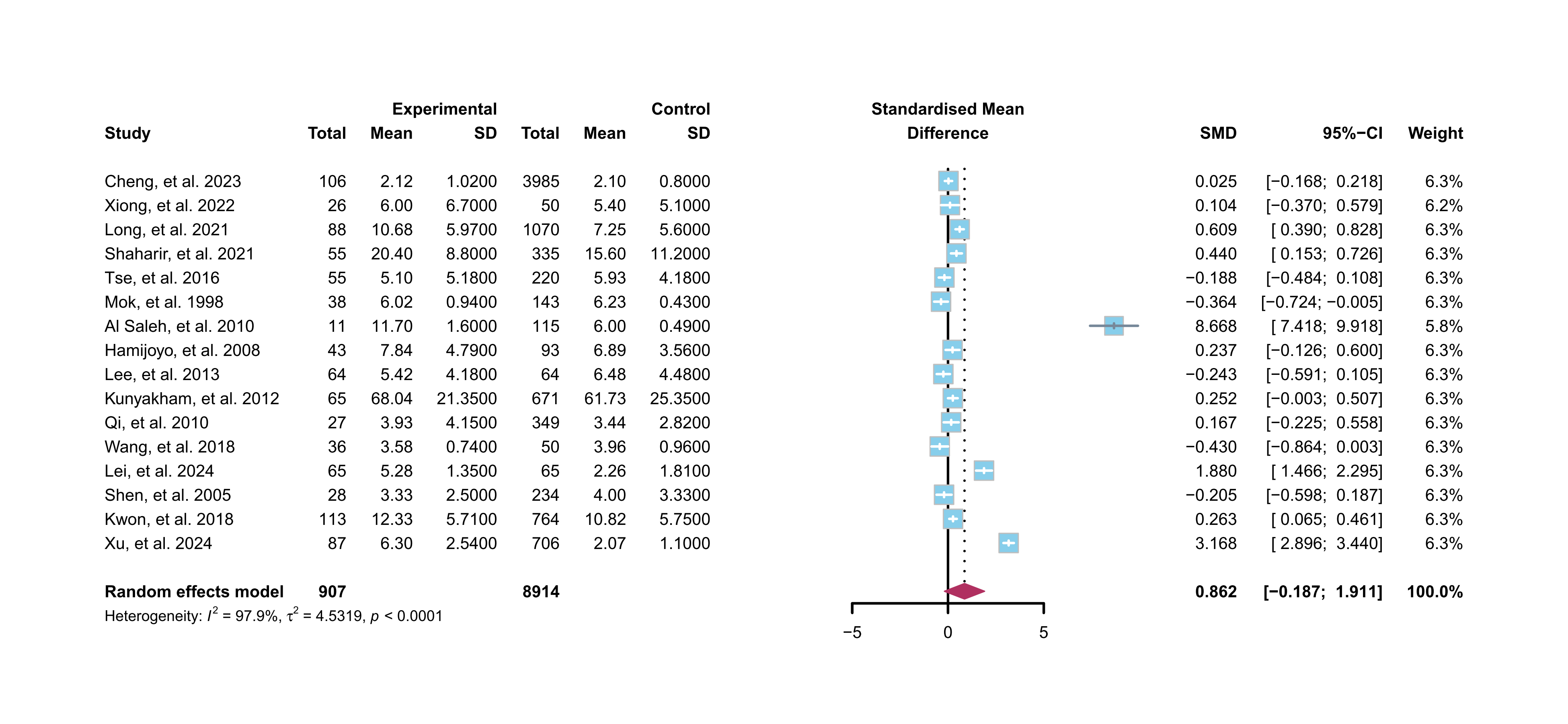

Supplement: Supplementary file 1 [file DataSheet1.zip › Supplementary Material/Supplementary figure 41.tif]

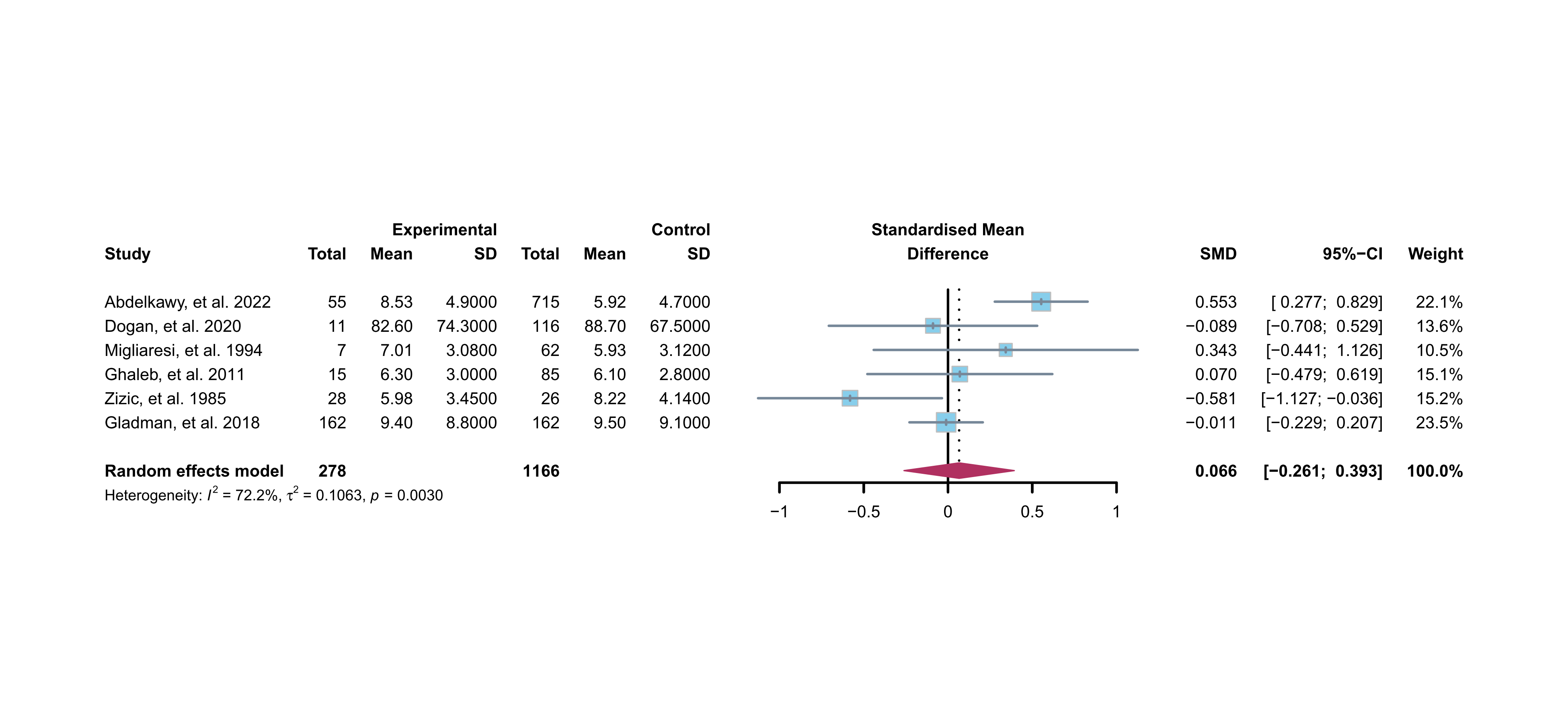

Supplement: Supplementary file 1 [file DataSheet1.zip › Supplementary Material/Supplementary figure 42.tif]

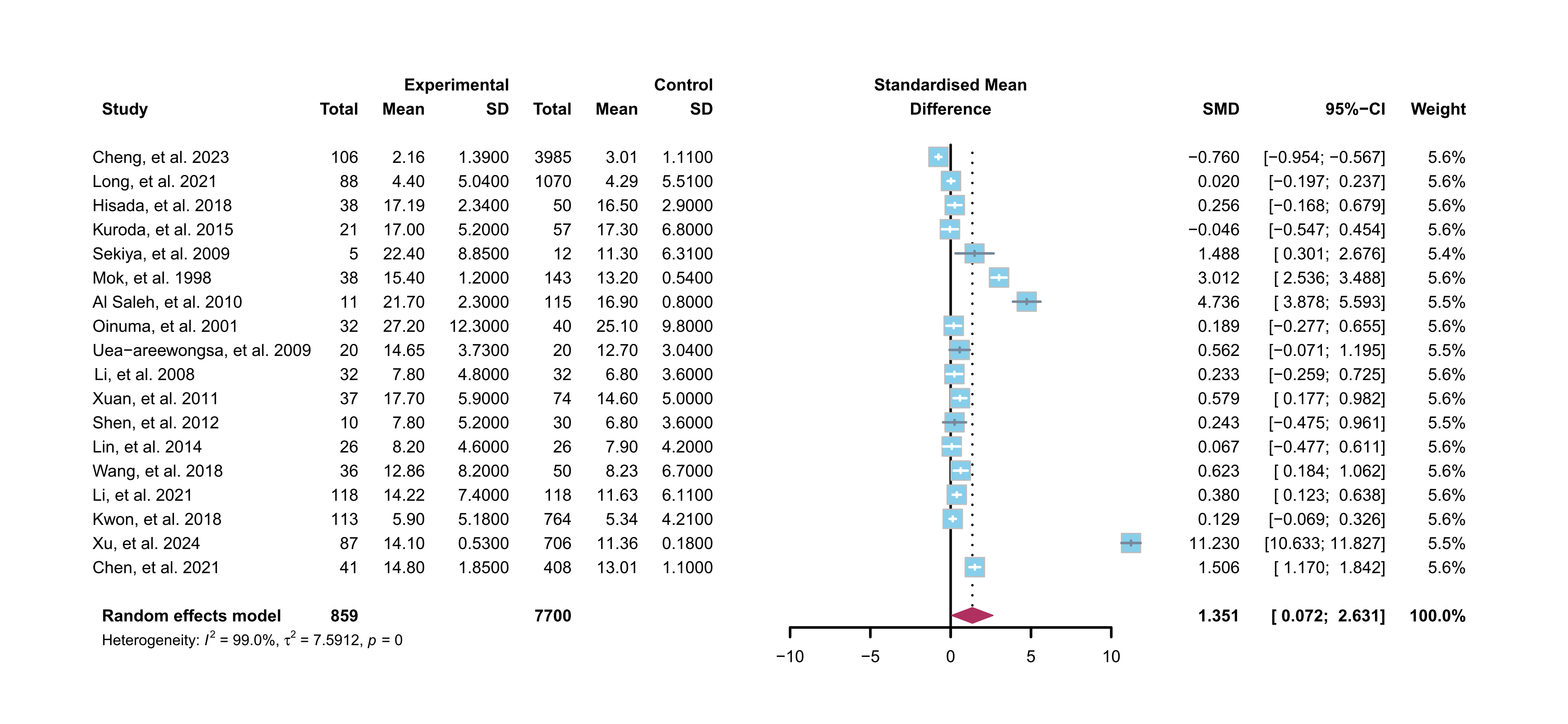

Supplement: Supplementary file 1 [file DataSheet1.zip › Supplementary Material/Supplementary figure 43.tif]

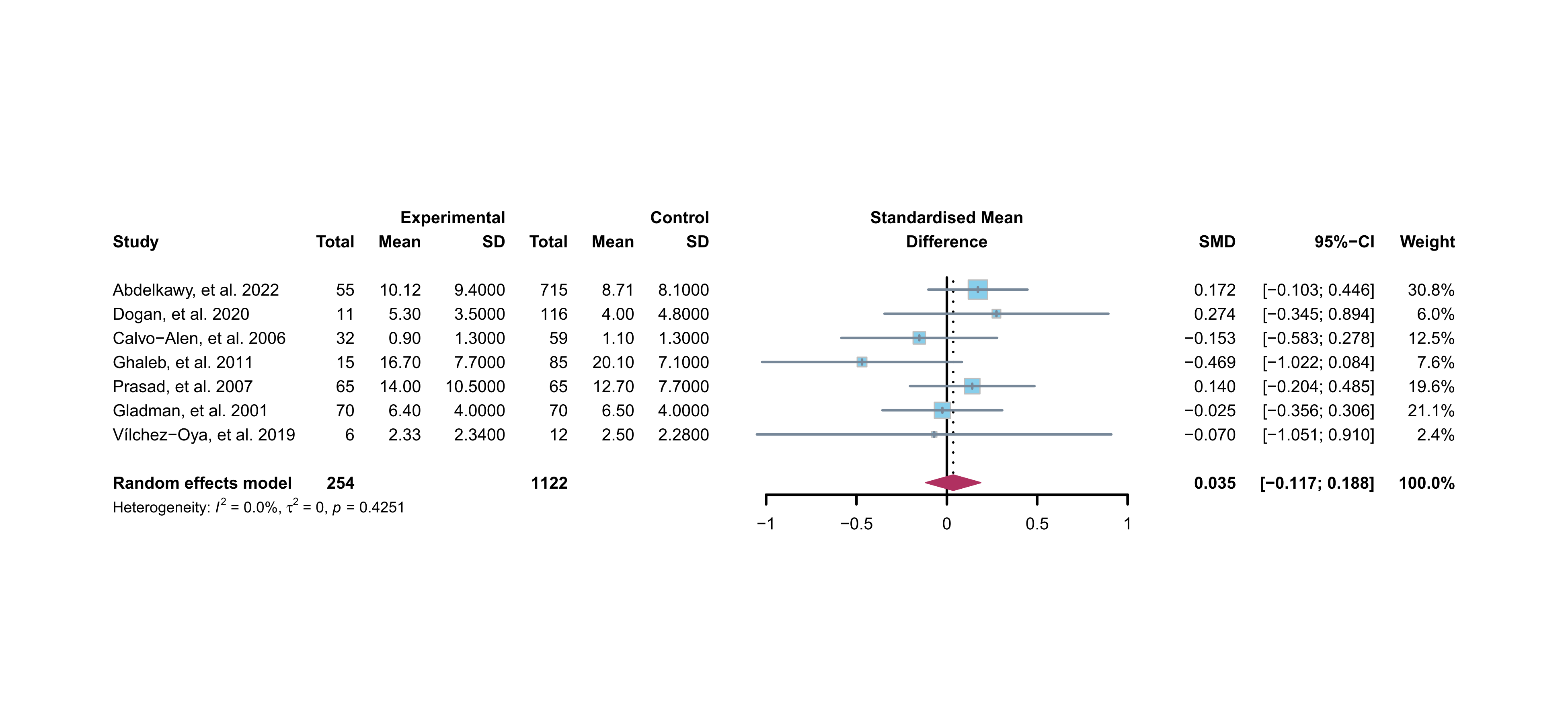

Supplement: Supplementary file 1 [file DataSheet1.zip › Supplementary Material/Supplementary figure 44.tif]

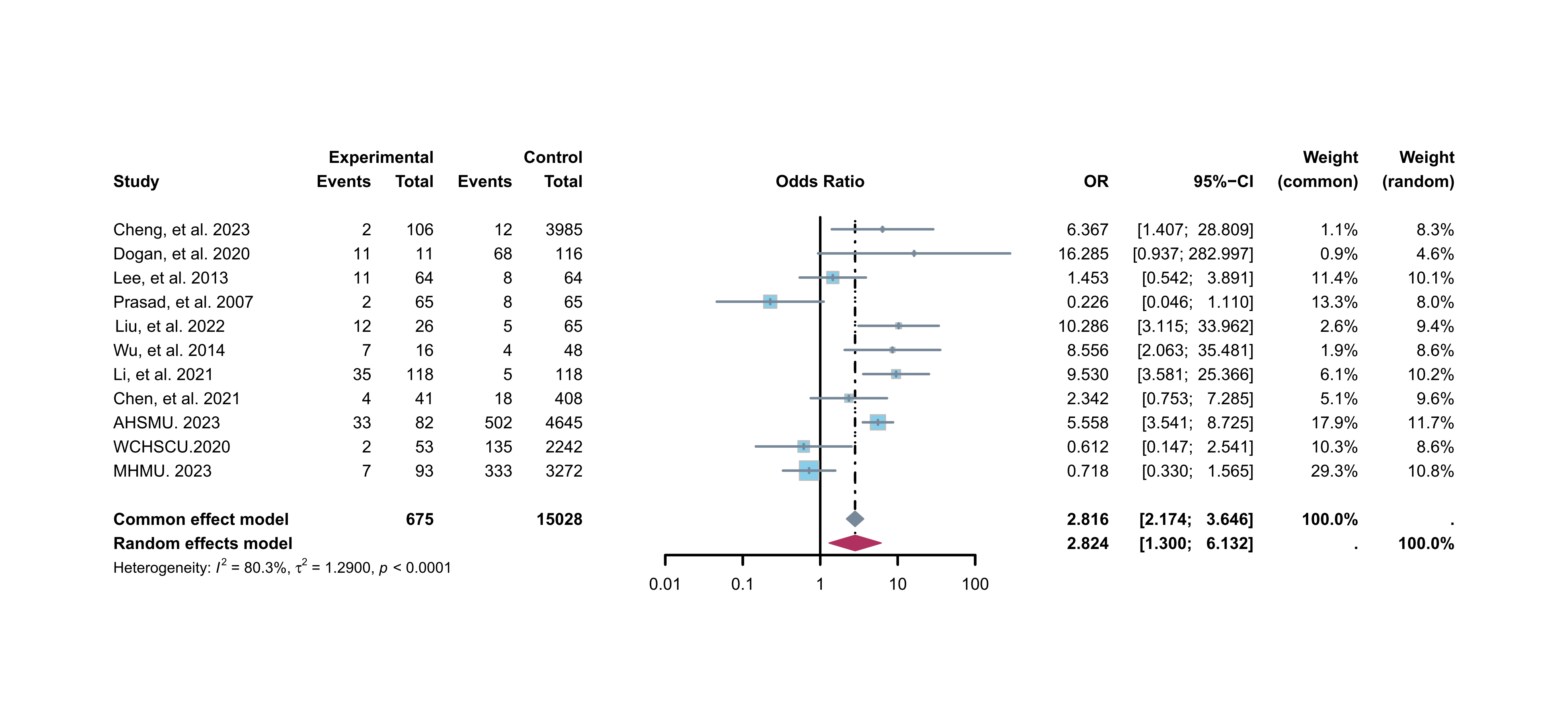

Supplement: Supplementary file 1 [file DataSheet1.zip › Supplementary Material/Supplementary figure 45.tif]

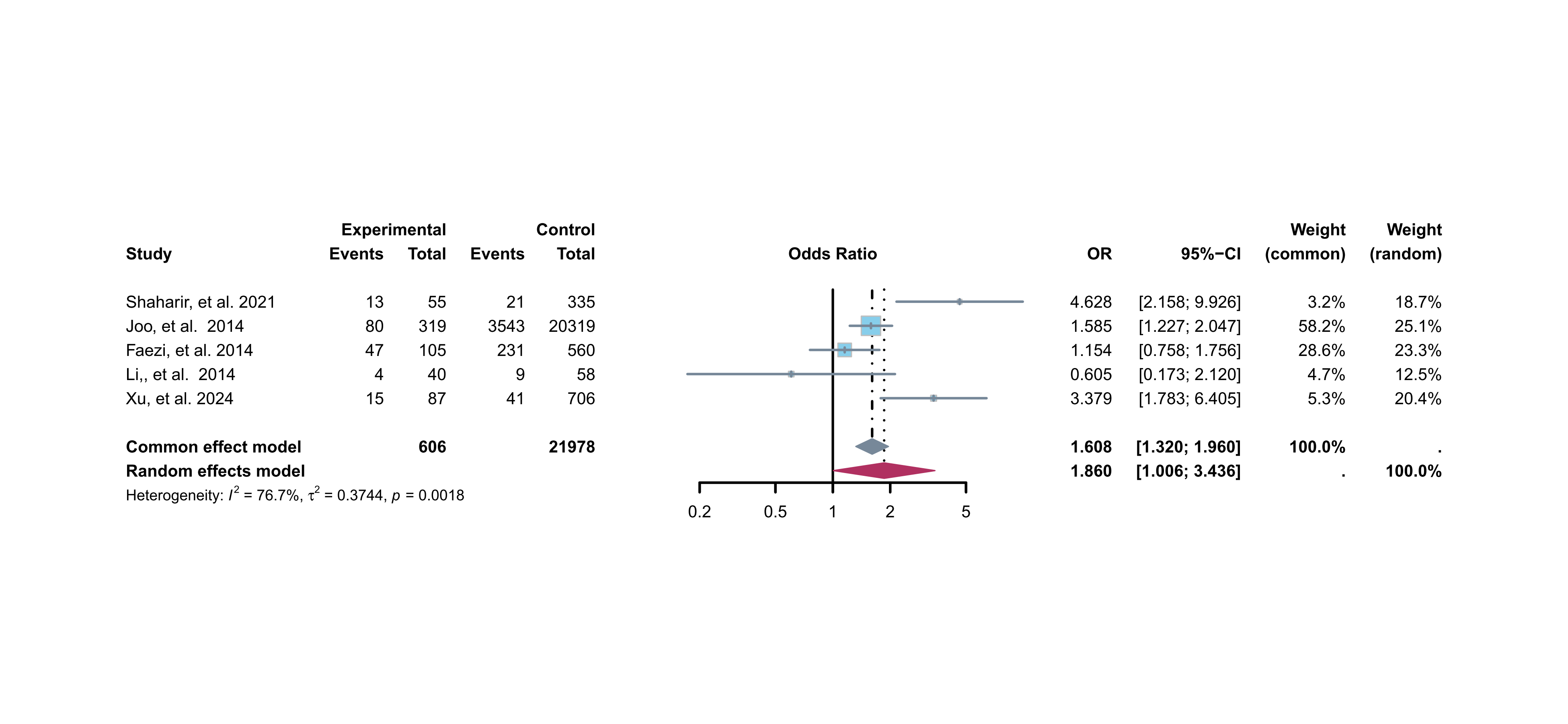

Supplement: Supplementary file 1 [file DataSheet1.zip › Supplementary Material/Supplementary figure 46.tif]

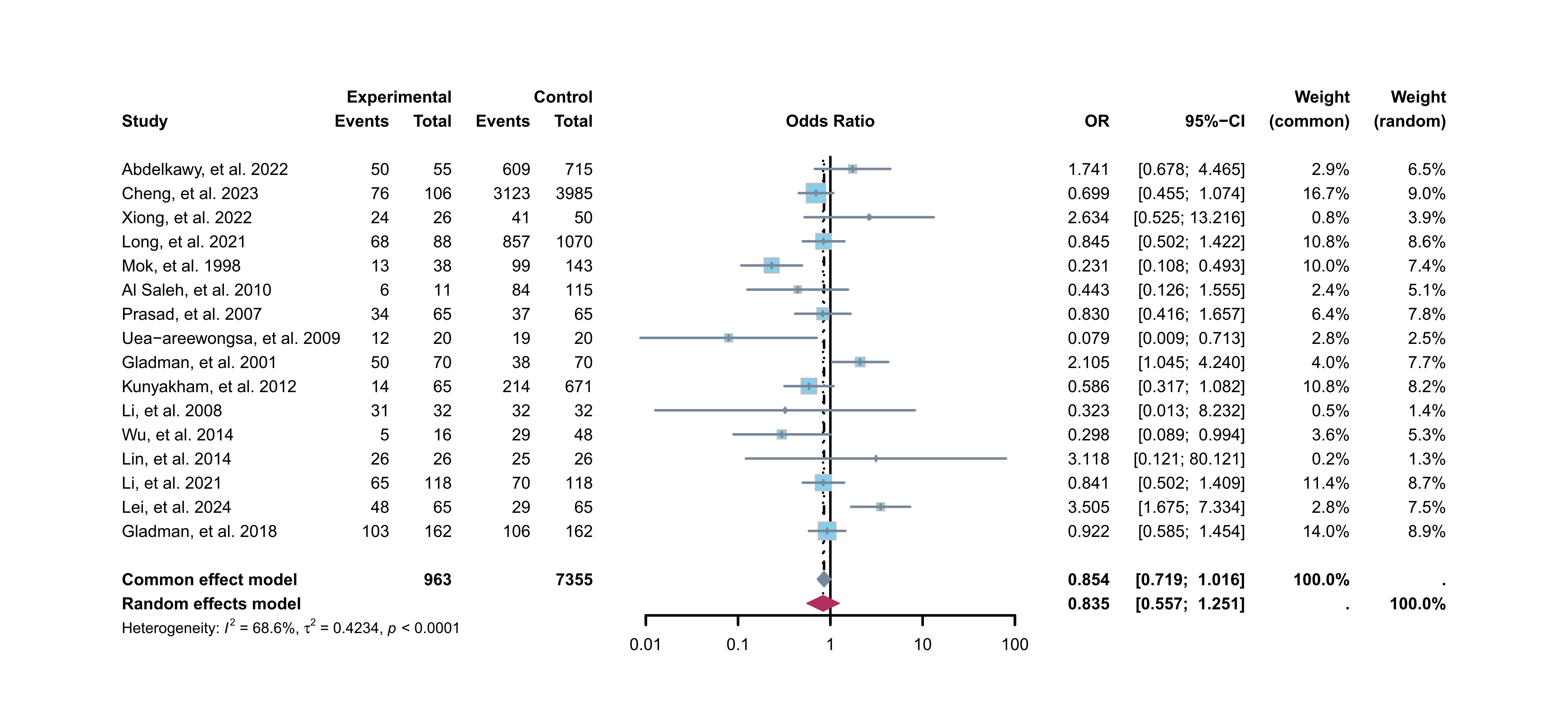

Supplement: Supplementary file 1 [file DataSheet1.zip › Supplementary Material/Supplementary figure 47.tif]

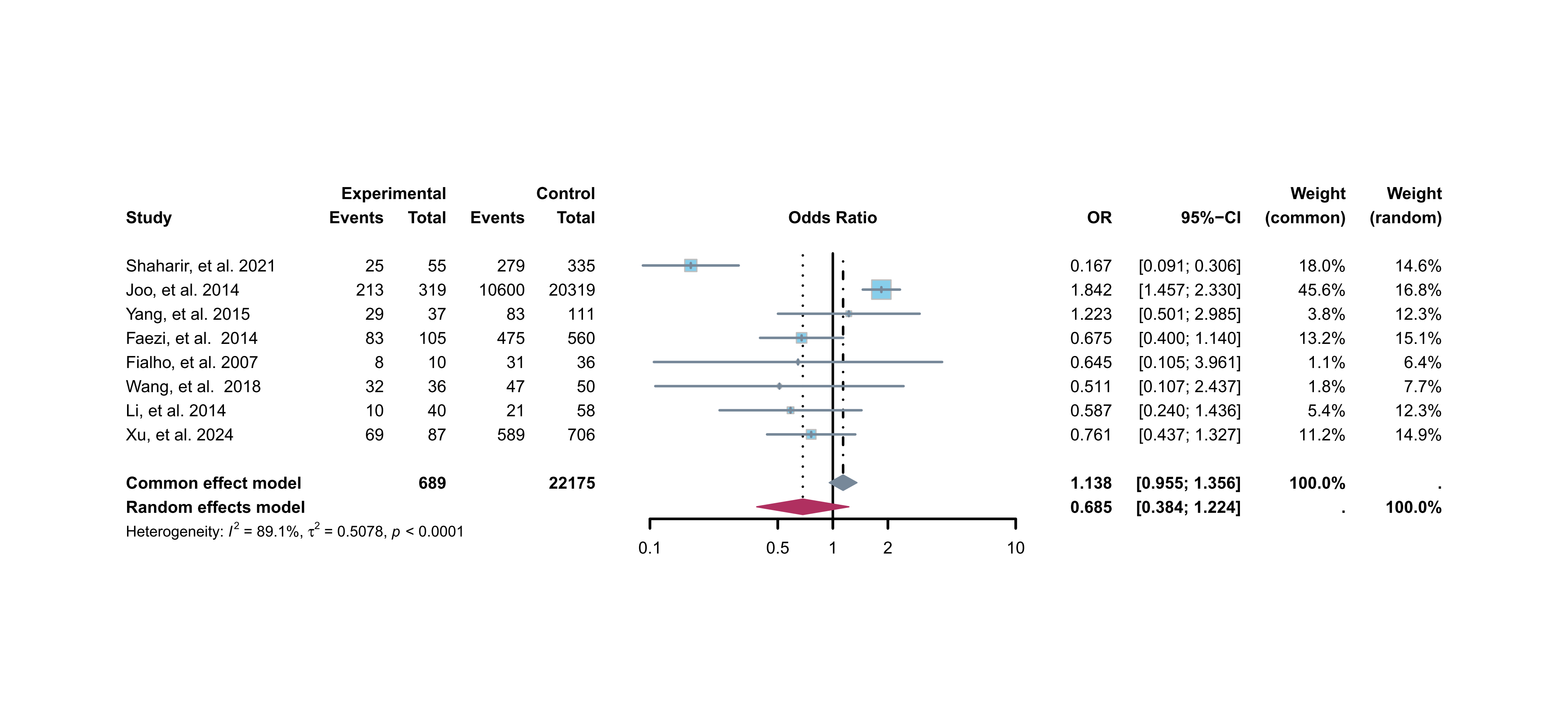

Supplement: Supplementary file 1 [file DataSheet1.zip › Supplementary Material/Supplementary figure 48.tif]

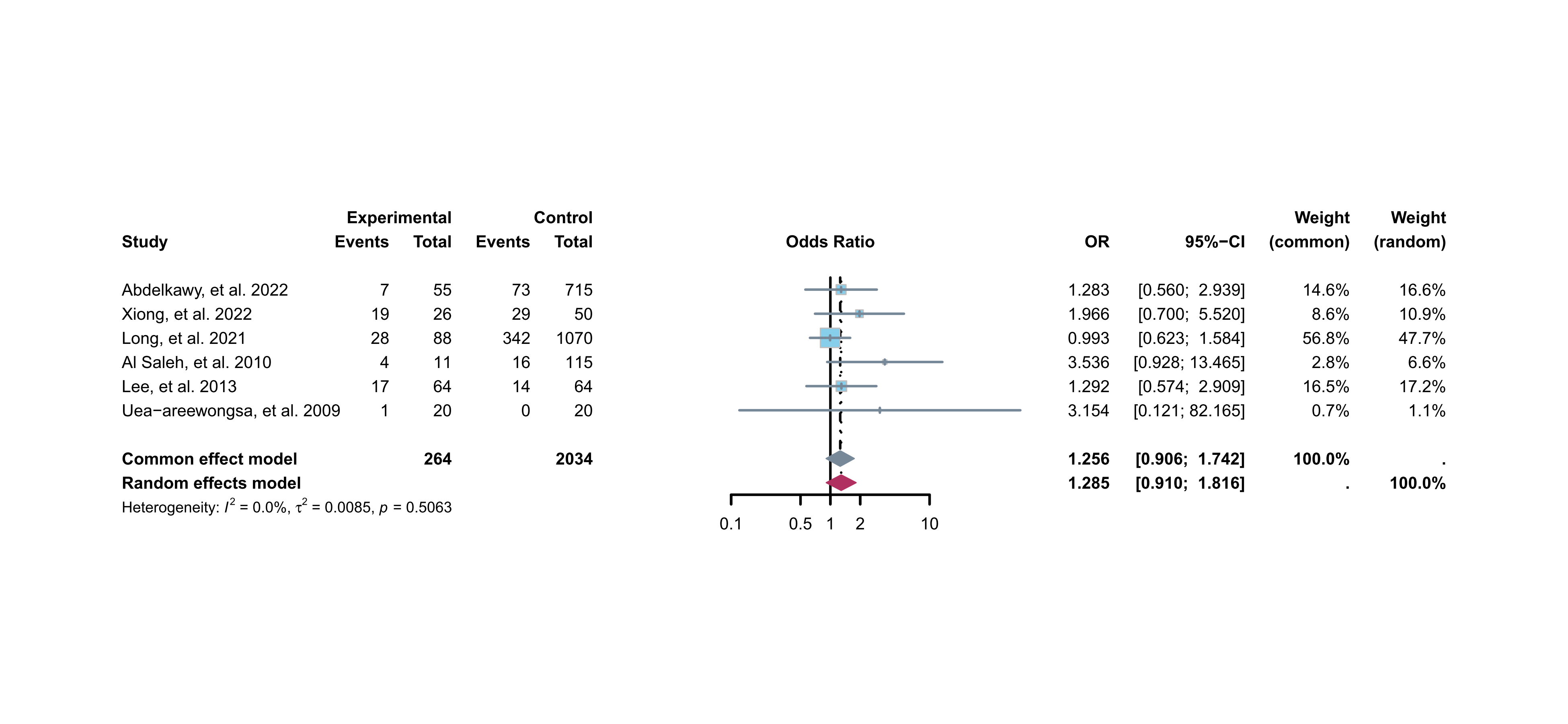

Supplement: Supplementary file 1 [file DataSheet1.zip › Supplementary Material/Supplementary figure 49.tif]

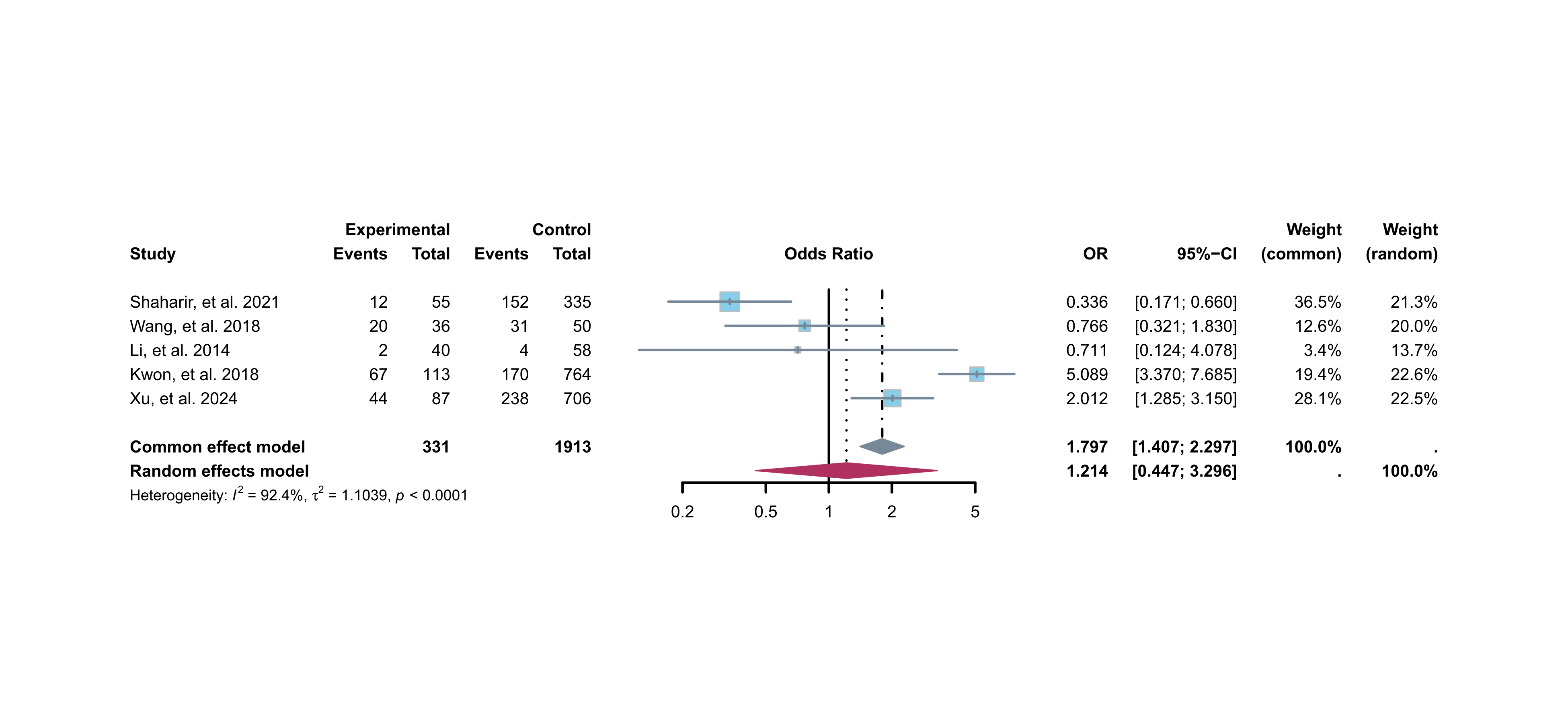

Supplement: Supplementary file 1 [file DataSheet1.zip › Supplementary Material/Supplementary figure 50.tif]

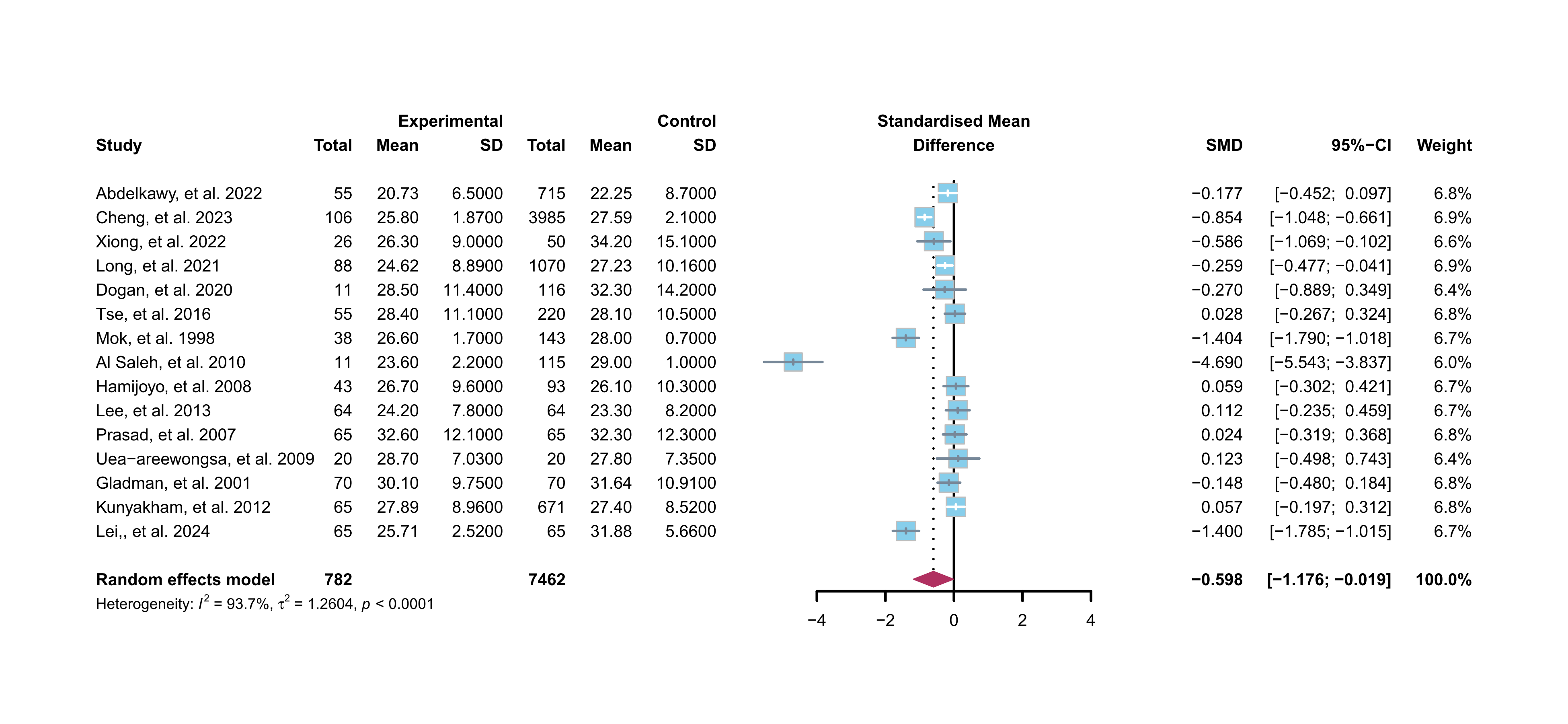

Supplement: Supplementary file 1 [file DataSheet1.zip › Supplementary Material/Supplementary figure 51.tif]

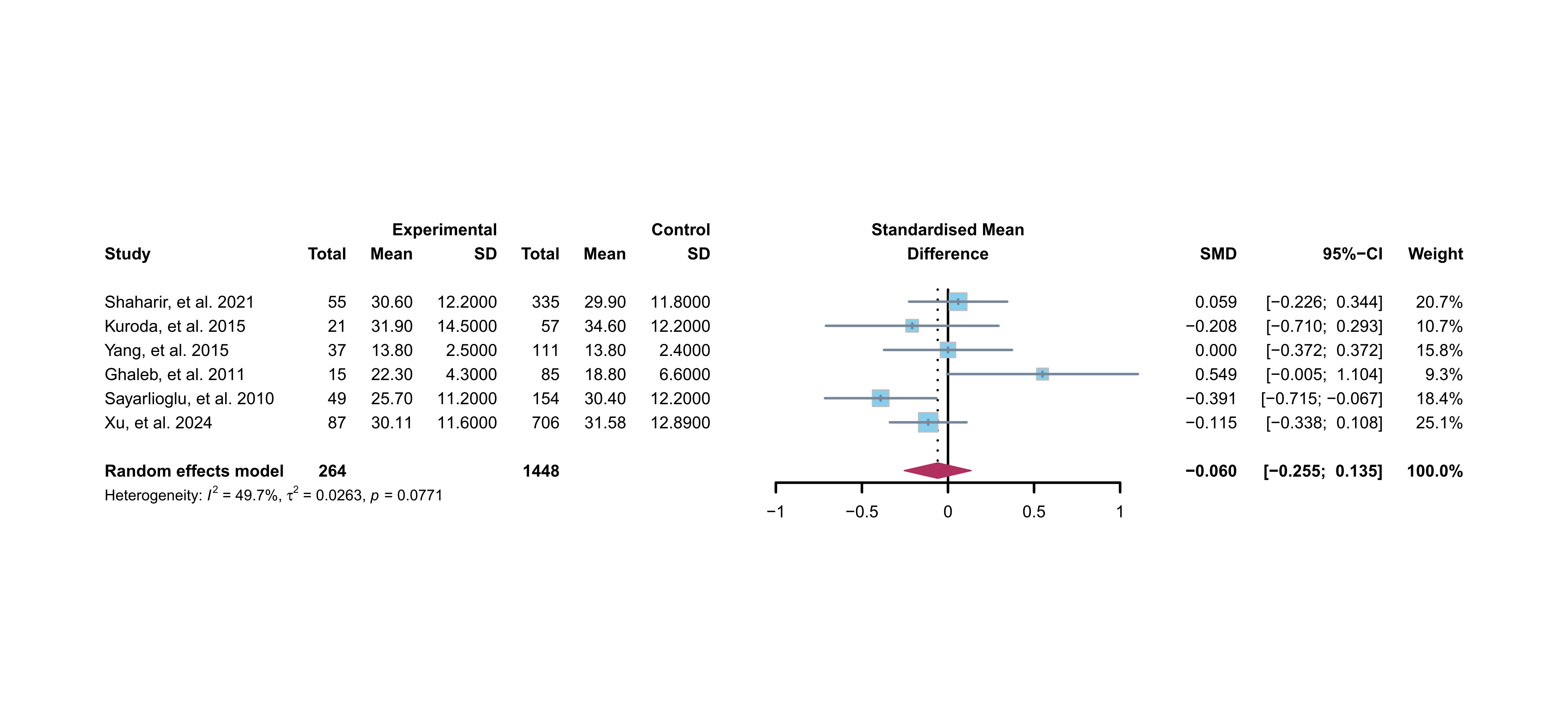

Supplement: Supplementary file 1 [file DataSheet1.zip › Supplementary Material/Supplementary figure 52.tif]

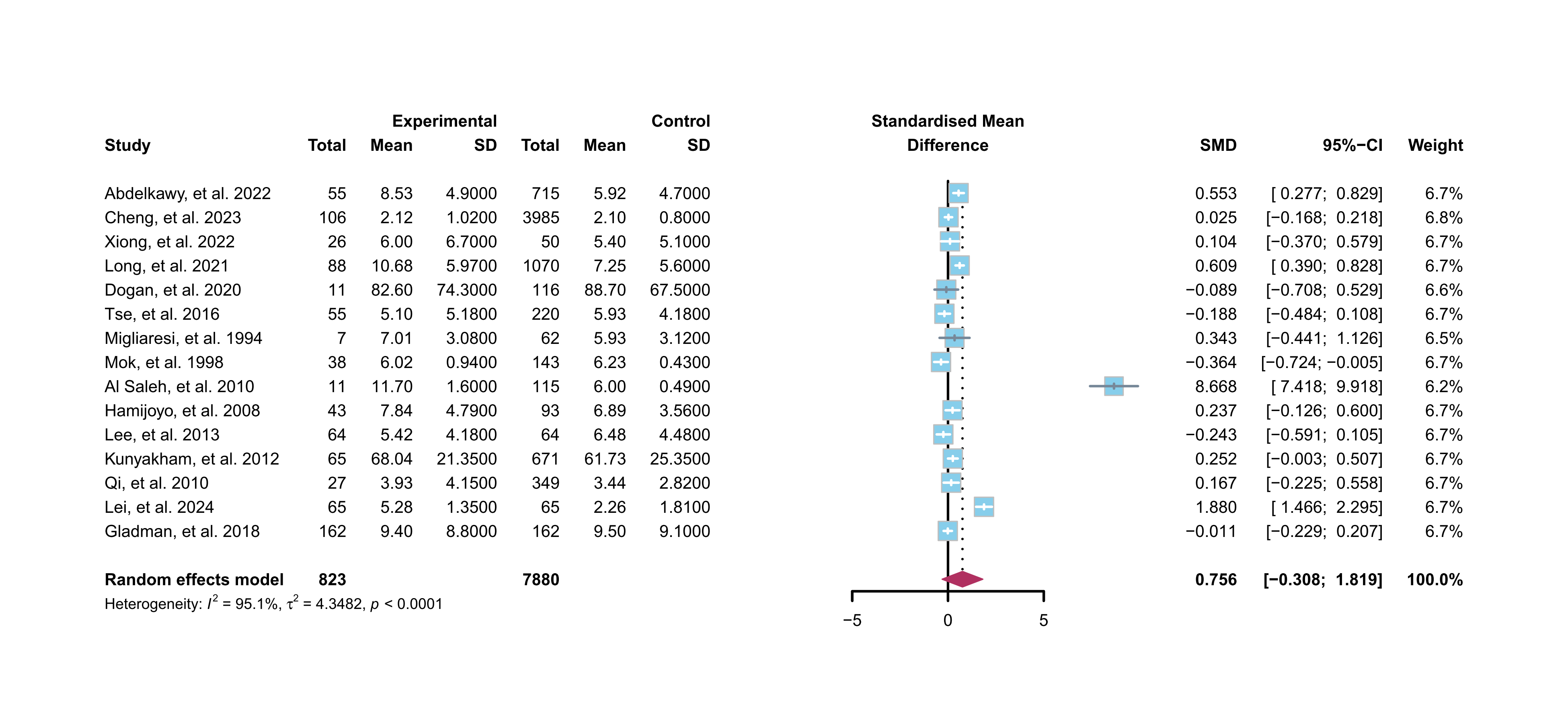

Supplement: Supplementary file 1 [file DataSheet1.zip › Supplementary Material/Supplementary figure 53.tif]

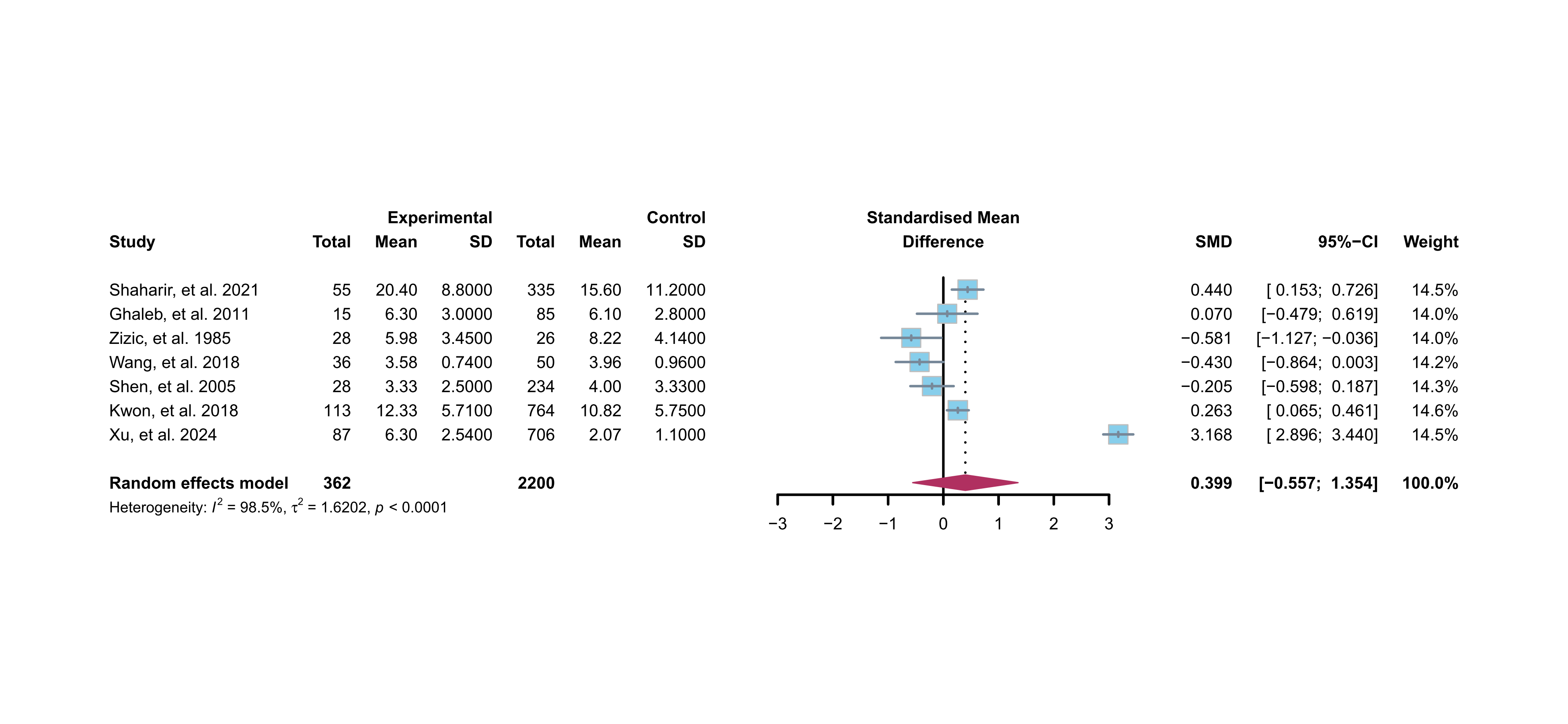

Supplement: Supplementary file 1 [file DataSheet1.zip › Supplementary Material/Supplementary figure 54.tif]

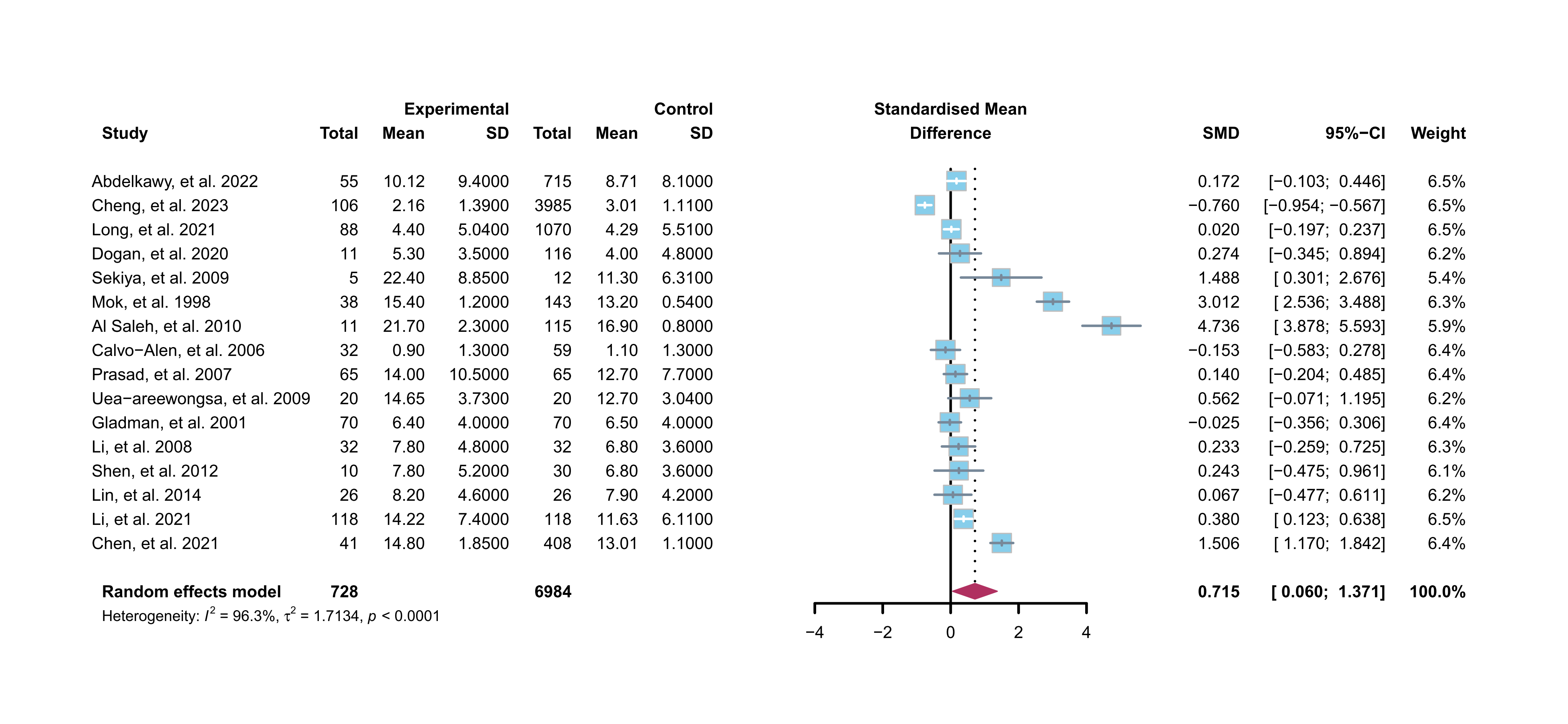

Supplement: Supplementary file 1 [file DataSheet1.zip › Supplementary Material/Supplementary figure 55.tif]

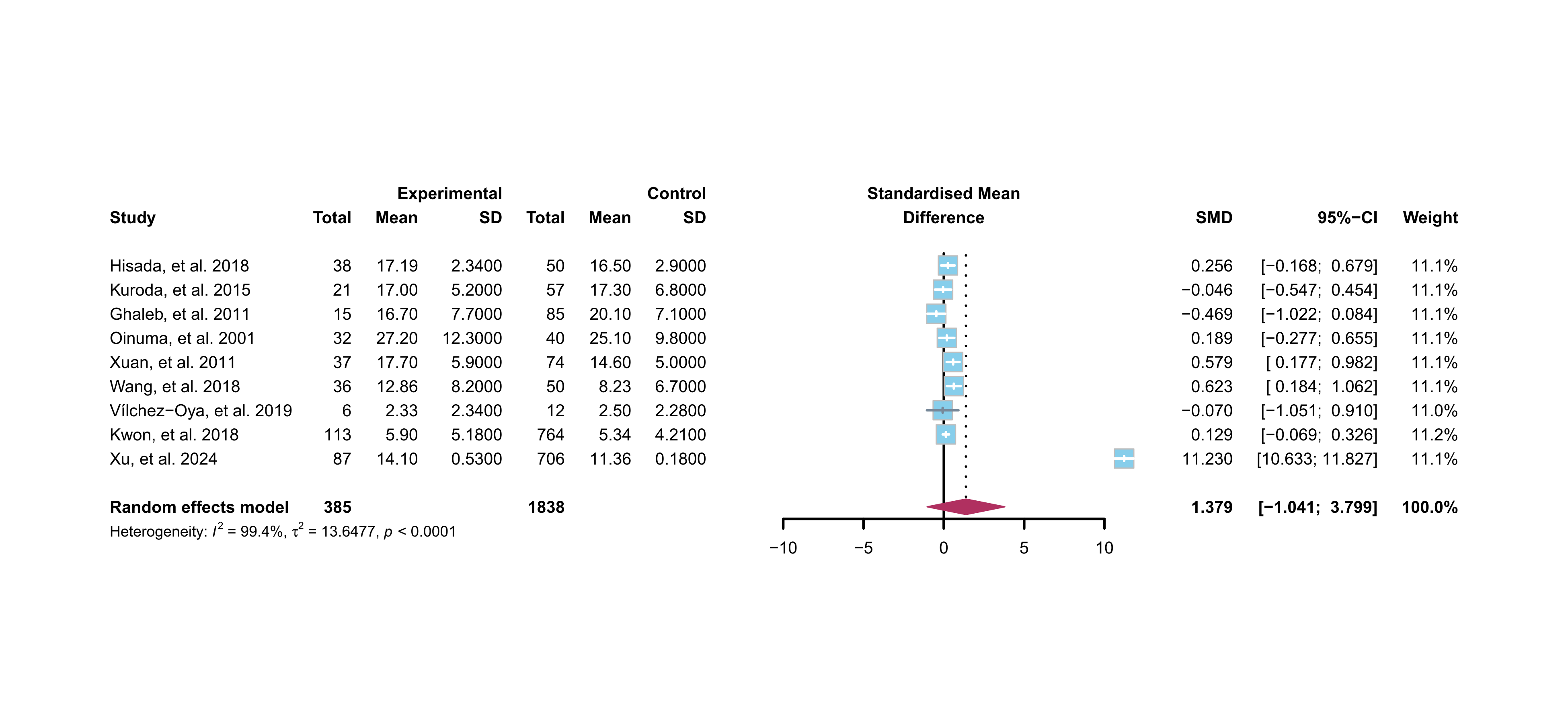

Supplement: Supplementary file 1 [file DataSheet1.zip › Supplementary Material/Supplementary figure 56.tif]
